# Supplementary material for: Axin2 coupled excessive Wnt‐glycolysis signaling mediates social defect in autism spectrum disorders
Source: EMBO Mol Med. 2023 Apr 20;15(6):e17101. doi: 10.15252/emmm.202217101 (PMC10245038; doi:10.15252/emmm.202217101)
Supplement: Supplementary file 1 — Expanded View Figures PDF [file EMMM-15-e17101-s013.pdf]

Expanded View Figures

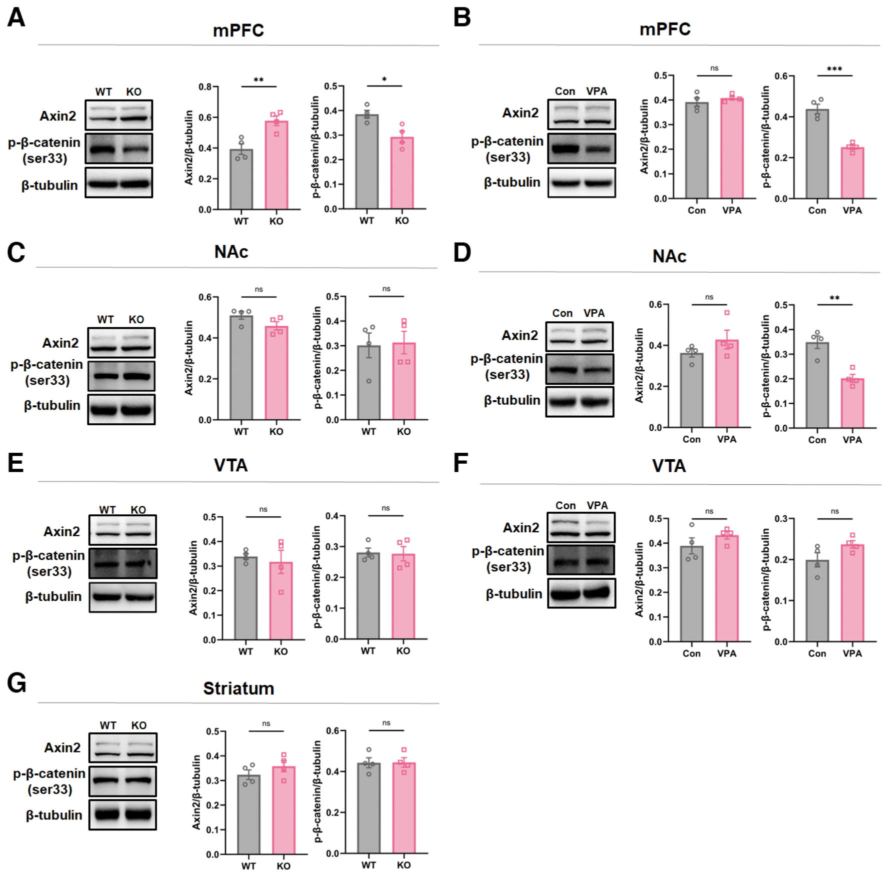

Figure EV1. Expression of Wnt signaling components in other brain regions.

- A Western blotting of Axin2 and p-β-catenin(S33) in the mPFC of WT and *Shank3*$^{-/-}$ mice.
- B Western blotting of Axin2 and p-β-catenin(S33) in the mPFC of control and VPA-treated mice.
- C Western blotting of Axin2 and p-β-catenin(S33) in the NAc of WT and *Shank3*$^{-/-}$ mice.
- D Western blotting of Axin2 and p-β-catenin(S33) in the NAc of control VPA-treated mice.
- E Western blotting of Axin2 and p-β-catenin(S33) in the VTA of WT and *Shank3*$^{-/-}$ mice.
- F Western blotting of Axin2 and p-β-catenin(S33) in the VTA of control VPA-treated mice.
- G Western blotting of Axin2 and p-β-catenin(S33) in the striatum of WT and *Shank3*$^{-/-}$ mice.

Data information: $N = 4$ samples from 12 mice per group. Mean ratio ± SEM. Two-tailed unpaired *t*-test (A, B, p-β-catenin in C, D-G). Mann–Whitney *U* test (Axin2 in C) *$P < 0.05$. **$P < 0.01$. ***$P < 0.001$. WT, wild type. KO, *Shank3*$^{-/-}$.
Source data are available online for this figure.

© 2023 The Authors

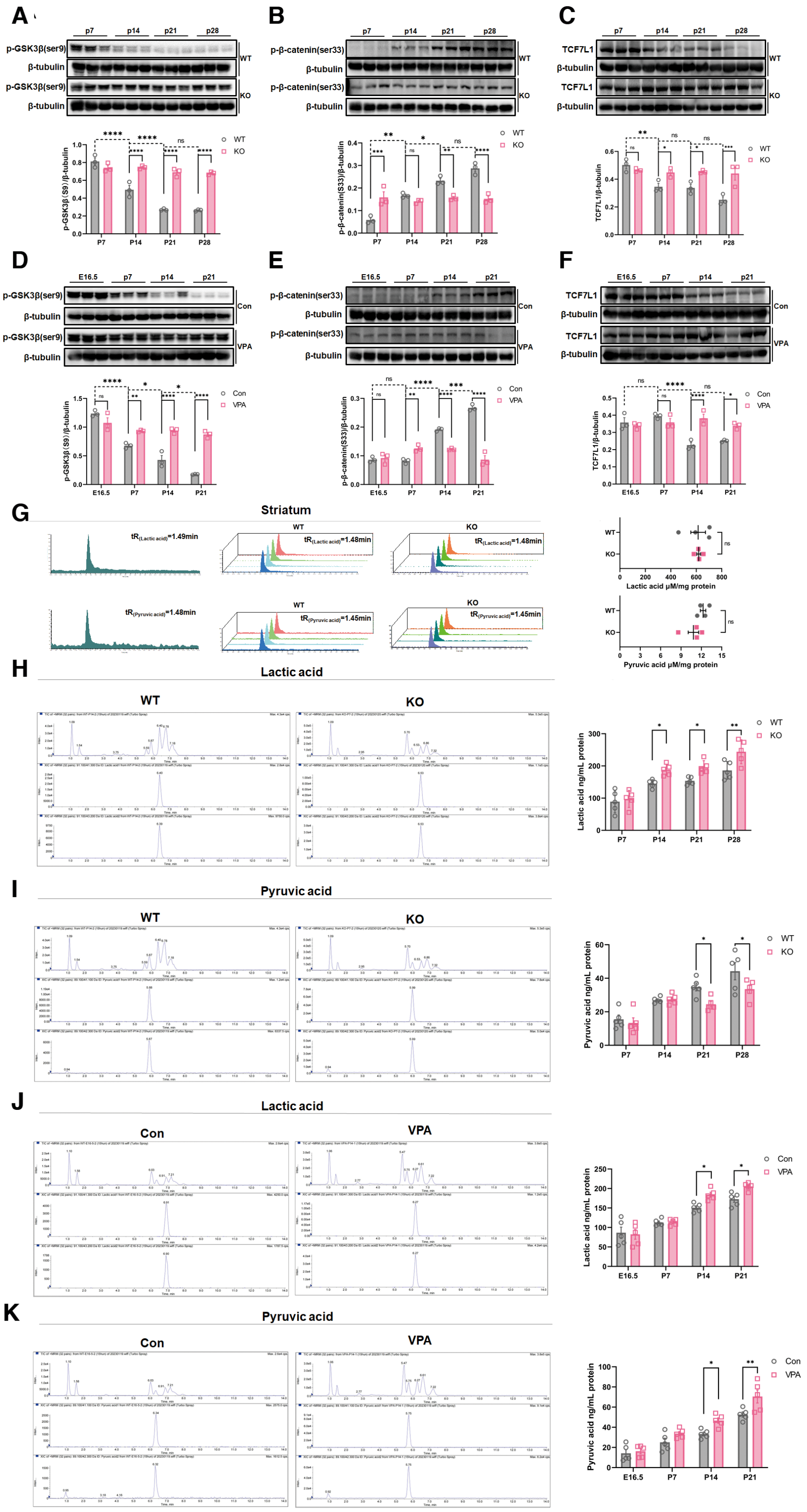

Figure EV2. Developmental changes of Wnt signaling and glycolysis.

A–C Western blotting of p-GSK3β(S9), p-β-catenin (S33) and TCF7L1 in the ACC of WT and *Shank3*$^{-/-}$ mice at P7, P14, P21 and P28.

D–F Western blotting of p-GSK3β(S9), p-β-catenin (S33) and TCF7L1 in the ACC of control and VPA-treated mice at E16.5, P7, P14 and P21.

G LS-MS measurement of lactic acid and pyruvic acid in the striatum of WT and *Shank3*$^{-/-}$ mice.

H, I LS-MS/MS measurement of lactic acid and pyruvic acid in the ACC of WT and *Shank3*$^{-/-}$ mice at P7, P14, P21 and P28.

J, K LS-MS/MS measurement of lactic acid and pyruvic acid in the ACC of control and VPA-treated mice at E16.5, P7, P14 and P21.

Data information: $N = 4$ samples from 12 mice per group in (A-F), 4 samples from 16 mice per group in (G), 5 samples from 20 mice per group in (H-K). Mean ratio ± SEM. Two-way repeated measurement ANOVA and Sidak's multiple comparisons test (A–F, H–K). Two-tailed unpaired *t*-test (G). *$P < 0.05$, **$P < 0.01$, ***$P < 0.001$, ****$P < 0.0001$. WT, wild type. KO, *Shank3*$^{-/-}$. Con, control.
Source data are available online for this figure.

 © 2023 The Authors

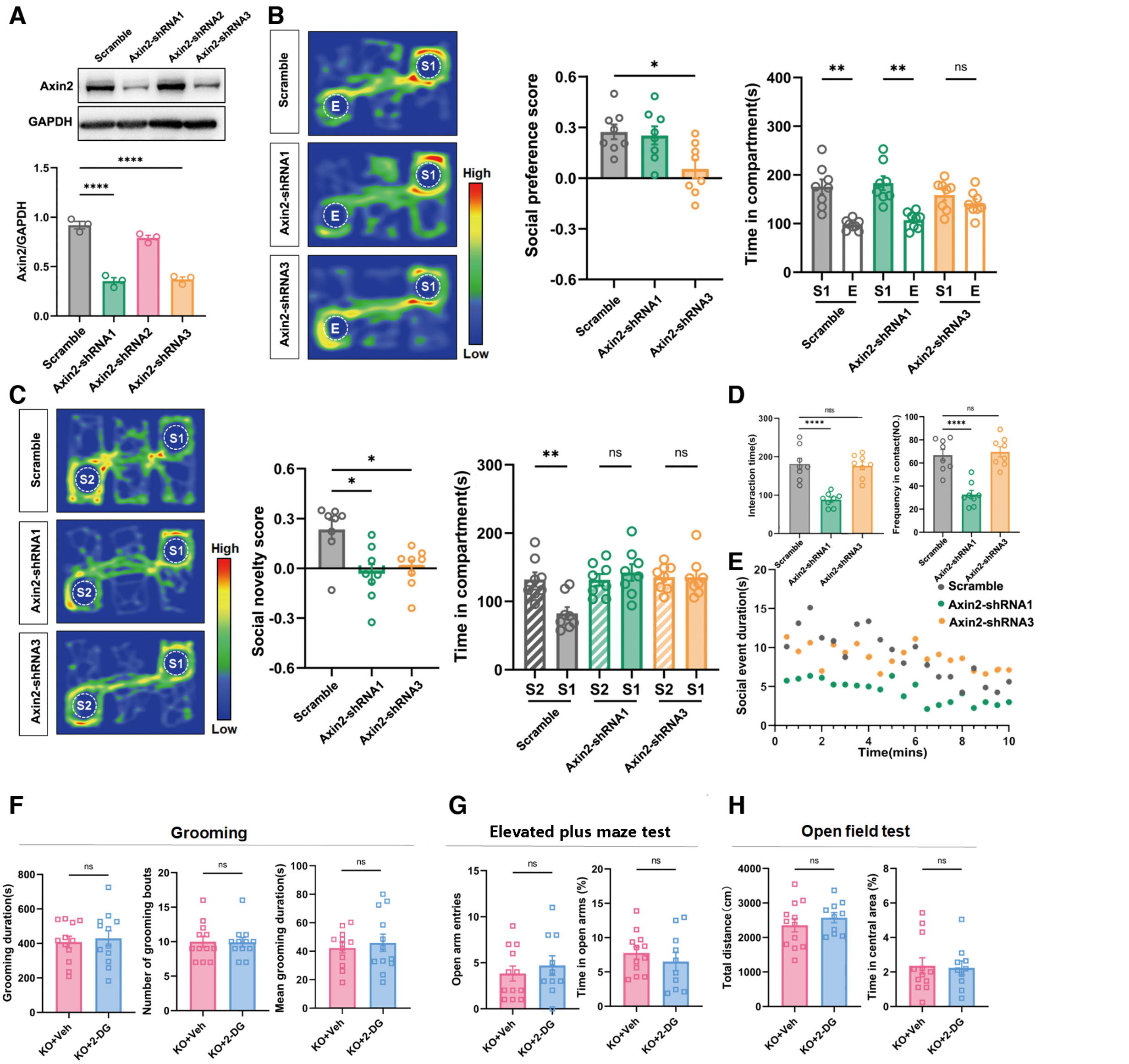

Figure EV3. Effects of knocking down Axin2 in on the social behavior of WT mice and 2-DG treatment on repetitive/anxiety-like behaviors of *Shank3*$^{-/-}$ mice.

A Western blotting verification of 3 Axin2-shRNA in ACC. Axin2-shRNA1 and Axin2-shRNA3 are efficient in knocking down Axin2.

B–E 3-chamber and resident-intruder assay of mice treated with scrambled RNA, Axin2-shRNA1 and Axin2-shRNA3. Notice the social impairment effects of Axin2-shRNA1 and Axin2-shRNA3.

F Grooming test of *Shank3*$^{-/-}$ mice treated with or without 2-DG.

G, H Elevated plus maze test and open-field test of *Shank3*$^{-/-}$ mice treated with or without 2-DG.

Data information: $N = 3$ mice in (A), 8 mice (B–D) and 10–12 mice (E–G) mice per group. Mean ratio ± SEM. One-way ANOVA with Tukey's multiple comparison test (A, D, social preference score in (B)). Kruskal–Wallis *H* test with Dunn's multiple comparison test (social novelty score in (C)). Paired *t*-test (time in compartment in Axin2-shRNA3 treated mice in (B), time in compartment in (C)). Wilcoxon signed-rank test (time in compartment of scrambled RNA and Axin2-shRNA3 treated mice in (B)). Two-tailed unpaired *t*-test and Mann–Whitney *U* test (F–H). *$P < 0.05$. **$P < 0.01$, ****$P < 0.0001$.
Source data are available online for this figure.

© 2023 The Authors

Figure EV4. Effects of XAV939 on the glycolysis/oxidative phosphorylation, social behavior, repetitive and anxiety-like behavior of ASD mice.

A ECAR assay of VPA-neurons treated with or without XAV939. Notice the decrease of glycolysis in XAV939-treated cells.
B OCR assay of VPA-neurons treated with or without XAV939. Notice the increase of oxidative phosphorylation in XAV939-treated cells.
C–E 3-chamber assay of *Shank3*$^{-/-}$ mice at 1 week following the last administration of XAV939. Notice the social improvement in XAV939-treated cells.
F–H Grooming test, elevated plus maze test and open-field test of *Shank3*$^{-/-}$ mice treated with or without XAV939.
I–K Grooming test, elevated plus maze test and open-field test of VPA-ASD mice treated with or without XAV939.

Data information: $N = 4$–5 batches of cells per group (A, B), 8 mice (D, E) and 7–8 mice (F–K) per group. Mean ratio ± SEM. Two-tailed unpaired *t*-test (max ECAR in B). Mann–Whitney *U* test (ECAR in (B), Open arm entries in (J) and (A)). $*P < 0.05$. $**P < 0.01$. Two-tailed unpaired *t*-test (social novelty score in E, F–K). Paired *t*-test (time in compartment in D and E). Wilcoxon signed-rank test (social preference scores in (E)). $*P < 0.05$, $**P < 0.01$.
Source data are available online for this figure.

© 2023 The Authors

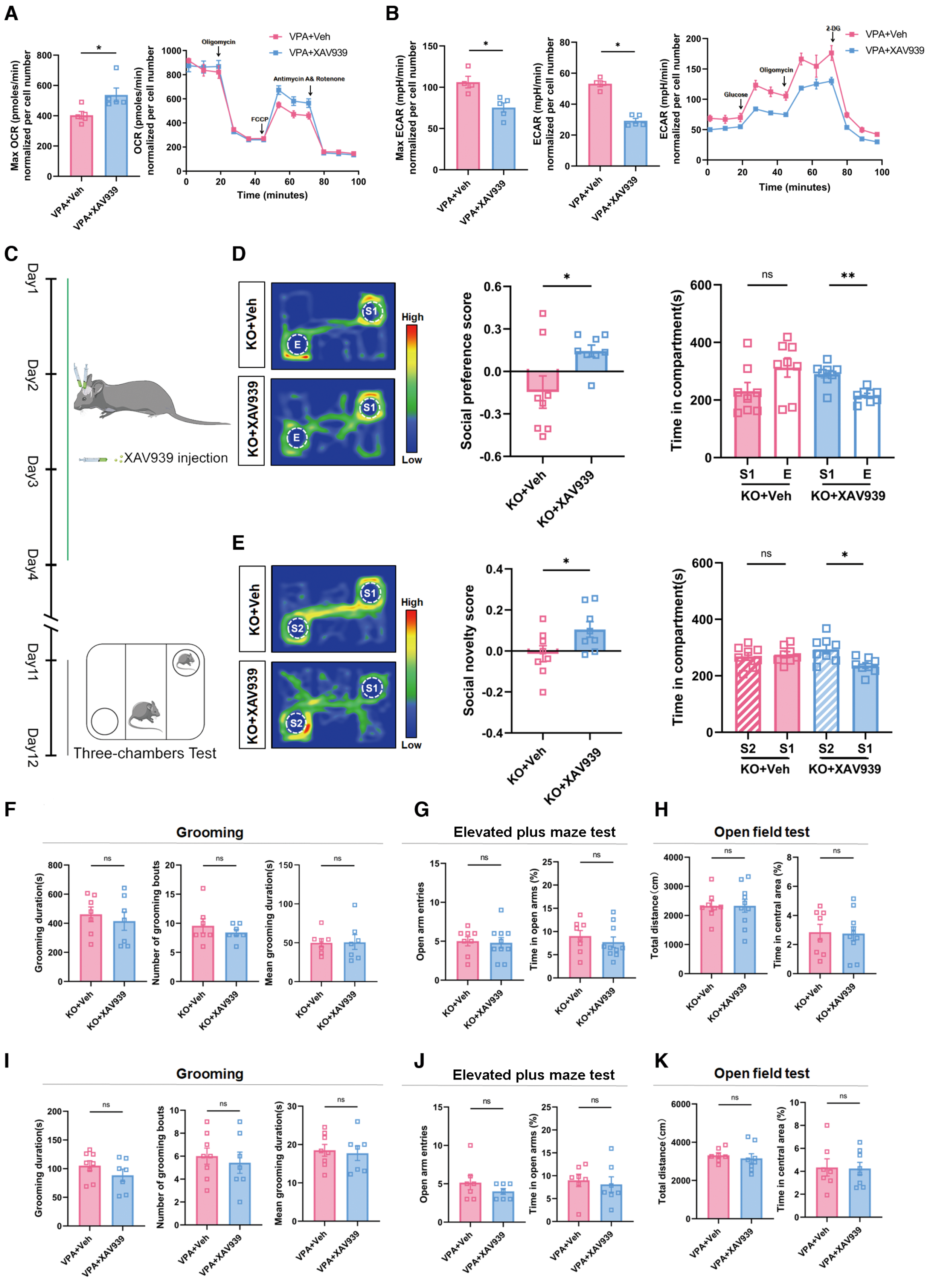
A
Max OCR (pmoles/min) normalized per cell number
OCR (pmoles/min) normalized per cell number
Oligomycin
FCCP
Antimycin A& Rotenone
VPA+Veh
VPA+XAV939
Time (minutes)
B
Max ECAR (mpH/min) normalized per cell number
ECAR (mpH/min) normalized per cell number
Glucose
Oligomycin
2-DG
C
Day1
Day2
Day3
Day4
Day11
Day12
XAV939 injection
Three-chambers Test
D
KO+Veh
KO+XAV939
High
Low
Social preference score
Time in compartment(s)
E
Social novelty score
F
Grooming
Grooming duration(s)
Number of grooming bouts
Mean grooming duration(s)
G
Elevated plus maze test
Open arm entries
Time in open arms (%)
H
Open field test
Total distance (cm)
Time in central area (%)
I
J
K

Figure EV4.

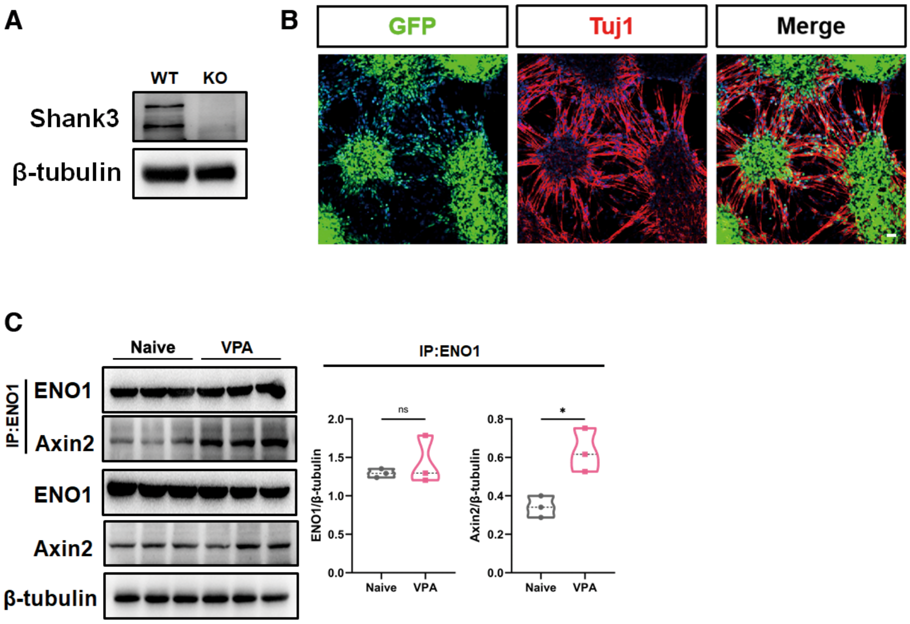

Figure EV5. Shank3 mutation and neural induction in human ESCs and interaction of Axin2/ENO1 in naïve and VPA-pretreated ESCs.

A Western blotting of Shank3 in WT and *Shank3* mutant human neurons.

B Immunocytochemistry of Tuj-1 in human ESCs after neural induction. At this stage, human neurons were used for experiments. Bar = 5 μm.

C Protein CO-IP of Axin2/ENO1 in naïve and VPA-treated human neurons.

Data information: $N = 3$ batches of cells. Two-tailed unpaired t-test. $^*P < 0.05$.
Source data are available online for this figure.

 © 2023 The Authors
