# Supplementary material for: Axin2 coupled excessive Wnt‐glycolysis signaling mediates social defect in autism spectrum disorders
Source: EMBO Mol Med. 2023 Apr 20;15(6):e17101. doi: 10.15252/emmm.202217101 (PMC10245038; doi:10.15252/emmm.202217101)
Supplement: Supplementary file 5 — PDF+ [file EMMM-15-e17101-s012.pdf]

Article

SOURCE DATA TRANSPARENT PROCESS OPEN ACCESS

EMBO Molecular Medicine

Axin2 coupled excessive Wnt-glycolysis signaling mediates social defect in autism spectrum disorders

Mengmeng Wang[1,†], Panpan Xian[1,†], Weian Zheng[1,2,†], Zhenzhen Li[1], Andi Chen[1], Haoxiang Xiao[1], Chao Xu[1], Fei Wang[1], Honghui Mao[1], Han Meng[1], Youyi Zhao[3], Ceng Luo[1], Yazhou Wang[1,*] & Shengxi Wu[1,**]

Abstract

Social dysfunction is the core syndrome of autism spectrum disorder (ASD) and lacks effective medicine. Although numerous risk genes and relevant environmental factors have been identified, the convergent molecular mechanism underlying ASD-associated social dysfunction remains largely elusive. Here, we report aberrant activation of canonical Wnt signaling and increased glycolysis in the anterior cingulate cortex (ACC, a key brain region of social function) of two ASD mouse models (*Shank3*$^{-/-}$ and valproic acid-treated mice) and their corresponding human neurons. Overexpressing β-catenin in the ACC of wild-type mice induces both glycolysis and social deficits. Suppressing glycolysis in ASD mice partially rescued synaptic and social phenotype. Axin2, a key inhibitory molecule in Wnt signaling, interacts with the glycolytic enzyme enolase 1 (ENO1) in ASD neurons. Surprisingly, an Axin2 stabilizer, XAV939, effectively blocked Axin2/ENO1 interaction, switched glycolysis/oxidative phosphorylation balance, promoted synaptic maturation, and rescued social function. These data revealed excessive neuronal Wnt-glycolysis signaling as an important underlying mechanism for ASD synaptic deficiency, indicating Axin2 as a potential therapeutic target for social dysfunction.

Keywords Axin2; glycolysis; social dysfunction; Wnt signaling
Subject Categories Metabolism; Neuroscience
DOI 10.15252/emmm.202217101 | Received 25 October 2022 | Revised 30 March 2023 | Accepted 3 April 2023 | Published online 20 April 2023
EMBO Mol Med (2023) 15: e17101

Introduction

Social dysfunction is the most predominant syndrome of autism spectrum disorders (ASD), which severely affects the life quality and career development of suffered patients, and lacks effective treatment (Anagnostou, 2018; Sullivan & Geschwind, 2019; Zhou *et al*, 2020). Last decade has witnessed the identification of approximately 1,000 risk genes and numerous environmental factors involved in the development of ASD (Newschaffer *et al*, 2007; Herbert, 2010; Iakoucheva *et al*, 2019). Elucidating the convergent mechanism is thought to be vital for developing effective treatment, which can benefit more patients. Electrophysiological studies have demonstrated that synaptic dysfunction, specifically the impairment of excitatory synaptic transmission or imbalance of excitatory-inhibitory ratio of synaptic transmission, was the major pathological change in multiple ASD mouse models and may account for social dysfunction (Yizhar *et al*, 2011; Antoine *et al*, 2019; Sohal & Rubenstein, 2019). However, whether there exists a convergent molecular mechanism for this social dysfunction-associated synaptic deficits still remains elusive.

Clinical studies frequently detected metabolic abnormalities in ASD patients. Mitochondrial dysfunction was found in blood samples of 30–80% ASD patients by different research groups (Rossignol & Frye, 2012; Frye, 2020). Biomarker studies reported increased lactate/pyruvate ratio in the serum of ASD patients (Vallee & Vallee, 2018; El Fotoh *et al*, 2019). In addition, ketogenic diet appears to improve ASD symptoms (Mu *et al*, 2020; Li *et al*, 2021), suggesting that glycolysis may be affected in ASD patients.

Aside from metabolic abnormalities, mutation of Wnt signaling components has also been suggested as highly detectable in ASD patients. For example, microdeletion and microduplication of multiple Wnt signaling components were found in ASD patients

1 Department of Neurobiology and Institute of Neurosciences, School of Basic Medicine, Fourth Military Medical University, Xi'an, China
2 School of Life Science and Research Center for Natural Peptide Drugs, Shaanxi Engineering and Technological Research Center for Conversation and Utilization of Regional Biological Resources, Yanan University, Yanan, China
3 State Key Laboratory of Military Stomatology and National Clinical Research Center for Oral Diseases and Shaanxi Engineering Research, Center for Dental Materials and Advanced Manufacture, Department of Anesthesiology, School of Stomatology, Fourth Military Medical University, Xi'an, China
*Corresponding author. Tel: +86 29 84774562; E-mail: yazhouw@fmmu.edu.cn
**Corresponding author. Tel: +86 29 84712311; E-mail: shengxi@fmmu.edu.cn
[†]These authors contributed equally to this work

© 2023 The Authors. Published under the terms of the CC BY 4.0 license

(Kalkman, 2012). A patient-based exome sequencing study mapped 39% of the most disruptive or severe *de novo* mutations to the β-catenin-related network (Sanders *et al*, 2012). Recent network study of ASD patients suggested that Wnt-β-catenin signaling, RAS–ERK, and PI3K-AKT are the three major abnormal signaling pathways for the development of ASD (Gazestani *et al*, 2019).

Given that Wnt signaling plays important roles in multiple aspects of neural development and that synaptic transmission requires high energy supply (Noelanders & Vleminckx, 2017; Devine & Kittler, 2018), it is possible that Wnt signaling and related metabolic abnormality could be involved in the synaptic deficits of ASD neurons. In cancer cells, Wnt signaling contributes to the proliferation of tumor cells via driving Warburg effect (aerobic glycolysis; Pate *et al*, 2014). Whether abnormal Wnt-glycolysis signaling occurs in ASD neurons and participates in the ASD social dysfunction remains still open.

In the present study, we addressed this question by using two widely adopted ASD models (*Shank3*$^{-/-}$ and valproic acid (VPA)-induced mice) and their corresponding human neurons. Our data disclosed an excessive neuronal Wnt-glycolysis signaling in synaptic deficits and their roles in social dysfunction and surprisingly revealed Axin2 as a convergent point of Wnt signaling and glycolysis activity in ASD neurons, indicating it as a potential therapeutic target for improving social function.

Results

Overactivation of canonical Wnt signaling in the ACC neurons of *Shank3*$^{-/-}$ and VPA-treated mice

We first adopted a widely accepted ASD model, *Shank3*$^{-/-}$ mice, to examine whether Wnt signaling was changed in social-related brain regions. Considering that Shank3 is a scaffold protein, we performed immunoprecipitation-mass spectrometry in the ACC, a major brain region in charge of ASD social function (Guo *et al*, 2019), of wild-type (WT) mice to explore if Shank3 interacted with any intracellular Wnt signaling components. Gene set enrichment analysis (GSEA) of differently immunoprecipitated proteins by anti-Shank3 antibody showed that Wnt signaling was among the top 3 signaling pathways with which Shank3 may interact (Fig 1A). Among significantly enriched Wnt-related proteins, several core Wnt signaling molecules, such as β-catenin and GSK3β, were found (Fig 1B). Protein co-immunoprecipitation confirmed the interaction between Shank3 and β-catenin under normal condition (Fig 1C). As nuclear accumulation of β-catenin is key for canonical Wnt signaling activation, we wondered whether Shank3/β-catenin interaction affected the nuclear transportation of β-catenin. Adding an anti-Shank3 antibody to primary cultured WT neurons induced rapid nuclear accumulation of β-catenin (Fig 1D). Western blotting confirmed the significantly higher levels of β-catenin in the nuclear proteins of *Shank3*$^{-/-}$ ACC neurons and lower levels of β-catenin in the membrane of *Shank3*$^{-/-}$ ACC neurons (Fig 1E). In addition, the levels of total β-catenin, p-GSK3β (S9, an inhibitory form of GSK3β), and Axin2 (a feedback inhibitory protein of Wnt signaling) increased significantly in primary *Shank3*$^{-/-}$ ACC neurons, as compared to WT neurons (Fig 1F). In the ACC of *Shank3*$^{-/-}$ mice, the inactive form of β-catenin (p-β-catenin, S33) and the Wnt signaling inhibitory peptide Sfrp1 were downregulated, while the Wnt signaling downstream transcription factor TCF1L1 upregulated (Fig 1G). These data indicated canonical Wnt signaling was overactivated in the ACC neurons of *Shank3*$^{-/-}$ mice.

Next, we assessed Wnt signaling in an environmental factor-induced ASD model, VPA-treated mice. Similar as in *Shank3*$^{-/-}$ mice, higher levels of p-GSK3β(S9), Axin2, and nuclear β-catenin were detected in the ACC of VPA-treated mice, as compared to those observed in control mice (Fig 1H). Therefore, canonical Wnt signaling is overactivated in the ACC of VPA-treated mice as well.

To test whether Wnt signaling was changed in other social-associated brain areas, we examined the expression of p-β-catenin (S33) and Axin2 in the medial prefrontal cortex (mPFC), ventral tegmental area (VTA), and nucleus accumbens (NAc). *Shank3*$^{-/-}$ mice showed significant increase of Axin2 and decrease of p-β-catenin (S33) in mPFC (Fig EV1A). VPA-treated mice showed notable downregulation of p-β-catenin(S33), but no changes of Axin2 in mPFC (Fig EV1B). Both mice showed no alteration of all these proteins in VTA and NAc (Fig EV1C–F). In addition, none of these proteins were found changed in the striatum of *Shank3*$^{-/-}$ mice (Fig EV1G). These data indicated that aberrant Wnt signaling occurs mainly in the ACC of *Shank3*$^{-/-}$ and VPA-treated mice.

We further explored when abnormal Wnt signaling appeared during development in these two ASD models. Considering that cortical *Shank3* transcription is detected from late neonatal and that VPA is usually administered at early embryonic stage (Wang *et al*, 2014), we compared the expression of key Wnt signaling molecules in the ACC of WT versus *Shank3*$^{-/-}$ mice from P7 to P28, and control versus VPA-treated mice from E16.5 to P21. *Shank3*$^{-/-}$ mice showed significant high levels of p-GSK3β(S9) and TCF7L1, and low levels of p-β-catenin(S33) starting from P14, in comparison with those at their corresponding time points of WT mice (Fig EV2A–C). VPA-treated mice showed similar changes of these proteins from P14, except for obvious increase of p-GSK3β(S9) starting from P7 (Fig EV2D–F). These data suggested that abnormal Wnt signaling in the ACC of these two ASD mice emerges from juvenile.

Elevation of glycolysis in ACC neurons of *Shank3*$^{-/-}$ and VPA-treated mice

We next evaluated the energy metabolism in the ACC of these two ASD mouse models by measuring the overall ATP levels. Significant lower ATP levels were detected in the ACC of *Shank3*$^{-/-}$ and VPA-treated mice (Fig 2A), indicating the dysregulation of metabolic homeostasis. Transcriptomic GSEA analysis indicated that glycolysis-associated genes were upregulated in the ACC of *Shank3*$^{-/-}$ mice (Fig 2B). Among the 12 significantly increased genes, enolase 1 (*ENO1*), alcohol dehydrogenase 1 (*ADH1*), and aldehyde dehydrogenase3 (*ALDH3*) showed the most obvious upregulation (Fig 2B and C), which was confirmed by western blotting (Fig 2D), indicating increased glycolysis in the ACC of *Shank3*$^{-/-}$ mice. To validate this altered glycolysis, we measured key metabolites of glycolysis by liquid chromatography–mass spectrometry (LC–MS). In the ACC of *Shank3*$^{-/-}$ mice, the levels of lactic acid significantly increased while those of pyruvic acid decreased (Fig 2E). In the striatum of *Shank3*$^{-/-}$ mice, both lactic acid and pyruvic acid were maintained at the similar levels with those in WT mice (Fig EV2G). As glycolysis is normally dominant in astrocytes

 © 2023 The Authors

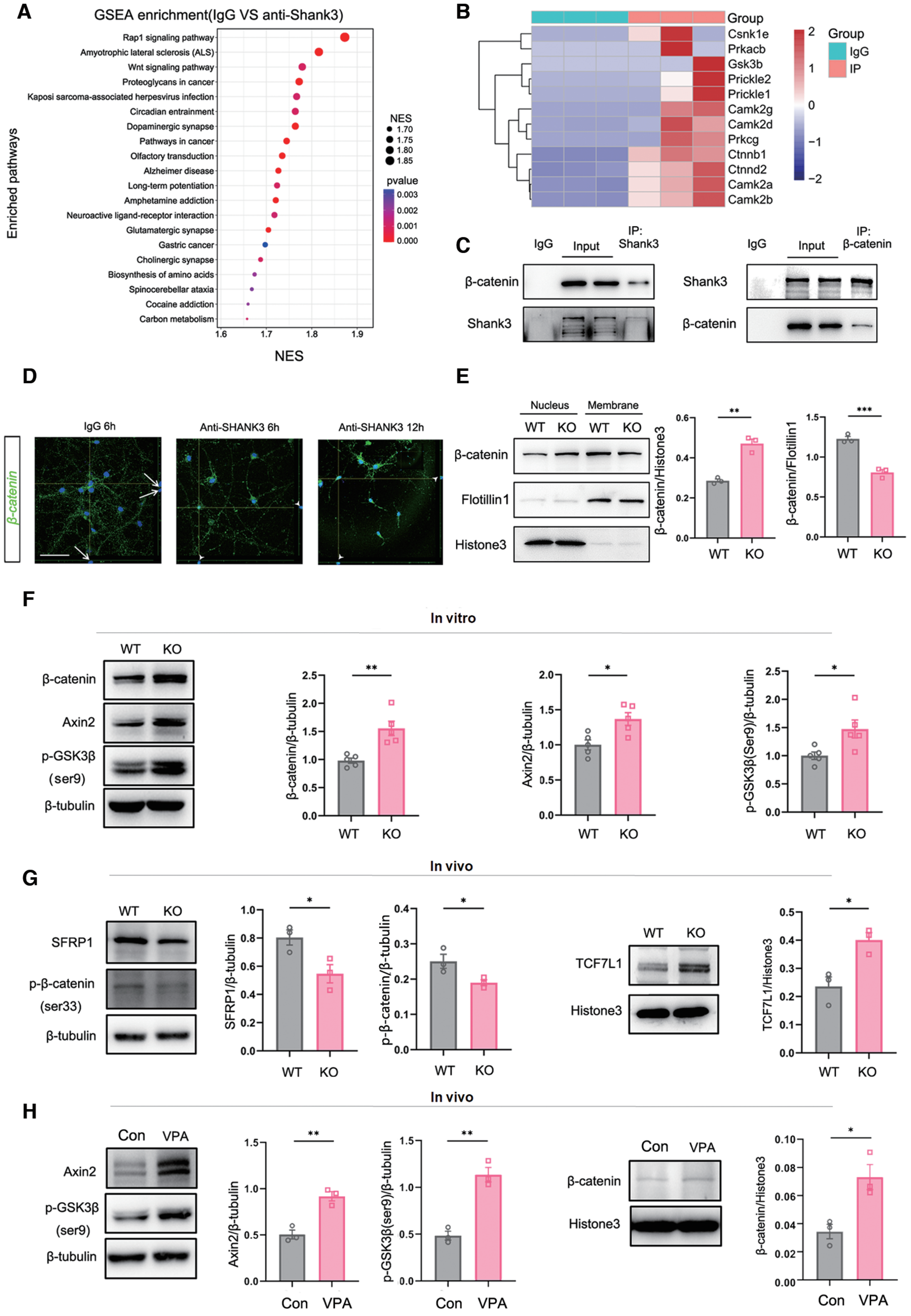
A
GSEA enrichment(IgG VS anti-Shank3)
Rap1 signaling pathway
Amyotrophic lateral sclerosis (ALS)
Wnt signaling pathway
Proteoglycans in cancer
Kaposi sarcoma-associated herpesvirus infection
Circadian entrainment
Dopaminergic synapse
Pathways in cancer
Olfactory transduction
Alzheimer disease
Long-term potentiation
Amphetamine addiction
Neuroactive ligand-receptor interaction
Glutamatergic synapse
Gastric cancer
Cholinergic synapse
Biosynthesis of amino acids
Spinocerebellar ataxia
Cocaine addiction
Carbon metabolism
Enriched pathways
NES
pvalue
B
Group
Csnk1e
Prkacb
Gsk3b
Prickle2
Prickle1
Camk2g
Camk2d
Prkcg
Ctnnb1
Ctnnd2
Camk2a
Camk2b
IgG
IP
C
IgG
Input
IP: Shank3
β-catenin
Shank3
IP: β-catenin
D
IgG 6h
Anti-SHANK3 6h
Anti-SHANK3 12h
β-catenin
E
Nucleus
Membrane
WT KO WT KO
Flotillin1
Histone3
β-catenin/Histone3
β-catenin/Flotillin1
F
In vitro
Axin2
p-GSK3β (ser9)
β-tubulin
β-catenin/β-tubulin
Axin2/β-tubulin
p-GSK3β(Ser9)/β-tubulin
G
In vivo
SFRP1
p-β-catenin (ser33)
SFRP1/β-tubulin
p-β-catenin/β-tubulin
TCF7L1
TCF7L1/Histone3
H
Con VPA
p-GSK3β(ser9)/β-tubulin
β-catenin/Histone3

Figure 1.

◀ **Figure 1. Wnt activation in ACC neurons of *Shank3*$^{-/-}$ and VPA-treated mice.**

A GSEA enrichment of significantly changed signaling pathways in pull-down proteins by anti-Shank3 antibody versus IgG. Wnt signaling is among the top 3 enriched pathways.
B Heat map of GSEA enriched Wnt signaling components.
C Protein CO-IP of Shank3 and β-catenin.
D Immunocytochemistry of β-catenin in primary WT neurons treated with anti-Shank3 antibody or IgG. Notice the nuclear accumulation of β-catenin upon anti-Shank3 antibody treatment. Arrows point to β-catenin-negative nucleus. Arrowheads point to β-catenin-positive nucleus.
E Western blotting of β-catenin in the membrane and nuclear protein of WT and *Shank3*$^{-/-}$ neurons. Notice the higher levels of β-catenin in the nuclear protein of *Shank3*$^{-/-}$ neurons.
F Western blotting of β-catenin, Axin2 and p-GSK3β(S9) in WT or *Shank3*$^{-/-}$ primary neurons.
G Western blotting of SFRP1 and p-β-catenin(S33) in the total proteins, and TCF7L1 in the nuclear protein of WT or *Shank3*$^{-/-}$ ACC.
H Western blotting of Axin2 and p-GSK3β(S9) in the total proteins, and β-catenin(S33) in the nuclear protein of ACC of WT or VPA-treated mice.

Data information: Bar = 50 μm in (D). N = 15 in (A, B), 3 in (E, G, H) mice and 5 batches of cells (F) per group. Mean ratio ± SEM. Two-tailed unpaired t-test. *P < 0.05. **P < 0.01. ***P < 0.001. WT, wild type. KO, *Shank3*$^{-/-}$ mice. Con, control.
Source data are available online for this figure.

(Bonvento & Bolanos, 2021), we further tested whether glycolysis occurred in primary neurons of *Shank3*$^{-/-}$ mice by evaluating the extracellular acidification rate (ECAR). In comparison with WT neurons, *Shank3*$^{-/-}$ neurons showed significantly higher glycolytic activity upon glucose stimulation. After oligomycin addition, *Shank3*$^{-/-}$ neurons exhibited significantly higher glycolytic capacity (Fig 2F).

In the ACC of VPA-treated mice, both lactic acid and pyruvic acid were higher, relative to those in control mice (Fig 2G). In comparison with WT neurons, primary neurons derived from VPA-treated mice showed significantly higher glycolytic activity upon glucose stimulation (Fig 2H). Together, these data demonstrated increased glycolysis in the ACC neurons of *Shank3*$^{-/-}$ and VPA-treated mice.

Next, we assessed the developmental changes of glycolytic metabolites in the ACC of these two mice. In comparison with those in WT mice, the levels of lactic acid in *Shank3*$^{-/-}$ and VPA-treated mice raised starting from P14 (Figs EV2H and 2J). The levels of pyruvic acid in the ACC of *Shank3*$^{-/-}$ and VPA-treated mice showed similar trends of alteration as observed in adult starting from P21 and P14, respectively, as compared to those in control mice at their corresponding time points (Figs EV2I and 2K).

Wnt signaling overactivation in ACC induces glycolysis and impairs social function in WT mice

We next asked whether overactivating Wnt signaling in WT ACC neurons was sufficient for inducing glycolysis and social dysfunction. We used β-cateninEX3$^{loxp/+}$ mice (β-catEX3) in which the exon3 of β-catenin is floxed by loxp sites and injected AAV-CaMKII-Cre into the ACC (Fig 3A). As phosphorylation of β-catenin's exon 3 is essential for its degradation, Cre-expressing cells expressing a stabilized β-catenin overactivate Wnt signaling. Three weeks after virus injection, Golgi staining was performed for evaluating dendritic and synaptic changes (Fig 3B). The spine density of ACC pyramidal neurons decreased remarkably in CaMKII-Cre-treated mice (Fig 3C). With respect to spine subtypes, mushroom-like and stubby spines were affected obviously (Fig 3D). Sholl analysis showed significantly less basal but not apical dendrite branches in CaMKII-Cre-treated mice (Fig 3E and F). Patch-clamp recording showed that both the frequency and average amplitude of miniature excitatory postsynaptic currents (mEPSCs) in CaMKII-Cre-treated ACC were markedly smaller than those of control mice (Fig 3G and H). In the 3-chamber assay, β-catenin overexpressing mice exhibited significant reduction of social preference while no significant change in social novelty (Fig 3I). In the resident-intruder assay, β-catenin overexpressing mice exhibited much less touch with intruder mice (Fig 3J and K). These data indicated that overactivating Wnt signaling in ACC neurons is sufficient for inducing synaptic and social deficits.

To confirm the effects of Wnt overactivation on social function, we knocked down the expression of Axin2, which is a feedback inhibitory protein of Wnt signaling, by injecting AAV-Axin2-shRNA into the ACC of WT mice. Two out of three Axin2-shRNAs showed efficient Axin2 knockdown in the ACC (Fig EV3A). Injecting either of those in the ACC significantly reduced social novelty or social ability of mice (Fig EV3B–E).

To investigate whether Wnt overactivation could affect glycolysis in neurons, we crossed CaMKII-Cre mice with β-catEX3 mice and cultured neurons from CaMKII-Cre:β-catEX3 embryos. The ECAR assay showed that both the reserved and maximum glycolysis in CaMKII-Cre:β-catEX3 neurons were significantly higher than in control neurons (Fig 3L and M). These data indicated that overactivating Wnt signaling increases glycolysis in neurons.

Suppressing glycolysis attenuates synaptic and social deficits in ASD mice

We then investigated the outcome of blocking glycolysis on synaptic and social deficits by administering 2-deoxy-d-glucose (2-DG, a glucose mimic which inhibits glycolysis) to *Shank3*$^{-/-}$ mice. Although no significant change in dendritic branching and average dendritic length (both basal and apical) was found in the ACC of 2-DG treated *Shank3*$^{-/-}$ mice (Fig 4A–C), this treatment significantly increased spine number, particularly that of filopodial, stubby, and mushroom-like spines in the ACC of *Shank3*$^{-/-}$ mice (Fig 4D–F). Western blotting showed significantly upregulated expression of ASD-associated synaptic scaffold protein Homer1, but not postsynaptic density protein 95 (PSD95), in the ACC of 2-DG-treated *Shank3*$^{-/-}$ mice (Fig 4G). Interestingly, 2-DG treatment significantly increased the expression of membrane GluR1 in the ACC of *Shank3*$^{-/-}$ mice but not the total GluR1 levels (Fig 4G and H), suggesting that rebalancing the energy supply may facilitate synaptic maturation. The 3-chamber and resident-intruder assays showed that 2-DG treatment significantly improved the social preference and

 © 2023 The Authors

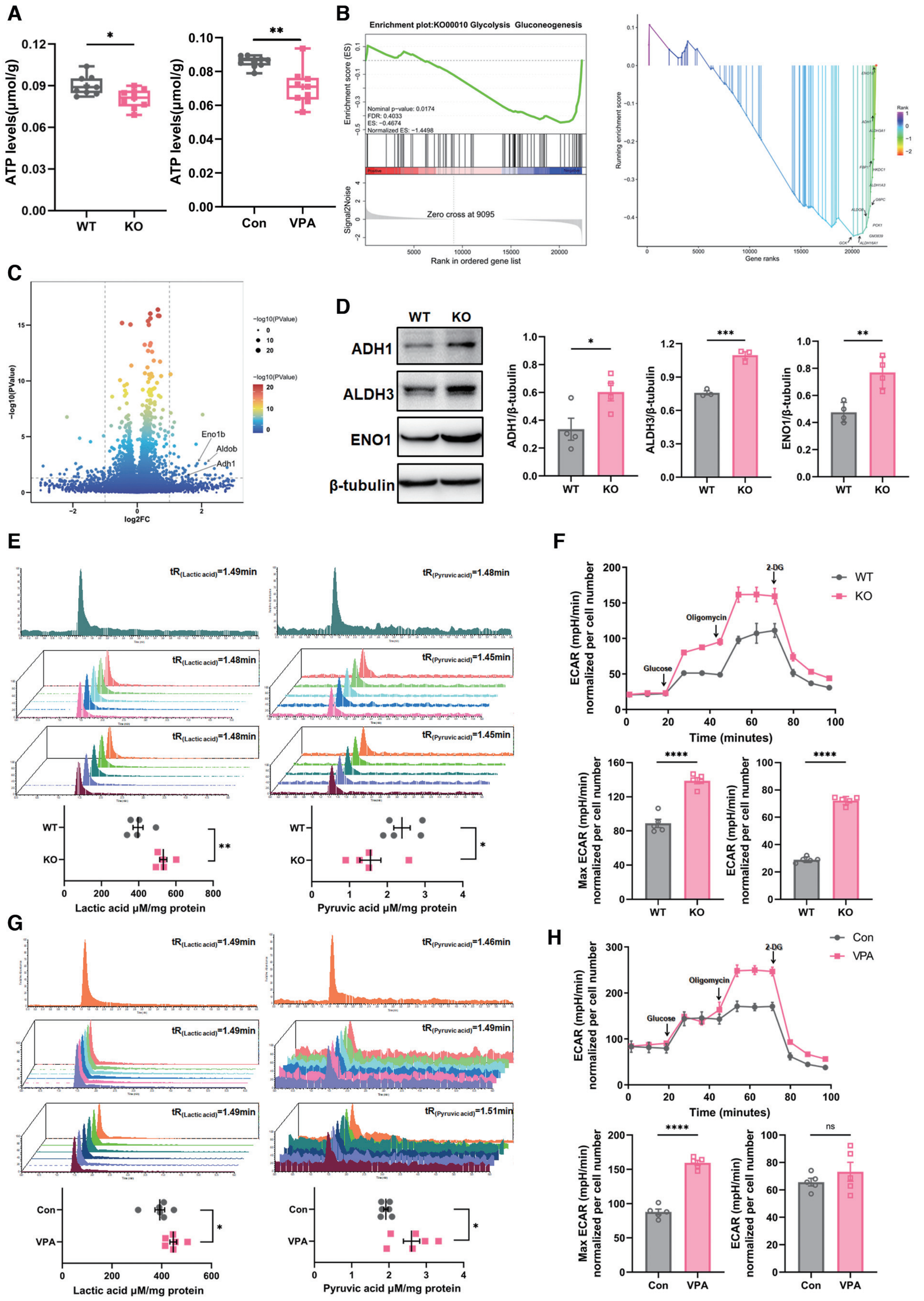
A
ATP levels(μmol/g)
WT
KO
Con
VPA
B
Enrichment plot:KO00010 Glycolysis Gluconeogenesis
Enrichment score (ES)
Nominal p-value: 0.0174
FDR: 0.4033
ES: −0.4674
Normalized ES: −1.4498
Signal2Noise
Zero cross at 9095
Rank in ordered gene list
Running enrichment score
Gene ranks
Rank
C
−log10(PValue)
log2FC
Eno1b
Aldob
Adh1
D
ADH1
ALDH3
ENO1
β-tubulin
ADH1/β-tubulin
ALDH3/β-tubulin
ENO1/β-tubulin
E
tR(Lactic acid)=1.49min
tR(Pyruvic acid)=1.48min
tR(Lactic acid)=1.48min
tR(Pyruvic acid)=1.45min
Lactic acid μM/mg protein
Pyruvic acid μM/mg protein
F
ECAR (mpH/min) normalized per cell number
Glucose
Oligomycin
2-DG
Time (minutes)
Max ECAR (mpH/min) normalized per cell number
G
tR(Lactic acid)=1.49min
tR(Pyruvic acid)=1.46min
tR(Pyruvic acid)=1.49min
tR(Pyruvic acid)=1.51min
H
Con
VPA
ns

Figure 2.

Figure 2. Increased glycolysis in ACC neurons of *Shank3*$^{-/-}$ and VPA-treated mice.

A ATP levels in the ACC of WT versus *Shank3*$^{-/-}$ mice, and VPA-treated mice versus their control.
B Glycolytic genes enriched by GESA analysis of transcriptome data of WT and *Shank3*$^{-/-}$ ACC.
C Volcano map of differently expressed genes in the ACC of WT and *Shank3*$^{-/-}$ mice.
D Western blotting of ADH1, ALDH3 and ENO1 in the ACC of WT and *Shank3*$^{-/-}$ mice. Notice the upregulation of ADH1, ALDH3 and ENO1 in *Shank3*$^{-/-}$ ACC.
E LC–MS measurement of lactic acid and pyruvic acid in the ACC of WT and *Shank3*$^{-/-}$ mice. Notice the upregulation of lactic acid and downregulation of pyruvic acid in *Shank3*$^{-/-}$ ACC.
F ECAR assay of primary WT and *Shank3*$^{-/-}$ neurons. Notice the higher ECAR values of *Shank3*$^{-/-}$ neurons upon glucose and oligomycin stimulation.
G LC–MS measurement of lactic acid and pyruvic acid in the ACC of control and VPA-treated mice. Lactic acid is increased in VPA-treated mice.
H ECAR assay of primary cultured control and VPA-treated neurons. Oligomycin stimulated higher ECAR values in VPA-treated neurons.

Data information: $N = 9$ mice in (A), 5 samples from 15 mice in (B, E), 6 samples from 18 mice in (C, G), 3–4 mice in (D), or 5 batches of cells (F, H) per group. Mean ratio ± SEM. The central band, boxes and whiskers of the boxplot in (A) represented median, upper quartile to lower quartile, and Min to Max, respectively. Two-tailed unpaired *t*-test. Welch's *t*-test (Lactic acid analysis in (E). Pyruvic acid analysis in (G) and ATP levels of VPA-treated mice in (A)). $^{*}P < 0.05$. $^{**}P < 0.01$. $^{***}P < 0.001$. WT, wild type. KO, *Shank3*$^{-/-}$. Con, control.
Source data are available online for this figure.

social interaction, but not the social novelty of *Shank3*$^{-/-}$ mice (Fig 4I–K). Additional analysis showed that 2-DG treatment did not affect the anxiety-like and repetitive behaviors of *Shank3*$^{-/-}$ mice (Fig EV3F–H). These data indicated that suppressing glycolysis improves the synaptic and social function of ASD mice.

Axin2 regulates glycolysis via interacting with ENO-1 in ASD neurons

We next explored the relationship between Wnt signaling overactivation and glycolysis in ASD neurons by suppressing Wnt signaling. Primary *Shank3*$^{-/-}$ neurons were treated with XAV939 which inhibits Wnt signaling through stabilizing Axin2, or ICG-001 which inhibits β-catenin/TCF interaction. The ECAR assay showed that XAV939 treatment significantly blocked glycolysis induction by glucose and oligomycin in *Shank3*$^{-/-}$ neurons (Fig 5A). Interestingly, ICG-001, which inhibited glycolysis in hepatocellular carcinoma cells at the same dosage we used (Zuo *et al*, 2021), had no significant effects on glycolysis in *Shank3*$^{-/-}$ neurons (Fig 5B). As the balance between glycolysis and oxidative phosphorylation shifts in neurons according to different energy requirements, we then evaluated the effects of XAV939 and ICG-001 on oxidative phosphorylation. The oxygen consumption rate (OCR) assay showed that XAV939 treatment significantly increased the maximal respiratory capacity in *Shank3*$^{-/-}$ neurons while ICG-001 treatment did not (Fig 5C and D). These data indicated that there might be a cytoplasmic mechanism for stabilized Axin2 in suppressing glycolysis. Previous studies had reported that Axin2 exerts different functions in different intracellular compartments. Mitochondrial accumulation of Axin2 is associated with mitochondrial dysfunction (Mahato *et al*, 2020). Nuclear localization of Axin2 in neural progenitors triggers neuronal differentiation (Fang *et al*, 2013). Considering that glycolytic enzyme ENO1 is upregulated in *Shank3*$^{-/-}$ neurons and that its function also depends on its subcellular localization (Didiasova *et al*, 2019), we hypothesized that if there were any colocalization between Axin2 and ENO1 in ASD neurons. Immunocytochemistry analysis showed punctate immunoreactivity for both Axin2 and ENO1 separated from each other in the cytoplasm of WT neurons (Fig 5E, left panels). However, in *Shank3*$^{-/-}$ neurons, both Axin2 and ENO1 immunoreactivity accumulated in the perinuclear cytoplasm and exhibited colocalization (Fig 5E, middle panels). Upon XAV939 treatment, Axin2 immunoreactivity translocated to the cell membrane and separated from ENO1 immunoreactivity as in WT neurons (Fig 5E, right panels). Western blotting confirmed the membrane accumulation of Axin2 in XAV939-treated *Shank3*$^{-/-}$ neurons (Fig 5F). These data indicated an interaction between Axin2 and XAV939 in *Shank3*$^{-/-}$ neurons. Protein CO-IP revealed basal levels of Axin2/ENO1 interaction in WT

Figure 3. Effects of overexpressing β-catenin in ACC neurons on glycolysis, and synaptic and social function.

A Experimental design and verification of ACC injection.
B Typical images of Golgi staining and reconstructed neurons in the ACC of control (GFP + EX3) and β-catenin overexpressing mice (CaMKII-Cre + EX3).
C, D Spine density and spine subtypes in the ACC of control and β-catenin overexpressing mice. Notice the reduction of total spine density, stubby and mushroom-like spines in β-catenin overexpressing mice.
E, F Sholl analysis of the ACC neurons of control and β-catenin overexpressing mice. There were fewer branching numbers of basal dendrites in β-catenin overexpressing mice.
G, H Patch-clamp recording of mEPSC in the ACC of control and β-catenin overexpressing mice. Both the frequency and average amplitudes of mEPSC were reduced in Wnt overactivated ACC.
I 3-chamber assay of control and β-catenin overexpressing mice.
J, K Resident-intruder assay of control and β-catenin overexpressing mice.
L, M ECAR assay of primary cultured control neurons (GFP + EX3) and β-catenin overexpressing neurons (CaMKII-Cre + EX3).

Data information: Bars = 50 μm in (B) and 20 μm in (C, G). $N = 20$ neurons from 3 mice (B–F), 9 neurons from 3 mice in (G, H), 9–10 mice (I–K) or 5 batches of cells (L, M) per group. Mean ratio ± SEM. Two-tailed unpaired *t*-test (Spine analysis excepting mushroom analysis in (D). Basal dendrites analysis in (E)). Social novelty and social preference scores in (I), (H), (J) and ECAR in (M). Mann–Whitney *U* test (Mushroom analysis in (D). Intersections of apical dendrites in (E)). Two-tailed paired *t*-test (Time in compartment in (I)). Two-way repeated measurement ANOVA test (F). Welch's *t*-test (Length of apical dendrites in (E). Amplitude of mEPSC in (H). Max ECAR in (M)). $^{*}P < 0.05$. $^{**}P < 0.01$. $^{***}P < 0.001$, $^{****}P < 0.0001$.
Source data are available online for this figure.

 © 2023 The Authors

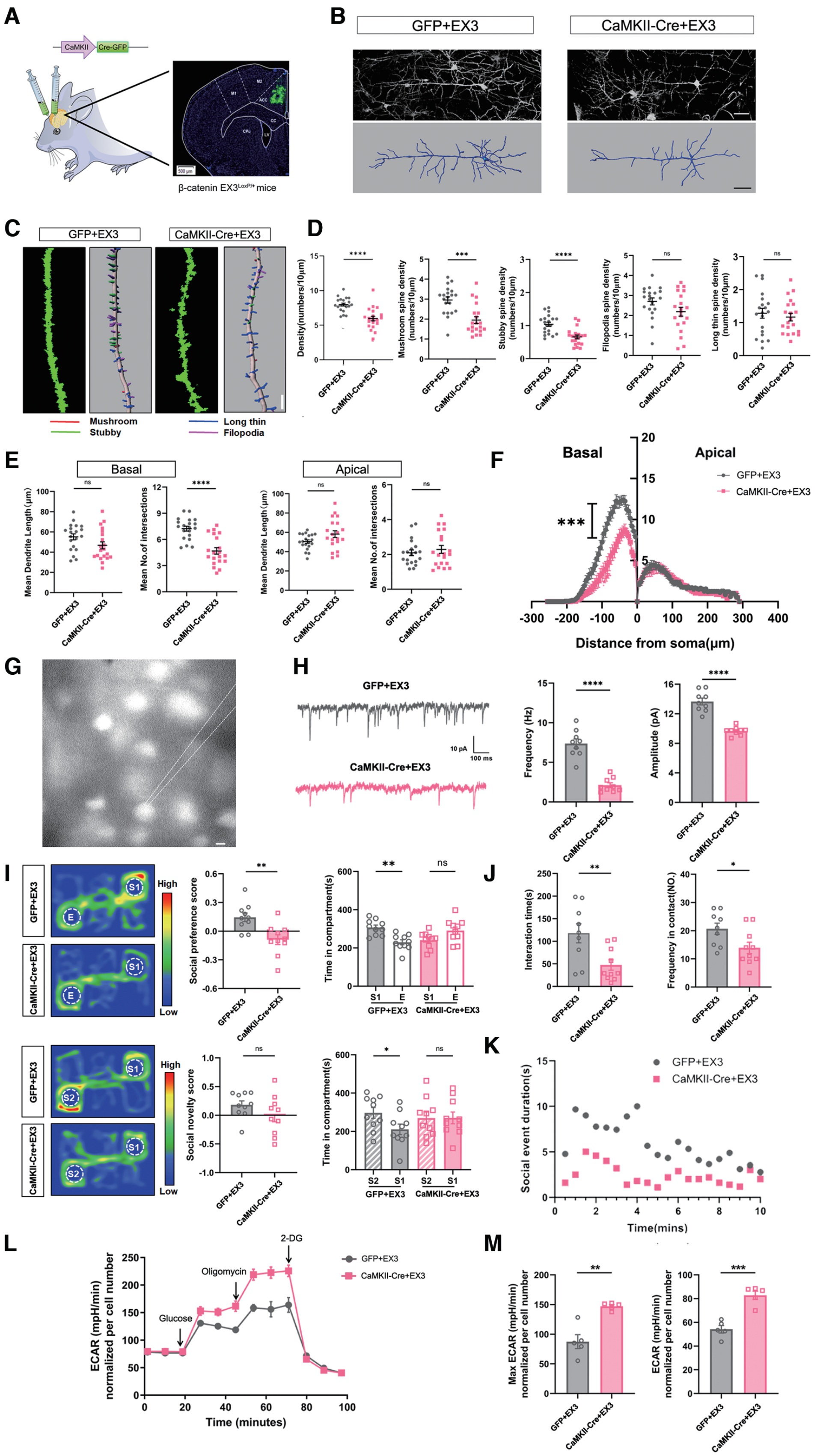
A
CaMKII
Cre-GFP
β-catenin EX3LoxP/+ mice
B
GFP+EX3
CaMKII-Cre+EX3
C
GFP+EX3
CaMKII-Cre+EX3
Mushroom
Stubby
Long thin
Filopodia
D
Density(numbers/10μm)
Mushroom spine density (numbers/10μm)
Stubby spine density (numbers/10μm)
Filopodia spine density (numbers/10μm)
Long thin spine density (numbers/10μm)
E
Basal
Apical
Mean Dendrite Length (μm)
Mean No.of intersections
F
Basal
Apical
GFP+EX3
CaMKII-Cre+EX3
Distance from soma(μm)
G
H
GFP+EX3
CaMKII-Cre+EX3
10 pA
100 ms
Frequency (Hz)
Amplitude (pA)
I
High
Low
Social preference score
Time in compartment(s)
Social novelty score
J
Interaction time(s)
Frequency in contact(NO.)
K
Social event duration(s)
Time(mins)
L
ECAR (mpH/min) normalized per cell number
Glucose
Oligomycin
2-DG
Time (minutes)
M
Max ECAR (mpH/min) normalized per cell number
ECAR (mpH/min) normalized per cell number

Figure 3.

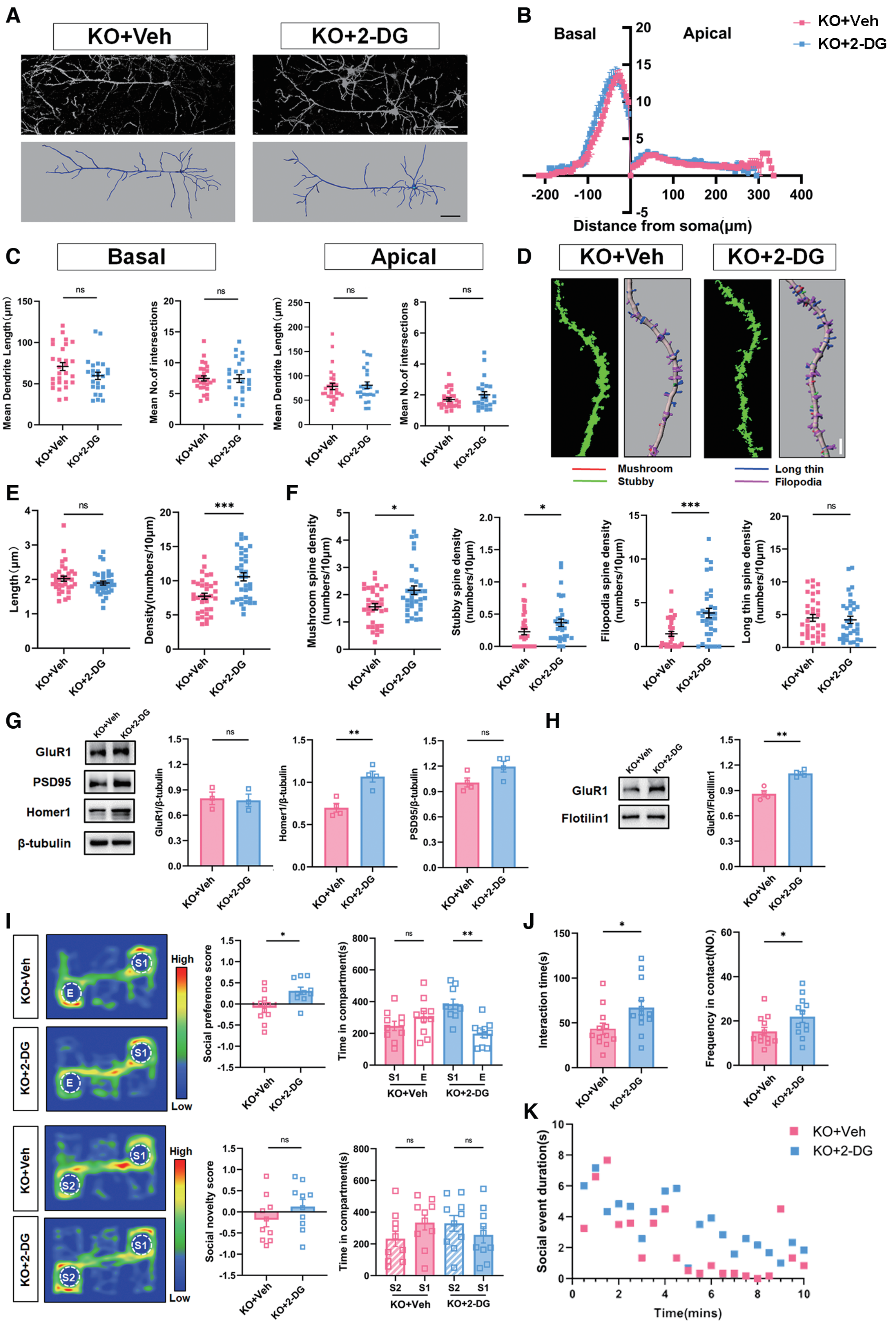

Figure 4.

© 2023 The Authors

Figure 4. Partial restoration of synaptic and social function of *Shank3*$^{-/-}$ mice by 2-DG treatment.

A Typical images of Golgi staining and reconstructed neurons in the ACC of *Shank3*$^{-/-}$ mice treated with 2-DG (KO + 2-DG) or vehicle (KO + Veh).
B, C Sholl analysis of pyramidal neurons in the ACC of *Shank3*$^{-/-}$ mice treated with 2-DG or vehicle.
D–F Spine length, spine density and spine subtypes in the ACC of *Shank3*$^{-/-}$ mice treated with 2-DG or vehicle. Notice the increase of total spine density, stubby, mushroom-like and filapodial spines in 2-DG treated *Shank3*$^{-/-}$ mice.
G Western blotting of GluR1, PSD95 and Homer1 in the total proteins of *Shank3*$^{-/-}$ ACC with or without 2-DG treatment.
H Western blotting of GluR1 in the membrane proteins of *Shank3*$^{-/-}$ ACC with or without 2-DG treatment. Notice the higher levels of GluR1 in membrane proteins of 2-DG treated mice.
I–K 3-chamber assay and resident-intruder assay of *Shank3*$^{-/-}$ mice treated with 2-DG or vehicle. Notice the improvement of social preference and social interaction by 2-DG treatment.

Data information: Bar = 50 μm in (A) and 20 μm in (D). *N* = 24–28 neurons from 4 mice in (A–C), 34 spines from 4 mice in (D–F), 3 samples from 12 mice in (H), 3 mice in (G) or 10–12 mice in (I–K) per group. Mean ratio ± SEM. Two-tailed unpaired *t*-test (Intersection of basal dendrites in (C). Spine density in (E). Social novelty and social preference score in (I, G, H, J)). Mann–Whitney *U* test (Dendrites analysis excepting intersection of basal dendrites in C, Spine length in (E, F)). Two-tailed paired *t*-test (Time in compartment in (I)). Two-way repeated measurement ANOVA test (B). *$P < 0.05$. **$P < 0.01$. ***$P < 0.001$. KO, *Shank3*$^{-/-}$. Veh, vehicle.
Source data are available online for this figure.

neurons. A significantly stronger Axin2/ENO1 interaction was observed in *Shank3*$^{-/-}$ neurons, which was largely abolished by XAV939 treatment (Fig 5G).

In VPA-treated neurons, XAV939 significantly attenuated glycolysis and enhanced oxidative phosphorylation (Figs EV4A and 4B). These data indicated that Axin2 might cross-link Wnt signaling with glycolysis via interacting with ENO1.

Stabilizing Axin2 rescues synaptic and social deficits in ASD mice

Since both Wnt signaling and glycolysis could be effectively suppressed by stabilizing Axin2, we then tested whether XAV939 treatment could rescue the synaptic defects in ASD neurons by continuous administration of XAV939 to the ACC of *Shank3*$^{-/-}$ mice for 3 days (Fig 6A). Sholl analysis showed that XAV939 treatment significantly increased both the branching number and average length of basal dendrites (Fig 6B–D). Further analysis showed that XAV939 significantly enhanced the density of spines, particularly the density of mushroom-like and stubby spines in the ACC of *Shank3*$^{-/-}$ mice (Fig 6E–G). Consistent with the increased mature spines, the average length of spine was much shorter in XAV939-treated *Shank3*$^{-/-}$ ACC (Fig 6G). Western blots showed that XAV939 treatment dramatically upregulated PSD95 and Homer1 expression in *Shank3*$^{-/-}$ ACC (Fig 6H). In terms of glutamate receptors, XAV939 treatment augmented not only the total level of GluR1 but also that of membrane-bound GluR1 in the ACC of *Shank3*$^{-/-}$ (Fig 6I and J).

To explore the effects of XAV939 on neuron function, we first examined the response of cytoplasmic Ca^{2+} of *Shank3*$^{-/-}$ neurons to excitatory stimulation. In comparison with WT neurons, *Shank3*$^{-/-}$ neurons displayed a relatively slow rise and decay of cytoplasmic Ca^{2+} concentrations, and with smaller amplitude, upon KCl stimulation. XAV939 treatment effectively restored both the amplitude and rising time of Ca^{2+} responses of *Shank3*$^{-/-}$ neurons, suggesting a recovery of neuronal electroactivity (Fig 7A–C). Patch-clamp recordings in the ACC of *Shank3*$^{-/-}$ mice showed that XAV939 treatment remarkably enhanced both the frequency and amplitude of mEPSCs (Fig 7D). At the behavioral level, XAV939 treatment remarkably improved the social preference, social novelty, and social interaction of both *Shank3*$^{-/-}$ and VPA-treated mice (Fig 7E–J). More importantly, the social improving effects could last for at least 1 week after the last XAV939 administration (Fig EV4C–E). The grooming and anxiety-like behaviors of both mice were not affected by XAV939 (Fig EV4F–K). These data demonstrated that stabilizing Axin2 effectively rebalanced the glycolysis/oxidative phosphorylation of ASD neurons and could rescue the synaptic deficits and social function upon delivering into ACC.

Abnormal Wnt activation and glycolysis in *Shank3* mutant and VPA-treated human neurons

We next asked whether similar changes occurred in human ASD neurons. To achieve this, we mutated Exon2 of the *Shank3* gene of

Figure 5. Interaction between Axin2 and ENO1 in ASD neurons.

A ECAR assay of primary *Shank3*$^{-/-}$ neurons treated with or without XAV939. Notice the lower levels of glycolysis in XAV939-treated cells.
B ECAR assay of primary *Shank3*$^{-/-}$ neurons treated with or without ICG-001.
C OCR assay of primary *Shank3*$^{-/-}$ neurons treated with or without XAV939. Notice the higher levels of maximal respiratory capacity in XAV939-treated cells.
D OCR assay of primary *Shank3*$^{-/-}$ neurons treated with or without ICG-001.
E Double-immunostaining of ENO1/Axin2 in WT neurons, *Shank3*$^{-/-}$ neurons, and XAV939-treated *Shank3*$^{-/-}$ neurons. Notice the colocalization of Axin2/ENO1 immunoreactivity in *Shank3*$^{-/-}$ neurons and the membrane localization of Axin2 immunoreactivity in XAV939-treated *Shank3*$^{-/-}$ neurons. Arrows point to membrane Axin2 immunoreactivity.
F Western blotting of Axin2 in membrane proteins of WT and *Shank3*$^{-/-}$ neurons treated with or without XAV939. Notice the accumulation of Axin2 in the membrane proteins of XAV939-treated *Shank3*$^{-/-}$ neurons.
G Protein CO-IP of Axin2/ENO1 in WT and *Shank3*$^{-/-}$ neurons treated with or without XAV939. Notice the strong interaction of Axin2/ENO1 in *Shank3*$^{-/-}$ neurons and diminished interaction upon XAV939 treatment.

Data information: Bar = 5 μm in E. *N* = 4–5 batches of cells in (A–D) per group and 3 samples from 9 mice per group in (F, G). Mean ratio ± SEM. Two-tailed unpaired *t*-test (A–D). One-way ANOVA with Tukey's multiple comparison test (F, G). *$P < 0.05$. **$P < 0.01$. WT, wild type. KO, *Shank3*$^{-/-}$.
Source data are available online for this figure.

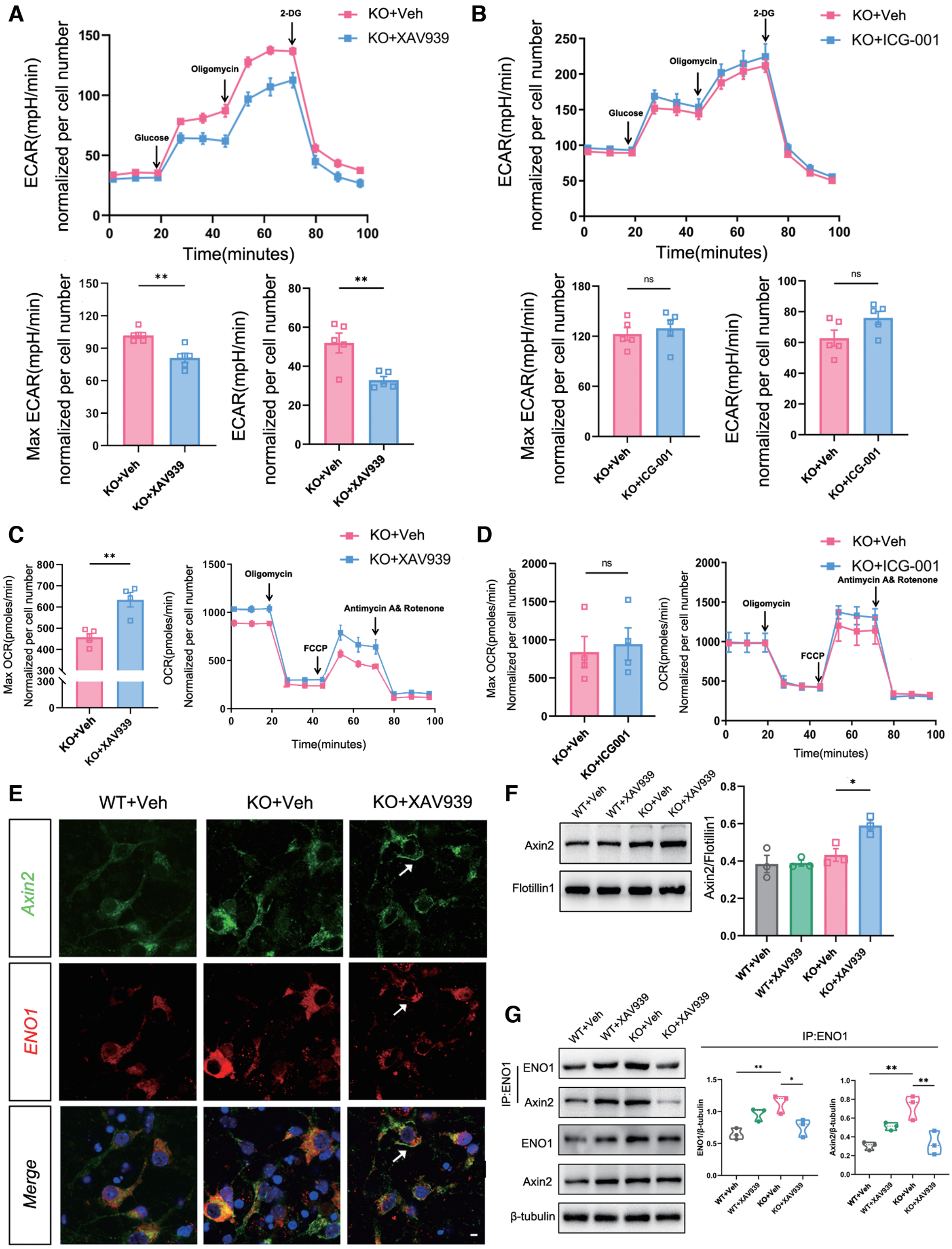

Figure 5.

hESCs (H8 line) using CRISPR/Cas9 (Fig 8A). The *Shank3* mutation was validated by DNA sequencing and Western blotting (Fig EV5A). Control and *Shank3* mutant hESCs were induced to neurons using a well-established protocol, which produced over 80% of Tuj-1-positive cells after 2–3-week induction (Fig EV5B). The expression of key molecules of the Wnt signaling pathway and other synaptic proteins was examined after neural induction. In *Shank3*-mutant human neurons, the levels of p-GSK3β(S9) and TCF1L1 significantly

© 2023 The Authors

increased while those of p-β-catenin (S33) decreased, indicating relatively higher levels of Wnt signaling (Fig 8B). We then evaluated Axin2/ENO1 interaction at different neural differentiation stages. Protein CO-IP assay evidenced similar basal levels of Axin2/ENO1 interaction in both WT and *Shank3*-mutant human neural progenitor cells (NPCs, Fig 8C). Notably, *Shank3*-mutant human neurons displayed much stronger Axin2/ENO1 interaction in comparison with that in WT human neurons (Fig 8D). The ECAR assay showed that *Shank3*-mutant human neurons exhibited significantly higher levels of glycolytic activity, similarly to mouse *Shank3*$^{-/-}$

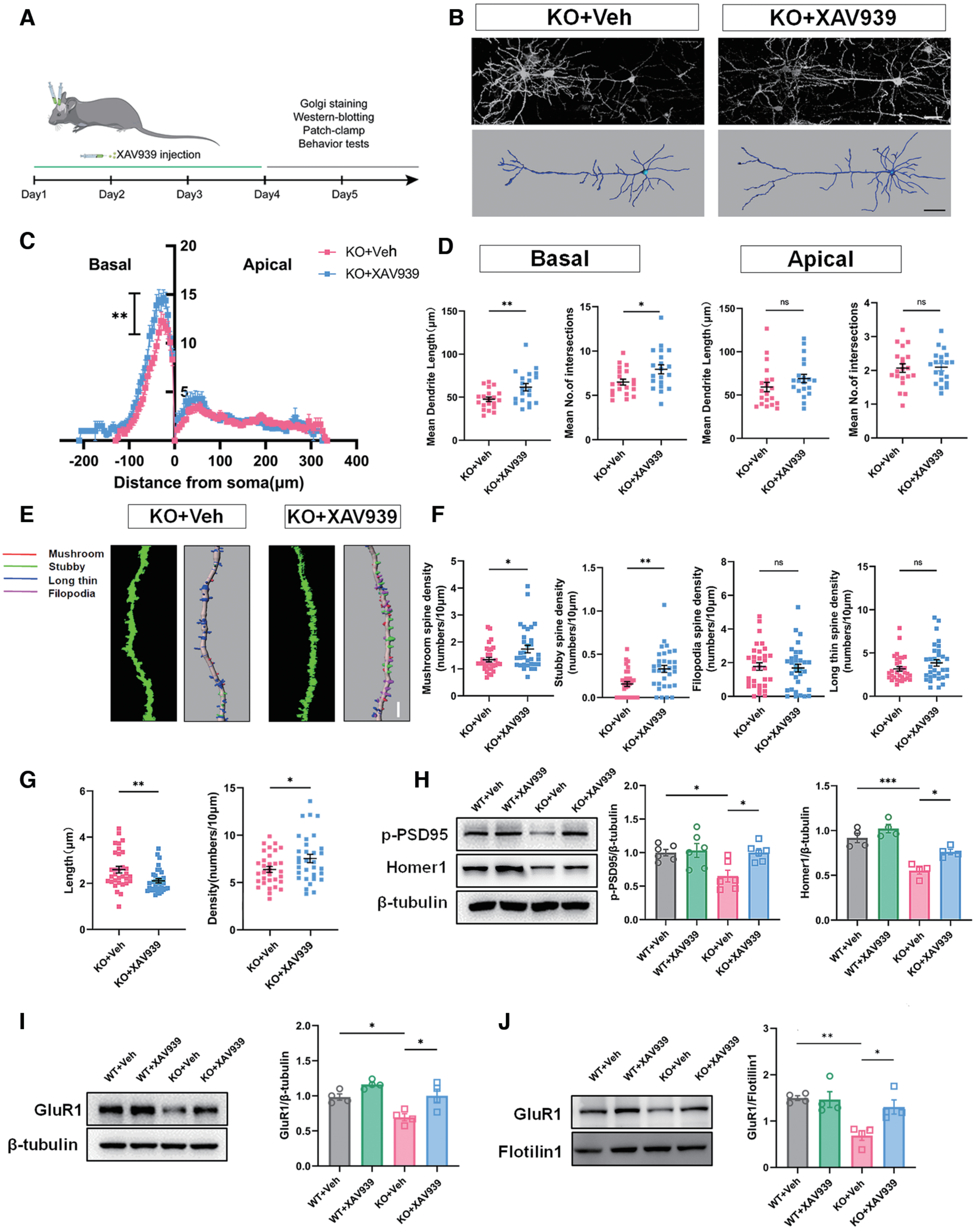

Figure 6.

◀ **Figure 6. Effects of stabilizing Axin2 on the morphology and synaptic protein of *Shank3*$^{-/-}$ ACC neurons.**

A Experimental design.

B Typical images of Golgi staining and reconstructed neurons of *Shank3*$^{-/-}$ ACC with or without XAV939 treatment.

C, D Sholl analysis of ACC neurons in *Shank3*$^{-/-}$ mice treated with or without XAV939. XAV939 treatment significantly increased the branching number and average length of basal dendrites.

E–G Spine length, spine density and spine subtypes of *Shank3*$^{-/-}$ ACC neurons treated with or without XAV939. Notice the increase of total spine density, stubby and mushroom-like spines and the decrease of average spine length in XAV939-treated *Shank3*$^{-/-}$ mice.

H Western blotting of p-PSD95 and Homer1 in WT and *Shank3*$^{-/-}$ ACC treated with or without XAV939.

I, J Western blotting of GluR1 in the total and membrane protein of WT and *Shank3*$^{-/-}$ neurons treated with or without XAV939. Notice the rescuing effects of XAV939 on the expression of GluR1 in *Shank3*$^{-/-}$ neurons.

Data information: Bar = 50 µm in (B) and 20 µm in (E). *N* = 20 neurons from 3 mice in (B–D), 32 spines from 3 mice in (E–G), 4–6 mice in (H, I), and 3 samples from 12 mice in (J) per group. Mean ratio ± SEM. Two-tailed unpaired *t*-test (Intersection of apical and basal dendrites in (D). Filopodia analysis in (F)). Mann–Whitney *U* test (Length of apical dendrites in (D). Spines analysis excepting filopodia analysis in (F). Spine length in (G)). One-way ANOVA with Tukey's multiple comparison test (H–J). Two-way repeated measurement ANOVA test (C). Welch's *t*-test (Length of basal dendrites in (D). Spine density in (G)). $*P < 0.05$, $**P < 0.01$, $***P < 0.001$. WT, wild type. KO, *Shank3*$^{-/-}$.
Source data are available online for this figure.

neurons, which was suppressed by XAV939 (Fig 8E). Further, same XAV939 treatment restored the expression of Homer1, PSD95, and GluR1 in *Shank3*-mutant human neurons (Fig 8F).

We next treated human NPCs with VPA and analyzed the glycolytic activity after 2 weeks of neuronal differentiation (Fig 8G). Remarkable augmented Axin2/ENO1 interaction was also observed in human neurons pretreated by VPA, as compared that with in naïve human neurons (Fig EV5C). In addition, significantly higher glycolysis levels were detected in VPA-treated human neurons (Fig 8H). Similar to *Shank3*-mutant human neurons, XAV939 treatment significantly reduced the reserved and maximal glycolysis of VPA-treated human neurons (Fig 8H). These data indicated that Wnt overactivation and glycolysis were conserved changes in ASD neurons, which can be rescued by stabilizing Axin2.

Discussion

In the present study, by using two ASD mice models (*Shank3*$^{-/-}$, VPA treatment), we first demonstrated aberrant activation of canonical Wnt signaling and aerobic glycolysis in ACC neurons. By artificially activating Wnt signaling, we observed elevation of glycolysis and social dysfunction in WT mice. Blocking glycolysis partially rescued synaptic and social deficits in these ASD models. Interestingly, the interaction between Wnt signaling component Axin2 and glycolytic enzyme ENO1 was robustly augmented in ASD neurons, which could be attenuated by chemical compound XAV939. XAV939 efficiently shifted the weight of energy supply from glycolysis to oxidative phosphorylation, rescued synaptic defects, and improved social function in both ASD models. More importantly, similar Wnt-glycolysis signaling overactivation, Axin2/ENO1 interaction and rescuing effects of stabilizing Axin2 were observed in *Shank3*-mutant and VPA-treated human neurons. In together, these data demonstrated a novel Axin2-coupled excessive Wnt-glycolysis signaling in ASD-associated synaptic and social deficiency.

In general, Wnt signaling is highly active in the embryonic stage, declining postnatally. Most previous studies on Wnt-related ASD risk genes focused on the early stage of neural development (Bernier *et al*, 2014; Durak *et al*, 2015, 2016; Caracci *et al*, 2016; El Khouri *et al*, 2021; Upadhyay *et al*, 2021). Our data demonstrated a relatively high level of canonical Wnt signaling in the ACC neurons of two ASD mice models starting from 2 weeks after birth and persisting to adult, which is in agreement with not only the developmental time course of *Shank3* transcription in cortex (Wang *et al*, 2014) but also the previous observation of decreased GSK3β activity in ACC and upregulation of nuclear β-catenin signaling in the mPFC of *Shank3* mutant mice (Qin *et al*, 2018; Wang *et al*, 2019). The nuclear β-catenin accumulation after disruption of the β-catenin/Shank3 interaction is in consistent with a recent paper

Figure 7. Effects of stabilizing Axin2 on the function of *Shank3*$^{-/-}$ ACC neurons and social behavior of ASD mice. ▶

A–C Calcium response of WT and *Shank3*$^{-/-}$ neurons treated with or without XAV939. Notice that XAV939 treatment significantly restored the amplitude, rising and decay time of calcium response in *Shank3*$^{-/-}$ neurons.

D Patch-clamp recording of mEPSCs in *Shank3*$^{-/-}$ ACC treated with or without XAV939. Both the frequency and amplitude of *Shank3*$^{-/-}$ neurons were increased by XAV939.

E–G 3-chamber and resident-intruder assays of *Shank3*$^{-/-}$ mice treated with or without XAV939. Notice the improvement of social preference, social novelty and social interaction of *Shank3*$^{-/-}$ mice by XAV939 treatment.

H–J 3-chamber and resident-intruder assays of VPA-ASD mice treated with or without XAV939. Notice the improvement of social preference, social novelty and social interaction by XAV939.

Data information: Bar = 15 µm in (A) and 30 µm in magnified images of (A). *N* = 39 neurons from 3 batches of cells in (A–C), 7–8 neurons from 3 mice in (D), or 8–10 mice (E–J) per group. Mean ratio ± SEM. Two-tailed unpaired *t*-test (D). Interaction time in (F). Social preference and social novelty scores in (E) and (H). Mann–Whitney *U* test (I). Two-tailed paired *t*-test (Time in compartment in (E), Time in compartment in VPA-ASD mice treated without XAV939 in (H)). Kruskal–Wallis *H* test with Dunn's multiple comparison test (A–C). Welch's *t*-test (Frequency in contact in (F)). Wilcoxon signed-rank test (Time in compartment in VPA-ASD mice treated with XAV939 in (H)). $*P < 0.05$, $**P < 0.01$, $***P < 0.001$, $****P < 0.0001$. KO, *Shank3*$^{-/-}$. Veh, vehicle.
Source data are available online for this figure.

© 2023 The Authors

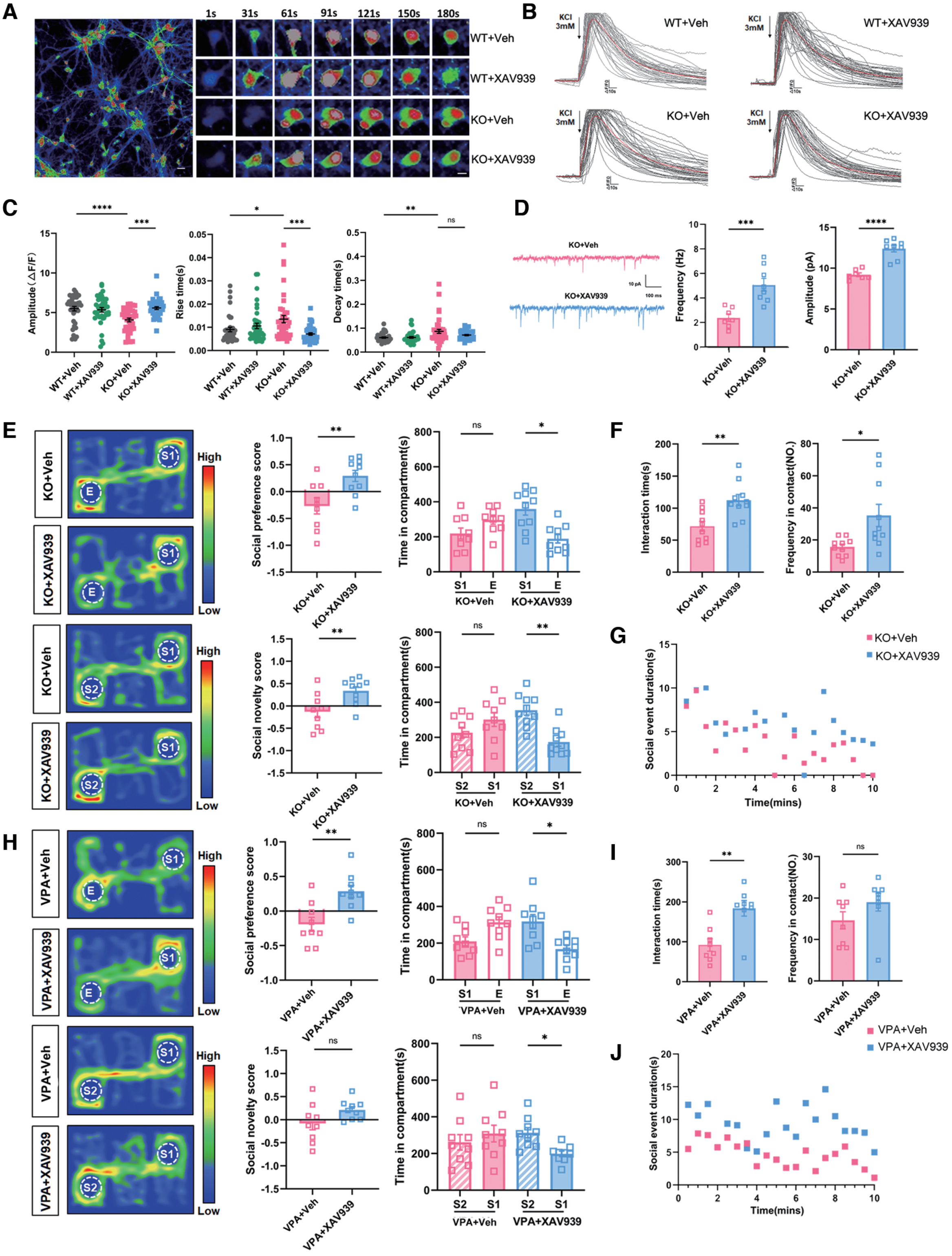

Figure 7.

which reported that a truncated form of Shank3 could shuffle into the nucleus and bind to β-catenin (Hassani Nia *et al*, 2020). It is possible that nuclear Shank3 may competitively inhibit β-catenin/TCF signaling, while cytoplasmic Shank3 confines β-catenin localization to the cytoplasm and membrane. The juvenile-appeared Wnt overactivation implied a postnatal time window for interference, as

© 2023 The Authors

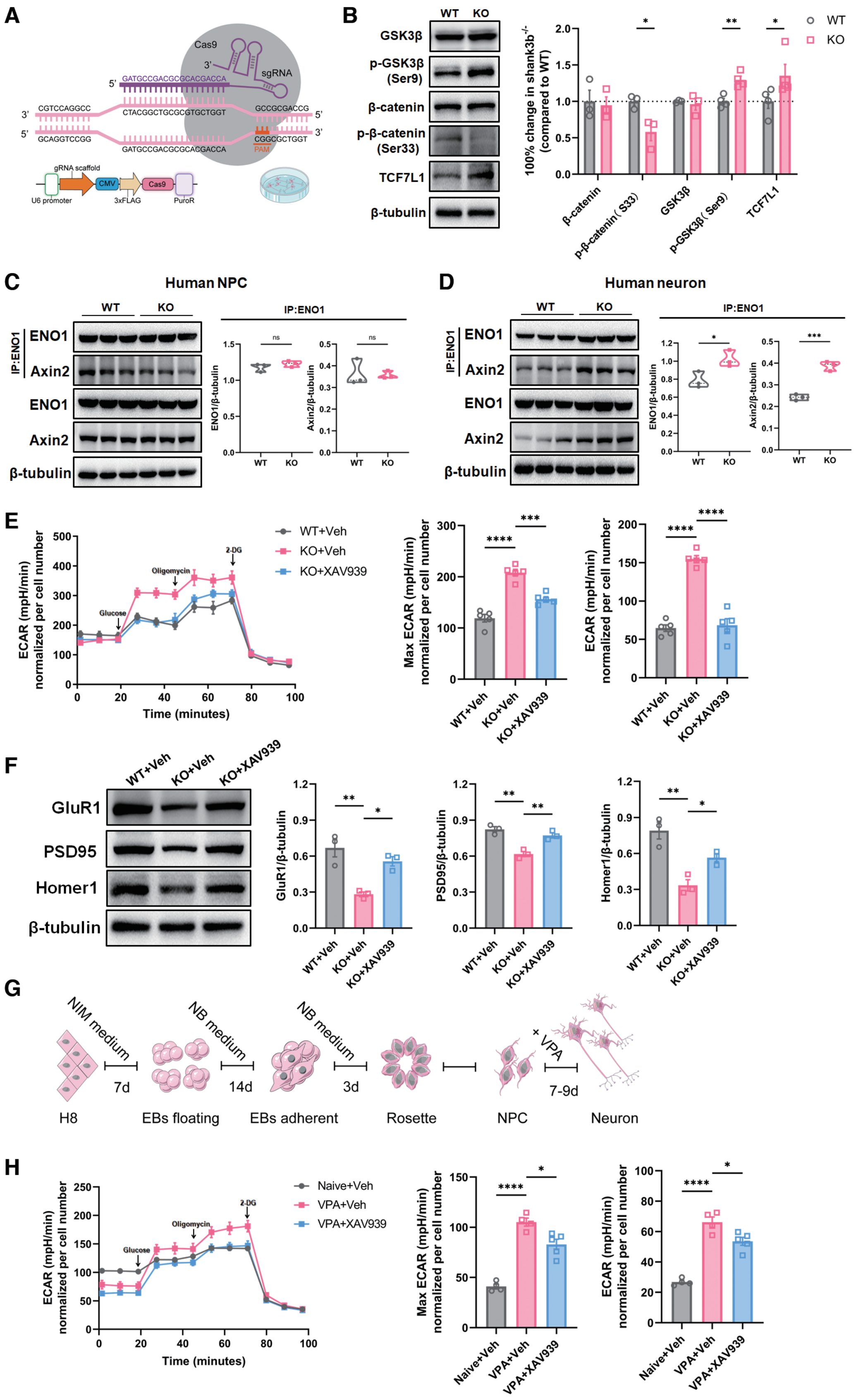

Figure 8.

© 2023 The Authors

Figure 8. Rescuing effects of stabilizing Axin2 on the glycolysis and synaptic deficiency of *Shank3* mutant and VPA-treated human neurons.

A Scheme of CRISPR/CAS9 mutation of human *Shank3*. The tested gRNA sequence is highlighted in purple.
B Western blotting of β-catenin, p-β-catenin(S33), GSK3β, p-GSK3β(S9) and TCF7L1 in neurons derived from WT or *Shank3* mutant human ESCs. Notice the similar change in Wnt signaling components as in *Shank3*$^{-/-}$ mice.
C Protein CO-IP assay of Axin2/ENO1 in WT and *Shank3* mutant human NPCs.
D Protein CO-IP assay of Axin2/ENO1 in WT and *Shank3* mutant human neurons. Notice the strong interaction of Axin2/ENO1 *Shank3* mutant human neurons.
E ECAR assay of WT human neurons, *Shank3* mutant human neurons, and XAV939-treated *Shank3* mutant human neurons. Notice the elevation of glycolysis in *Shank3* mutant human neurons and the suppressive effects of XAV939.
F Western blotting of GluR1, PSD95 and Homer1 in WT human neurons, *Shank3* mutant human neurons, and XAV939-treated *Shank3* mutant human neurons. Notice the reduced expression of GluR1, PSD95 and Homer1 in *Shank3* mutant human neurons and restoration of their expression by XAV939.
G Experimental design of VPA treatment in human NPCs. VPA was given to NPCs to mimic the *in vivo* ASD model induced by VPA exposure.
H ECAR assay of naive human neurons, VPA-pretreated human neurons with or without XAV939 treatment. Notice the higher levels of glycolysis in VPA-pretreated human neurons and the inhibitory effects of XAV939.

Data information: N = 3–4 batches of cells in (B, C, D, F), 4–5 batches of cells in (E, H) per group. Mean ratio ± SEM. Two-tailed unpaired t-test (Wnt components expression excepting TCF7L1 and GSK3β in B, C, D). Mann–Whitney U test (TCF7L1 expression in B). Welch's t-test (GSK3β expression in (B)). One-way ANOVA with Tukey's multiple comparison test (E, F, H). *$P < 0.05$. **$P < 0.01$. ***$P < 0.001$. ****$P < 0.0001$. WT, wild type. KO, *Shank3*$^{-/-}$. Veh, vehicle. EB, embryonic body. NPC, neural progenitor cell.
Source data are available online for this figure.

prenatal inhibition of Wnt signaling may be harmful for organ development.

Valproic acid-induced ASD is widely adopted as an environmentally induced ASD model. A previous study reported rapid Wnt signaling activation in PFC upon VPA treatment (Qin *et al*, 2016). Our data suggested that embryonic exposure to a single dose VPA could produce a long-lasting Wnt signaling activation in ACC, possibly due to the epigenetic effects of VPA.

Our observation that overexpressing stabilized β-catenin in excitatory ACC neurons (CaMKII-positive neurons) induced social deficits is in line with the "excitatory-inhibitory imbalance hypothesis" of ASD (Satterstrom *et al*, 2020), as a previous study reported that deleting β-catenin in inhibitory neurons led to ASD (Dong *et al*, 2016). This implies that postnatal activation of Wnt signaling, for example, by environmental factors, may lead to ASD.

Under normal condition, neurons prefer oxidative phosphorylation rather than glycolysis to meet their high energy requirement for maintaining membrane potential and synaptic transmission (Yellen, 2018). High prevalence of metabolic abnormality, particularly high levels of lactate or lactate-pyruvate ratio in ASD patients, has inspired a "Warburg effect hypothesis" in ASD pathology (Vallee & Vallee, 2018). Our data of ECAR assay and high levels of lactic acid in *Shank3*$^{-/-}$ and VPA-treated neurons clearly demonstrated an increase of glycolysis in ASD neurons. The promotion of membrane-bound GluR1 by 2-DG treatment substantiated a role of sufficient energy supply in synaptic function. The social improving effects of 2-DG we observed, in together with the antiepileptic effects of 2-DG (Stafstrom *et al*, 2009), suggested that inhibiting neuronal glycolysis may be beneficial for ASD treatment, as epilepsy is a concomitant disease of ASD (Tuchman, 2017).

In cancer cells, activated Wnt signaling induces glycolysis by upregulating its downstream target genes, such as PDK1 and PARP (Pate *et al*, 2014; Zuo *et al*, 2021). Surprisingly, inhibiting the function of nuclear β-catenin by ICG001 did not restore the balance of glycolysis/oxidative phosphorylation in ASD neurons while stabilizing Axin2 did. Although the possibility that Wnt signaling regulates glycolysis by its downstream genes in ASD neurons could not be excluded, our data uncovered a direct role of Axin2 in regulating glycolytic activity. The interaction between Axin2 and ENO1 may provide a platform for recruiting glycolytic enzymes, facilitating glycolytic flow. Our data that overexpressing β-catenin in WT ACC stimulated glycolysis is in line with this hypothesis. Being a feedback gene of Wnt signaling, Axin2 is upregulated in neurons overexpressing β-catenin, which potentially augmented Axin2/ENO1 interaction and elevated glycolysis. More interestingly, treating ASD neurons with XAV939, a stabilizer of Axin2, effectively anchored Axin2 on cell membrane, thereby preventing the interaction of Axin2/ENO1 in cytoplasm and suppressing glycolysis. In addition, the membrane-bound Axin2 would promote the degradation of β-catenin and exerted its function in suppressing canonical Wnt signaling.

Previous studies have proposed Wnt signaling as a potential target for ASD treatment, mainly relying on the gene-regulation effects of Wnt signaling (Bae & Hong, 2018). The direct regulation of glycolysis by Axin2 suggested a rapid effect of Wnt signaling on synaptic function. Our observation that stabilizing Axin2 efficiently restored glycolysis/oxidative phosphorylation balance, promoted synaptic maturation, and improved multiple aspects of social function in both *Shank3*$^{-/-}$ and VPA-treated mice confirmed the role of this Wnt signaling-glycolysis cascade in ASD social dysfunction. A weak point of the compound of XAV939 is that it cannot be applied systematically. Owning to the technique difficulties of local delivering it into ACC at early stage, we made interference mainly on young adult mice in the present study. Detailed pharmacological studies focusing on developmental stage are worthy to be conducted in the future. Nevertheless, our data suggested that adult inhibiting Wnt-glycolysis signaling is effective for ASD treatment.

More importantly, we detected similar Wnt signaling activation and aerobic glycolysis in *Shank3* mutant and VPA-treated human neurons. The enhanced Axin2/ENO1 interaction in *Shank3* mutant and VPA-treated human neurons but not in human NPCs was consistent with the postnatal appearance of excessive Wnt-glycolysis signaling in ASD mouse models. Although the detailed mechanisms underlying the Wnt activation and glycolysis in *Shank3* mutant and VPA-induced ASD models may be different, our data indicated that neuronal Wnt signaling-glycolysis cascade may be a conserved mechanism underlying ASD synaptic dysfunction, and Axin2 may be a potential convergent therapeutic target for improving social function.

© 2023 The Authors

Materials and Methods

Mice and treatment

Mice

Wild-type (WT) C57 mice were obtained from the animal facility of the Fourth Military Medical University. *Shank3*$^{-/-}$ mice were a kind gift from Prof. Guoping Feng as described (Peca *et al*, 2011). CaMKII-Cre and β-cateninEX3$^{loxp+/-}$ mice were obtained from the Jackson laboratory. VPA-treated mice were obtained by breeding up progenies of pregnant mice which were exposed to VPA at E12.5 (i.p. injection, 500 mg/kg). Mice were maintained at ambient temperature with a 12 h dark-and-light cycle and given free access to water and food. All animal procedures were approved by the Committee of the Animal Care and Use Committee of Fourth Military Medical University and performed according to ARRIVE guidelines. Most of the experiments, except for those specifically mentioned, were performed on mice of 4–6 weeks old. All mice for the same experimental group were randomly allocated treatment of experimental procedure.

Virus injection

Mice were anesthetized with isoflurane. The adeno-associated virus (AAV) expressing CaMKII-Cre (1×10^{13} PFU) was obtained from Brain VTA (Wuhan, China). The AAV expressing Axin2 shRNA was bought from Obio Biotech (Shanghai, China). The virus (200 nL/injection site) was injected into the bilateral anterior cingulate cortex (ACC) at 0.23 mm left or right to bregma and 0.93 mm anterior to bregma at 1.6 mm in depth. An AAV expressing green fluorescent protein (GFP) was adopted as control.

XAV939 administration

Mice were anesthetized as above. Four days before drug administration, microinjection guide cannulas (26 ga) were secured to the skull for drug delivery. After habituation, microinjection was performed for three consecutive days, through microinjection needles mounted on a micro infusion pump (RWD Life Science). Flow rate was set at 200 nl/min. 500 nl/side of XAV939 (MCE, HY-15147, 20 μM) was given. Vehicle solution (0.1% DMSO in 0.9% NaCl) was used as control. After injection, the needles were kept in place for one more minute for drug diffusion.

2-Deoxy-d-glucose treatment

2-Deoxy-d-glucose (2-DG) was administered to *Shank3*$^{-/-}$ mice via i.p. injection at a dose of 2.5 mg/10 g for five consecutive days. At 24 h after the last 2-DG administration, social function and synaptic development were tested in the injected mice.

Primary neuron culture and treatment

Anterior cingulate cortices of E18-P0 wild-type (WT), *Shank3*$^{-/-}$ and CaMKII-Cre:β-cateninEX3$^{loxp+/-}$ mice were removed in sterile. After digestion, isolated neurons were seeded in neurobasal medium supplemented with 1% N_2 and 2% B27 for 5–6 days.

To interfere Shank3/β-catenin interactions, anti-Shank3 antibody (20 ng/μl, Cat. 64555, Cell Signaling) was added to the culture medium at 5 days *in vitro* (DIV). Anti-IgG (1 μg/100 μl) antibody was added as control. An antibody and peptide transfection reagent (Cat: 501-04, Polyplus transfection) was used to assist the cellular entrance of these antibodies.

XAV939 treatment

XAV939 was added at 5 DIV and maintained at 20 μM for 3 days; cells were analyzed at 5 days after XAV939 treatment.

Protein co-immunoprecipitation

Anterior cingulate cortices were dissected and lysed in immunoprecipitation buffer with a cocktail containing proteinase inhibitors. After centrifugation, supernatant was immunoprecipitated by protein A/G PLUS-Agarose Immunoprecipitation Reagent according to the manufacturer's instructions. Briefly, lysates were first incubated with 20 μl protein A/G PLUS-Agarose or 1 μg control IgG. After bead separation, Anti-Shank3, anti-β-catenin, or anti-ENO1 antibody (2 μg) was added to the supernatant and incubated overnight. Subsequently, 20 μl protein A/G PLUS-Agarose was added for another 4 h. Then, the immunoprecipitates were processed by immunoblotting with the appropriate antibodies (anti-β-catenin, anti-Axin2, or anti-Shank3).

Golgi staining and spine analysis

Golgi staining was conducted as described with minor modification. The brain was dissected and fixed in Golgi-Cox solution (consisting a mixture of potassium dichromate, potassium chromate, and mercuric chloride at a concentration of 5%) in darkness for 14–16 days. Next, brains were dehydrated by 25–30% sucrose before making frozen sections (100–200 μm). For staining, sections were placed in 50% NH_4OH, fixing solution, and subsequently 5% sodium thiosulfate. Then, dehydration in gradient ethanol was carried out. The images were obtained and analyzed by a researcher who was blind to experimental design.

Behavioral tests

Three-Chamber test

The 3-chamber apparatus was made by an opaque acrylic box (65 × 45 × 25 cm) with three chambers (43 × 23 cm). After habituation, a stimulus mouse was placed in the cylinder in the "social chamber," while the cylinder in the "non-social chamber" kept empty. The time the test mice spent in the social versus nonsocial chambers was measured. The behavior of each mouse was video-recorded for assessing the details of social behavior. Each chamber was cleaned with 75% ethanol between tests. The behavior was analyzed using SMART3·0 software (Panlab Harvard Apparatus, Spain). The (Time social − Time non-social)/(Time social + Time non-social) was adopted as preference score.

Resident-juvenile-intruder test

The test was performed as previously described with minor modification. Briefly, the resident mouse (test mouse) was allowed to explore freely in his home cage. The intruder mouse (novel, 3–4 weeks old) was introduced in the cage. The test mouse was allowed to explore the intruder mouse freely for 10 min. Juvenile intruder was used to avoid mutual aggression. The intruder mouse was changed in each test. The time and frequency of direct contacts were measured.

© 2023 The Authors

CRISPR/CAS9 mediated *Shank3* mutation in human embryonic stem cells

Guide RNA sequences corresponding to human Shank3 (gRNA1: GATGCCGACGCGCACGACCA; gRNA2: ATGCAGCTGAGCCGCGCC GC; gRNA3: AGCGCCGTGGTCGTGCGCGT) were cloned into PX459v2·0. After sequence confirmed, constructs were transfected into H8 human ESCs. Transfected cells were sorted, and single cells were seeded onto 96-well plates. Each single-cell colony was cultured to confluence, reseeded, and collected for DNA sequencing. gRNA1 showed the best efficiency of *Shank3* mutation (Fig EV4A). The sequencing primers are as follows: Forward primer: CGCTTCCC TCCCGTCTCAG. Reverse primer: TCCAGGCGCAGGCACTTCT.

Human embryonic stem cell culture and VPA treatment

The human embryonic stem cell (hESC) line H8 (RRID: CVCL_9389) was obtained from Prof. Wei Jiang (Wuhan University). Cells were maintained in mTeSR1 medium (Cat. 85850, Stem Cells) with feeder cell free. Neuron induction was conducted according to the reported protocol with minor modification. Briefly, hESCs were digested using accutase (Cat. 07920, Stem Cells) and cultured in suspension using a neural induction medium (NIM, Cat.08582, Stem Cells) till embryonic body (EB) formation. EBs were transferred to 6-well plates until appearance of rosettes. Rosettes were reseeded and cultured with neurobasal medium containing 2% B27, 100 nmol/L brain-derived neurotrophic factor (Cat. 450-02, PeproTech) and 100 nmol/L glial-derived neurotrophic factor (Cat. 450-10, PeproTech) for neuronal differentiation. VPA (1 mM, P4543, Sigma) was added at 3 days after rosette formation.

Extracellular acidification rate (ECAR) and oxygen consumption rate (OCR)

The assays were conducted using a Seahorse XF-24 extracellular flux analyzer (Seahorse Bioscience). Cells were seeded at a density of 8×10^5 cells per well. One day before measurement, the sensor cartridge was calibrated overnight. OCR or ECAR was measured sequentially before or after the addition of the following reagents: oligomycin (1 μM), p-trifluoremethoxyphenylhydrazone (FCCP, 1 μM), antimycin A (1 μM), rotenone (1 μM), glucose (10 mM) and 2-DG (50 mM). Each measurement cycle included the following steps: 2 min mixture, 1 min waiting, and 3 min measurement. Following the last assay cycle, the plate was removed and cell counting was conducted. Total cell data were used to normalize the measured ECAR and OCR values.

In vitro electrophysiology

The mice were anesthetized as described above. Coronal slices (250 μm-thick) containing the ACC were prepared using a vibratome (VT1200S, Leica). Whole-cell patch-clamp recordings were performed using a multiclamp 700B amplifier (Molecular Devices) under infrared differential interference contrast visualization. Clampex 10.7 was used for data acquisition. For miniature excitatory postsynaptic currents (mEPSCs) recording, the cell membrane potential was clamped at −70 mV and the data were analyzed using Mini Analysis (Synaptosoft Inc.).

Calcium imaging

For detecting calcium responses, neurons were incubated with media containing 4 μM Fluo-8AM (ab142773, Abcam, CA, USA) for 15 min. After resting Ca^{2+} levels were recorded, 3 mM KCl was added. Fluorescent signals were excited at 488 nm and imaged every 1 s for 180 s using a confocal microscope (Olympus, FV3000, Japan). Calcium influx and resting Ca^{2+} levels were measured by image analysis software Cellcens (Olympus, Japan). For each experimental condition, the ΔF/F value of more than 100 cells was calculated using Igor Pro software (WaveMetrics, Oregon, USA). The results from ≥ 3 independent experiments were averaged.

Protein CO-IP MS and transcriptome analysis

For protein CO-IP MS, the ACC protein (approximately 500 μg total protein) was incubated with 2 μg primary anti-Shank3 antibody or IgG overnight and then with 20 μl Protein A/G PLUS-Agarose for 4 h. Immunoprecipitates were collected and separated by SDS–PAGE. The gel lysis and protein digestion were performed by using commercially available iST Sample Preparation kit (PreOmics, Germany). The peptides were analyzed by nano-HPLC–MS/MS, and the resulting data were processed by PEAKS Studio version 10.6 (Bioinformatics Solutions Inc., Waterloo, Canada) by Gene Denovo Biotechnology Co. (Guangzhou, China).

For transcriptome analysis, total RNA of WT and *Shank3*$^{-/-}$ ACC was extracted using Trizol reagent kit (Invitrogen, Carlsbad, CA, USA) according to the manufacturer's protocol. RNA quality assessment, reverse transcription, PCR amplification and sequencing, and data analysis were performed by Gene Denovo Biotechnology Co. (Guangzhou, China).

Measurement of ATP, lactic acid, and pyruvic acid

For ATP measurement, the ACC tissue was carefully dissected and immediately homogenized. ATP levels were monitored using a commercial kit (BC0300, Solarbio).

For measuring the levels of lactic acid and pyruvic acid in adult mice, chromatographic separation was performed with a liquid chromatography–mass spectrometer (LC–MS) from Thermo Fisher. The assay was conducted using an amide column (2.1 × 10 mm, 1.7 μm particle size) under isocratic elution with 95% acetonitrile, 10 mM ammonium acetate, and 0.04% ammonia with the flow rate of 0.2 ml/min.

For the levels of lactic acid and pyruvic acid at developmental time points (E16.5, P7, P14, P21, and P28), chromatographic separation was performed using liquid chromatography with tandem mass spectrometry (API 4000 LC/MS/MS). The assay was conducted using an Allure PFP Propyl Column (100 mm × 2.1 mm, 5 μm particle size), with the flow rate of 0.15 ml/min. Concentrations were calculated using internal calibration.

Western blotting and immunocytochemistry

For western blotting, ACC tissues were lysed with RIPA buffer containing a proteinase inhibitor cocktail. Membrane and nuclear proteins were extracted according to the membrane/nuclear protein extraction kit instructions (MinuteTM, Invent Biotechnologies, Inc.).

Proteins were separated by SDS–PAGE and processed with regular protocols. After primary antibody incubation, membranes were incubated with corresponding HRP-conjugated secondary antibodies. Bands were visualized by an ECL kit (Thermo). Images were acquired by a Tanon imaging system and analyzed by ImageJ. For blot quantification, the (gray scale of target protein)/(gray scale of β-tubulin) ratio in experimental groups was compared to that of control groups.

For immunocytochemistry, cells were fixed with 4% cold paraformaldehyde phosphate buffer and penetrated with PBS containing 0.1% Triton X-100 for 10–15 min. After 24 h primary antibody incubation at 4°C, cells were incubated with secondary antibodies conjugated with Alexa Fluor 594 or Alexa Fluor 488 (Jackson ImmunoResearch). All the images were taken under a confocal microscope (FV3000, Olympus).

All the primary and secondary antibodies used in western blots and immunocytochemistry are included in Table EV1.

Statistical analysis

All the morphological and behavior analysis was made by experienced researchers who were blind to the experimental design. Data were expressed as mean ratio ± SEM and analyzed by using GraphPad Prism 8.0 and SPSS 21.0 software. The homogeneity of variance test was assessed with Levene's test and the normality of the Shapiro–Wilk test.

For the data which met the normality and homogeneity of variance: Two-tailed unpaired *t*-test, one-way ANOVA with Tukey's multiple comparisons test, two-tailed paired *t*-test, and two-way repeated measurement ANOVA were adopted. For those not: Mann–Whitney *U* test or Kruskal–Wallis *H* test with Dunn's multiple comparisons test, Welch's *t*-test, or Wilcoxon signed-rank test was conducted. Statistical significance was assessed at levels of $P < 0.05$. The statistical methods for each figure were included in Dataset EV1.

Data availability

All data are available in the main text or the supplementary materials. This study includes no data deposited in external repositories.

Expanded View for this article is available online.

Acknowledgements

This work was funded by project from the Ministry of Science and Technology of China (No. 2021ZD0201005) to Dr. Shengxi Wu, National Natural Science Foundation of China (No. 82221001, No. 81730035) to Dr. Shengxi Wu and project of Shaanxi key R & D program (Grant No. 2022ZDLSF01-02) to Dr. Yazhou Wang. The authors thank Dr. Haoting Li (458 hospital of PLA), Mao Sun and Yuqian Li (Fourth Military Medical University) for their technical help. The authors thank Dr. Guoping Feng (MIT McGovern Institute) for kind gift of *Shank3*$^{-/-}$ mice and Dr. Wei Jiang (Wuhan University) for kind gift of human embryonic stem cell line.

Author contributions

Mengmeng Wang: Conceptualization; data curation; validation; investigation; visualization; methodology; writing – review and editing. **Panpan Xian:** Investigation; methodology. **Weian Zheng:** Investigation; methodology. **Zhenzhen Li:** Investigation; methodology. **Andi Chen:** Investigation. **Haoxiang Xiao:** Investigation. **Chao Xu:** Investigation. **Fei Wang:** Investigation. **Honghui Mao:** Data curation; methodology. **Han Meng:** Investigation. **Youyi Zhao:** Investigation. **Ceng Luo:** Supervision; methodology. **Yazhou Wang:** Conceptualization; supervision; funding acquisition; validation; writing – original draft; project administration; writing – review and editing. **Shengxi Wu:** Conceptualization; resources; supervision; funding acquisition; validation; writing – original draft; project administration; writing – review and editing.

The paper explained

Problem

As more and more risk genes and environmental factors associated with ASD development are identified, it becomes increasingly important to investigate the convergent molecular mechanism for ASD social dysfunction. Genetic studies and network analysis have suggested Wnt signaling as one of the major signaling pathways in the development of ASD. Clinical observation has reported metabolic abnormalities in over 30% of ASD patients. However, whether Wnt-glycolysis signaling and their cross-talk contribute to ASD synaptic defects and social dysfunction is still an open question in the field. Our study addressed this question in detail by using two ASD mouse models and their corresponding human neurons.

Results

Our data revealed overactivation of Wnt signaling and glycolysis in the anterior cingulate cortex (ACC, a key brain region for social function) of *Shank3*$^{-/-}$ and VPA-treated ASD mice from a young age, as well as in corresponding human neurons. Overexpressing β-catenin in ACC is sufficient to induce glycolysis and social dysfunction. Inhibiting Wnt signaling and glycolysis could rescue the ASD-associated synaptic and social phenotype. Further, we demonstrated Axin2/ENO1 interaction as a convergent point of Wnt-glycolysis signaling in both mouse and human ASD neurons.

Impact

Our findings demonstrate that Wnt-glycolysis is aberrantly increased in ASD neurons from juveniles and XAV939 can disrupt Axin2/ENO1 interaction, inhibit Wnt-glycolysis signaling, and restore synaptic and social function. These findings are important for clinical translation, as they suggest a therapeutic time window and a potential compound for treating ASD social dysfunction.

Disclosure and competing interests statement

The authors declare that they have no conflict of interest.

References

Anagnostou E (2018) Clinical trials in autism spectrum disorder: evidence, challenges and future directions. *Curr Opin Neurol* 31: 119 – 125

Antoine MW, Langberg T, Schnepel P, Feldman DE (2019) Increased excitation-inhibition ratio stabilizes synapse and circuit excitability in four autism mouse models. *Neuron* 101: 648 – 661

Bae SM, Hong JY (2018) The Wnt signaling pathway and related therapeutic drugs in autism spectrum disorder. *Clin Psychopharmacol Neurosci* 16: 129 – 135

 © 2023 The Authors

Bernier R, Golzio C, Xiong B, Stessman HA, Coe BP, Penn O, Witherspoon K, Gerdts J, Baker C, Vulto-van Silfhout AT *et al* (2014) Disruptive CHD8 mutations define a subtype of autism early in development. *Cell* 158: 263 – 276

Bonvento G, Bolanos JP (2021) Astrocyte-neuron metabolic cooperation shapes brain activity. *Cell Metab* 33: 1546 – 1564

Caracci MO, Avila ME, De Ferrari GV (2016) Synaptic Wnt/GSK3beta signaling hub in autism. *Neural Plast* 2016: 9603751

Devine MJ, Kittler JT (2018) Mitochondria at the neuronal presynapse in health and disease. *Nat Rev Neurosci* 19: 63 – 80

Didiasova M, Schaefer L, Wygrecka M (2019) When place matters: shuttling of Enolase-1 across cellular compartments. *Front Cell Dev Biol* 7: 61

Dong F, Jiang J, McSweeney C, Zou D, Liu L, Mao Y (2016) Deletion of CTNNB1 in inhibitory circuitry contributes to autism-associated behavioral defects. *Hum Mol Genet* 25: 2738 – 2751

Durak O, de Anda FC, Singh KK, Leussis MP, Petryshen TL, Sklar P, Tsai LH (2015) Ankyrin-G regulates neurogenesis and Wnt signaling by altering the subcellular localization of beta-catenin. *Mol Psychiatry* 20: 388 – 397

Durak O, Gao F, Kaeser-Woo YJ, Rueda R, Martorell AJ, Nott A, Liu CY, Watson LA, Tsai LH (2016) Chd8 mediates cortical neurogenesis via transcriptional regulation of cell cycle and Wnt signaling. *Nat Neurosci* 19: 1477 – 1488

El Fotoh W, El Naby SAA, Abd El Hady NMS (2019) Autism spectrum disorders: the association with inherited metabolic disorders and some trace elements. A retrospective study. *CNS Neurol Disord Drug Targets* 18: 413 – 420

El Khouri E, Ghoumid J, Haye D, Giuliano F, Drevillon L, Briand-Suleau A, De La Grange P, Nau V, Gaillon T, Bienvenu T *et al* (2021) Wnt/beta-catenin pathway and cell adhesion deregulation in CSDE1-related intellectual disability and autism spectrum disorders. *Mol Psychiatry* 26: 3572 – 3585

Fang WQ, Chen WW, Fu AK, Ip NY (2013) Axin directs the amplification and differentiation of intermediate progenitors in the developing cerebral cortex. *Neuron* 79: 665 – 679

Frye RE (2020) Mitochondrial dysfunction in autism Spectrum disorder: unique abnormalities and targeted treatments. *Semin Pediatr Neurol* 35: 100829

Gazestani VH, Pramparo T, Nalabolu S, Kellman BP, Murray S, Lopez L, Pierce K, Courchesne E, Lewis NE (2019) A perturbed gene network containing PI3K-AKT, RAS-ERK and WNT-beta-catenin pathways in leukocytes is linked to ASD genetics and symptom severity. *Nat Neurosci* 22: 1624 – 1634

Guo B, Chen J, Chen Q, Ren K, Feng D, Mao H, Yao H, Yang J, Liu H, Liu Y *et al* (2019) Anterior cingulate cortex dysfunction underlies social deficits in Shank3 mutant mice. *Nat Neurosci* 22: 1223 – 1234

Hassani Nia F, Woike D, Kloth K, Kortum F, Kreienkamp HJ (2020) Truncating mutations in SHANK3 associated with global developmental delay interfere with nuclear beta-catenin signaling. *J Neurochem* 155: 250 – 263

Herbert MR (2010) Contributions of the environment and environmentally vulnerable physiology to autism spectrum disorders. *Curr Opin Neurol* 23: 103 – 110

Iakoucheva LM, Muotri AR, Sebat J (2019) Getting to the cores of autism. *Cell* 178: 1287 – 1298

Kalkman HO (2012) A review of the evidence for the canonical Wnt pathway in autism spectrum disorders. *Mol Autism* 3: 10

Li Q, Liang J, Fu N, Han Y, Qin J (2021) A ketogenic diet and the treatment of autism spectrum disorder. *Front Pediatr* 9: 650624

Mahato B, Kaya KD, Fan Y, Sumien N, Shetty RA, Zhang W, Davis D, Mock T, Batabyal S, Ni A *et al* (2020) Pharmacologic fibroblast reprogramming into photoreceptors restores vision. *Nature* 581: 83 – 88

Mu C, Corley MJ, Lee RWY, Wong M, Pang A, Arakaki G, Miyamoto R, Rho JM, Mickiewicz B, Dowlatabadi R *et al* (2020) Metabolic framework for the improvement of autism spectrum disorders by a modified ketogenic diet: a pilot study. *J Proteome Res* 19: 382 – 390

Newschaffer CJ, Croen LA, Daniels J, Giarelli E, Grether JK, Levy SE, Mandell DS, Miller LA, Pinto-Martin J, Reaven J *et al* (2007) The epidemiology of autism spectrum disorders. *Annu Rev Public Health* 28: 235 – 258

Noelanders R, Vleminckx K (2017) How Wnt signaling builds the brain: bridging development and disease. *Neuroscientist* 23: 314 – 329

Pate KT, Stringari C, Sprowl-Tanio S, Wang K, TeSlaa T, Hoverter NP, McQuade MM, Garner C, Digman MA, Teitell MA *et al* (2014) Wnt signaling directs a metabolic program of glycolysis and angiogenesis in colon cancer. *EMBO J* 33: 1454 – 1473

Peca J, Feliciano C, Ting JT, Wang W, Wells MF, Venkatraman TN, Lascola CD, Fu Z, Feng G (2011) Shank3 mutant mice display autistic-like behaviours and striatal dysfunction. *Nature* 472: 437 – 442

Qin L, Dai X, Yin Y (2016) Valproic acid exposure sequentially activates Wnt and mTOR pathways in rats. *Mol Cell Neurosci* 75: 27 – 35

Qin L, Ma K, Wang ZJ, Hu Z, Matas E, Wei J, Yan Z (2018) Social deficits in Shank3-deficient mouse models of autism are rescued by histone deacetylase (HDAC) inhibition. *Nat Neurosci* 21: 564 – 575

Rossignol DA, Frye RE (2012) Mitochondrial dysfunction in autism spectrum disorders: a systematic review and meta-analysis. *Mol Psychiatry* 17: 290 – 314

Sanders SJ, Murtha MT, Gupta AR, Murdoch JD, Raubeson MJ, Willsey AJ, Ercan-Sencicek AG, DiLullo NM, Parikshak NN, Stein JL *et al* (2012) De novo mutations revealed by whole-exome sequencing are strongly associated with autism. *Nature* 485: 237 – 241

Satterstrom FK, Kosmicki JA, Wang J, Breen MS, De Rubeis S, An JY, Peng M, Collins R, Grove J, Klei L *et al* (2020) Large-scale exome sequencing study implicates both developmental and functional changes in the neurobiology of autism. *Cell* 180: 568 – 584

Sohal VS, Rubenstein JLR (2019) Excitation-inhibition balance as a framework for investigating mechanisms in neuropsychiatric disorders. *Mol Psychiatry* 24: 1248 – 1257

Stafstrom CE, Ockuly JC, Murphree L, Valley MT, Roopra A, Sutula TP (2009) Anticonvulsant and antiepileptic actions of 2-deoxy-D-glucose in epilepsy models. *Ann Neurol* 65: 435 – 447

Sullivan PF, Geschwind DH (2019) Defining the genetic, genomic, cellular, and diagnostic architectures of psychiatric disorders. *Cell* 177: 162 – 183

Tuchman R (2017) What is the relationship between autism Spectrum disorders and epilepsy? *Semin Pediatr Neurol* 24: 292 – 300

Upadhyay J, Patra J, Tiwari N, Salankar N, Ansari MN, Ahmad W (2021) Dysregulation of multiple signaling neurodevelopmental pathways during embryogenesis: a possible cause of autism spectrum disorder. *Cell* 10: 958

Vallee A, Vallee JN (2018) Warburg effect hypothesis in autism Spectrum disorders. *Mol Brain* 11: 1

Wang X, Xu Q, Bey AL, Lee Y, Jiang YH (2014) Transcriptional and functional complexity of Shank3 provides a molecular framework to understand the phenotypic heterogeneity of SHANK3 causing autism and Shank3 mutant mice. *Mol Autism* 5: 30

Wang M, Liu X, Hou Y, Zhang H, Kang J, Wang F, Zhao Y, Chen J, Liu X, Wang Y *et al* (2019) Decrease of GSK-3beta activity in the anterior cingulate cortex of Shank3b (−/−) mice contributes to synaptic and social deficiency. *Front Cell Neurosci* 13: 447

Yellen G (2018) Fueling thought: management of glycolysis and oxidative phosphorylation in neuronal metabolism. *J Cell Biol* 217: 2235 – 2246

© 2023 The Authors

Yizhar O, Fenno LE, Prigge M, Schneider F, Davidson TJ, O'Shea DJ, Sohal VS, Goshen I, Finkelstein J, Paz JT *et al* (2011) Neocortical excitation/inhibition balance in information processing and social dysfunction. *Nature* 477: 171–178

Zhou H, Xu X, Yan W, Zou X, Wu L, Luo X, Li T, Huang Y, Guan H, Chen X *et al* (2020) Prevalence of autism Spectrum disorder in China: a Nationwide multi-center population-based study among children aged 6 to 12 years. *Neurosci Bull* 36: 961–971

Zuo Q, He J, Zhang S, Wang H, Jin G, Jin H, Cheng Z, Tao X, Yu C, Li B *et al* (2021) PPARgamma coactivator-1alpha suppresses metastasis of hepatocellular carcinoma by inhibiting Warburg effect by PPARgamma-dependent WNT/beta-catenin/pyruvate dehydrogenase kinase isozyme 1 Axis. *Hepatology* 73: 644–660

License: This is an open access article under the terms of the Creative Commons Attribution License, which permits use, distribution and reproduction in any medium, provided the original work is properly cited.

Expanded View Figures

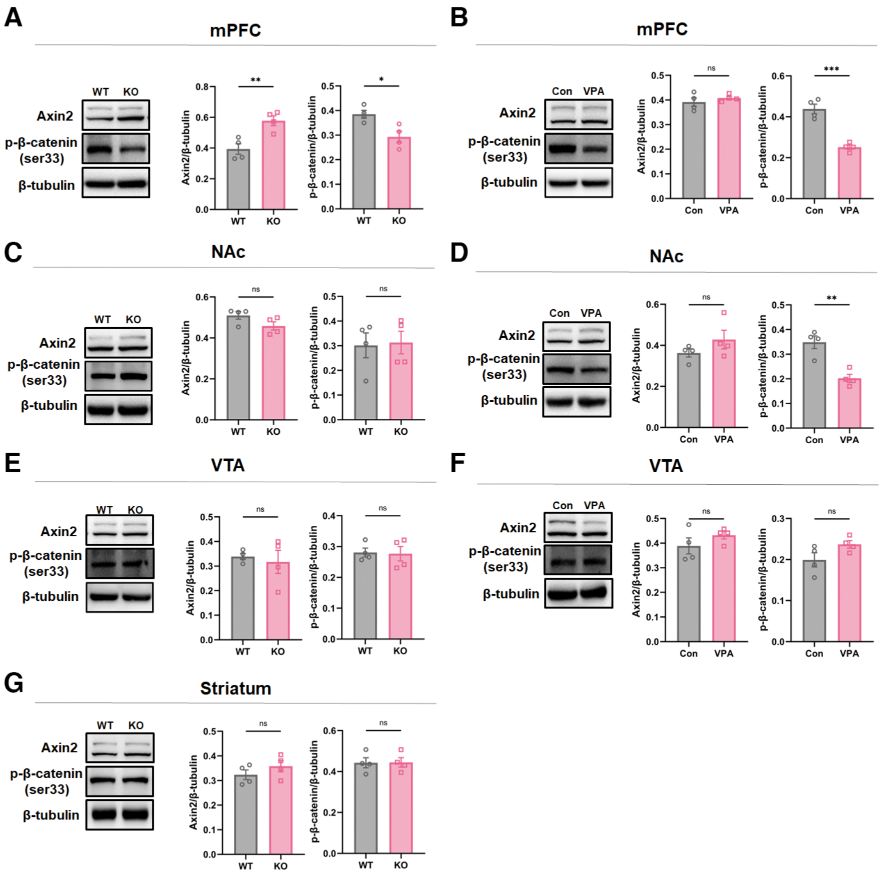

Figure EV1. Expression of Wnt signaling components in other brain regions.

A Western blotting of Axin2 and p-β-catenin(S33) in the mPFC of WT and *Shank3*$^{-/-}$ mice.
B Western blotting of Axin2 and p-β-catenin(S33) in the mPFC of control and VPA-treated mice.
C Western blotting of Axin2 and p-β-catenin(S33) in the NAc of WT and *Shank3*$^{-/-}$ mice.
D Western blotting of Axin2 and p-β-catenin(S33) in the NAc of control VPA-treated mice.
E Western blotting of Axin2 and p-β-catenin(S33) in the VTA of WT and *Shank3*$^{-/-}$ mice.
F Western blotting of Axin2 and p-β-catenin(S33) in the VTA of control VPA-treated mice.
G Western blotting of Axin2 and p-β-catenin(S33) in the striatum of WT and *Shank3*$^{-/-}$ mice.

Data information: $N = 4$ samples from 12 mice per group. Mean ratio ± SEM. Two-tailed unpaired *t*-test (A, B, p-β-catenin in C, D-G). Mann–Whitney *U* test (Axin2 in C) $^*P < 0.05$. $^{**}P < 0.01$. $^{***}P < 0.001$. WT, wild type. KO, *Shank3*$^{-/-}$.
Source data are available online for this figure.

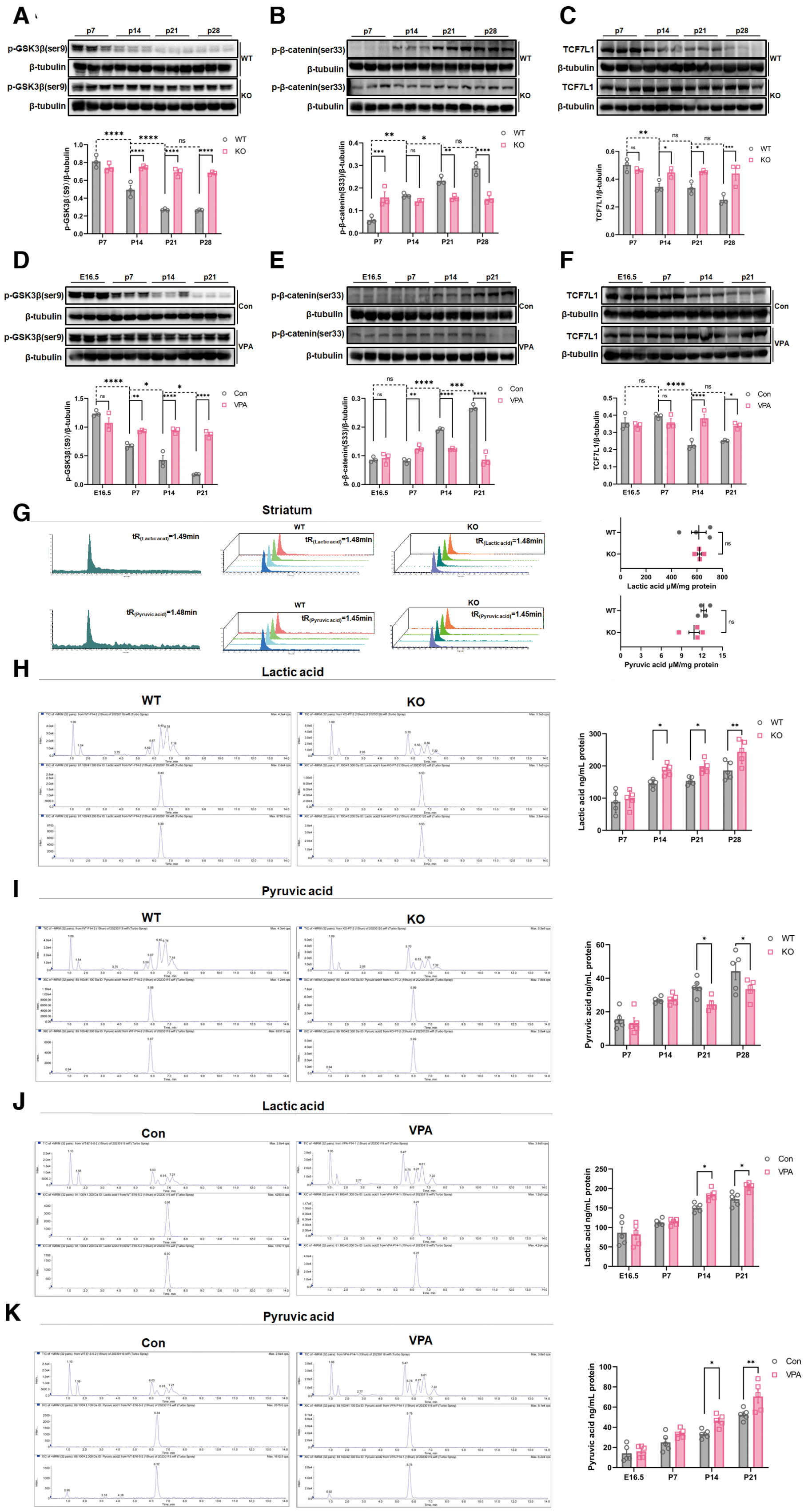

Figure EV2. Developmental changes of Wnt signaling and glycolysis.

A–C Western blotting of p-GSK3β(S9), p-β-catenin (S33) and TCF7L1 in the ACC of WT and *Shank3*$^{-/-}$ mice at P7, P14, P21 and P28.

D–F Western blotting of p-GSK3β(S9), p-β-catenin (S33) and TCF7L1 in the ACC of control and VPA-treated mice at E16.5, P7, P14 and P21.

G LS-MS measurement of lactic acid and pyruvic acid in the striatum of WT and *Shank3*$^{-/-}$ mice.

H, I LS-MS/MS measurement of lactic acid and pyruvic acid in the ACC of WT and *Shank3*$^{-/-}$ mice at P7, P14, P21 and P28.

J, K LS-MS/MS measurement of lactic acid and pyruvic acid in the ACC of control and VPA-treated mice at E16.5, P7, P14 and P21.

Data information: $N = 4$ samples from 12 mice per group in (A-F), 4 samples from 16 mice per group in (G), 5 samples from 20 mice per group in (H-K). Mean ratio ± SEM. Two-way repeated measurement ANOVA and Sidak's multiple comparisons test (A–F, H–K). Two-tailed unpaired *t*-test (G). $^{*}P < 0.05$, $^{**}P < 0.01$, $^{***}P < 0.001$, $^{****}P < 0.0001$. WT, wild type. KO, *Shank3*$^{-/-}$. Con, control.
Source data are available online for this figure.

 © 2023 The Authors

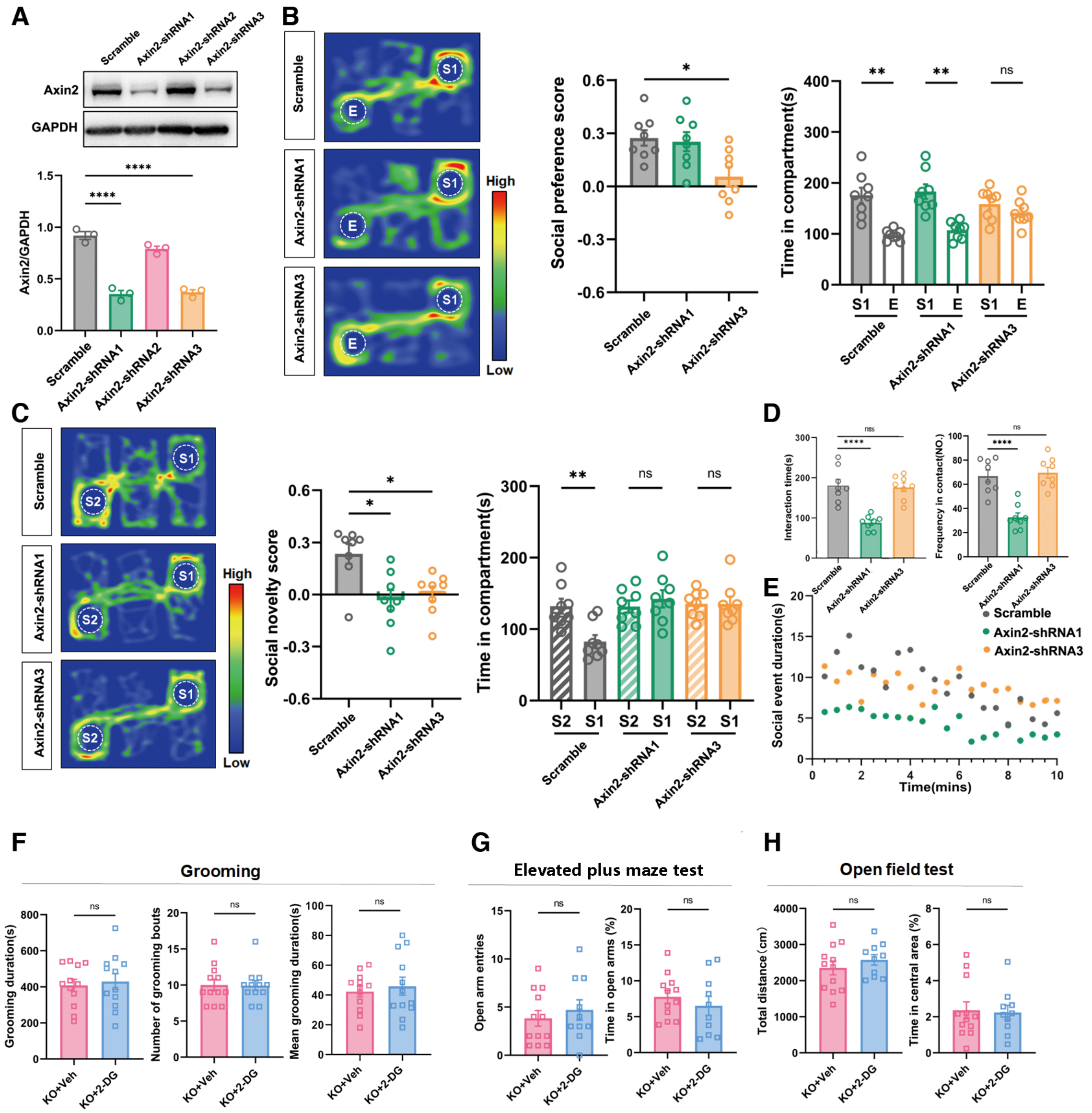

Figure EV3. Effects of knocking down Axin2 in on the social behavior of WT mice and 2-DG treatment on repetitive/anxiety-like behaviors of *Shank3*$^{-/-}$ mice.

A Western blotting verification of 3 Axin2-shRNA in ACC. Axin2-shRNA1 and Axin2-shRNA3 are efficient in knocking down Axin2.

B–E 3-chamber and resident-intruder assay of mice treated with scrambled RNA, Axin2-shRNA1 and Axin2-shRNA3. Notice the social impairment effects of Axin2-shRNA1 and Axin2-shRNA3.

F Grooming test of *Shank3*$^{-/-}$ mice treated with or without 2-DG.

G, H Elevated plus maze test and open-field test of *Shank3*$^{-/-}$ mice treated with or without 2-DG.

Data information: $N = 3$ mice in (A), 8 mice (B–D) and 10–12 mice (E–G) mice per group. Mean ratio ± SEM. One-way ANOVA with Tukey's multiple comparison test (A, D, social preference score in (B)). Kruskal–Wallis *H* test with Dunn's multiple comparison test (social novelty score in (C)). Paired *t*-test (time in compartment in Axin2-shRNA3 treated mice in (B), time in compartment in (C)). Wilcoxon signed-rank test (time in compartment of scrambled RNA and Axin2-shRNA3 treated mice in (B)). Two-tailed unpaired *t*-test and Mann–Whitney *U* test (F–H). $*P < 0.05$. $**P < 0.01$, $****P < 0.0001$.
Source data are available online for this figure.

Figure EV4. Effects of XAV939 on the glycolysis/oxidative phosphorylation, social behavior, repetitive and anxiety-like behavior of ASD mice.

A ECAR assay of VPA-neurons treated with or without XAV939. Notice the decrease of glycolysis in XAV939-treated cells.
B OCR assay of VPA-neurons treated with or without XAV939. Notice the increase of oxidative phosphorylation in XAV939-treated cells.
C–E 3-chamber assay of *Shank3*$^{-/-}$ mice at 1 week following the last administration of XAV939. Notice the social improvement in XAV939-treated cells.
F–H Grooming test, elevated plus maze test and open-field test of *Shank3*$^{-/-}$ mice treated with or without XAV939.
I–K Grooming test, elevated plus maze test and open-field test of VPA-ASD mice treated with or without XAV939.

Data information: N = 4–5 batches of cells per group (A, B), 8 mice (D, E) and 7–8 mice (F–K) per group. Mean ratio ± SEM. Two-tailed unpaired t-test (max ECAR in B). Mann–Whitney U test (ECAR in (B), Open arm entries in (J) and (A)). $^*P < 0.05$. $^{**}P < 0.01$. Two-tailed unpaired t-test (social novelty score in E, F–K). Paired t-test (time in compartment in D and E). Wilcoxon signed-rank test (social preference scores in (E)). $^*P < 0.05$, $^{**}P < 0.01$.
Source data are available online for this figure.

 © 2023 The Authors

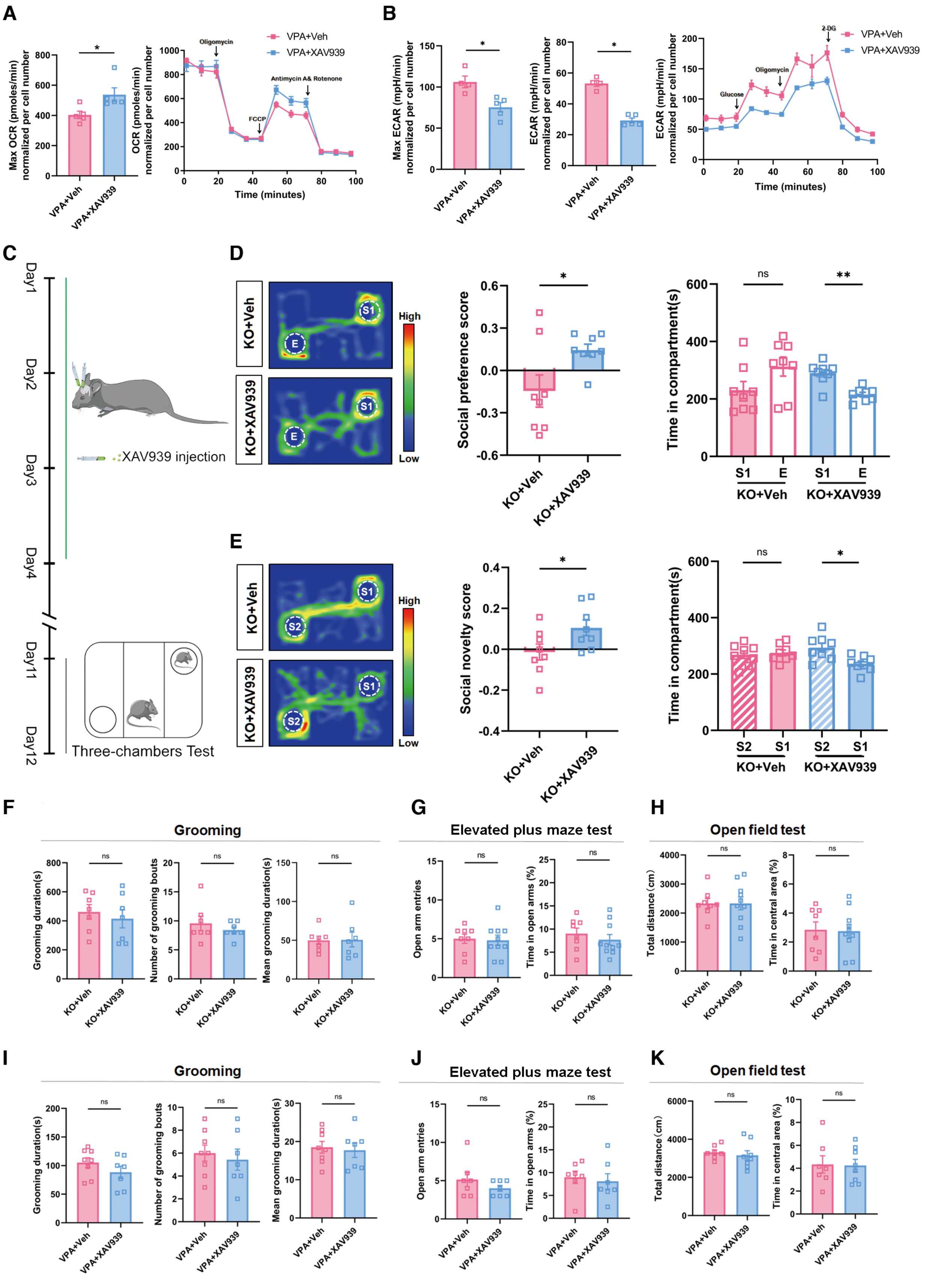
A
Max OCR (pmoles/min) normalized per cell number
OCR (pmoles/min) normalized per cell number
Oligomycin
FCCP
Antimycin A& Rotenone
VPA+Veh
VPA+XAV939
Time (minutes)
B
Max ECAR (mpH/min) normalized per cell number
ECAR (mpH/min) normalized per cell number
Glucose
Oligomycin
2-DG
C
Day1
Day2
Day3
Day4
Day11
Day12
XAV939 injection
Three-chambers Test
D
KO+Veh
KO+XAV939
High
Low
S1
E
Social preference score
Time in compartment(s)
ns
E
S2
Social novelty score
F
Grooming
Grooming duration(s)
Number of grooming bouts
Mean grooming duration(s)
G
Elevated plus maze test
Open arm entries
Time in open arms (%)
H
Open field test
Total distance (cm)
Time in central area (%)
I
J
K

Figure EV4.

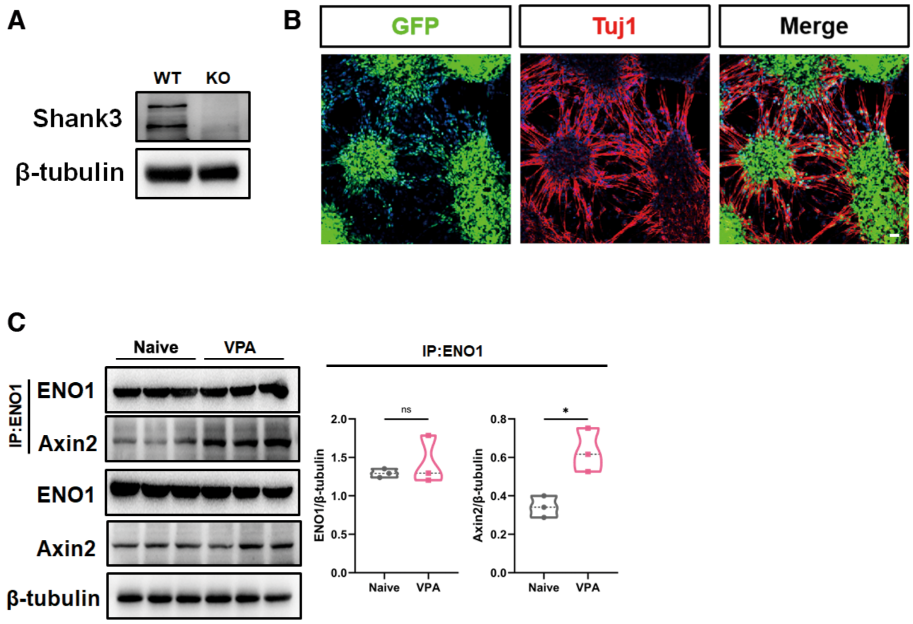

Figure EV5. Shank3 mutation and neural induction in human ESCs and interaction of Axin2/ENO1 in naïve and VPA-pretreated ESCs.

A Western blotting of Shank3 in WT and *Shank3* mutant human neurons.

B Immunocytochemistry of Tuj-1 in human ESCs after neural induction. At this stage, human neurons were used for experiments. Bar = 5 µm.

C Protein CO-IP of Axin2/ENO1 in naïve and VPA-treated human neurons.

Data information: *N* = 3 batches of cells. Two-tailed unpaired *t*-test. *$P < 0.05$.
Source data are available online for this figure.

 © 2023 The Authors
