# Supplementary figures and images for: Axin2 coupled excessive Wnt‐glycolysis signaling mediates social defect in autism spectrum disorders
Source: EMBO Mol Med. 2023 Apr 20;15(6):e17101. doi: 10.15252/emmm.202217101 (PMC10245038; doi:10.15252/emmm.202217101)

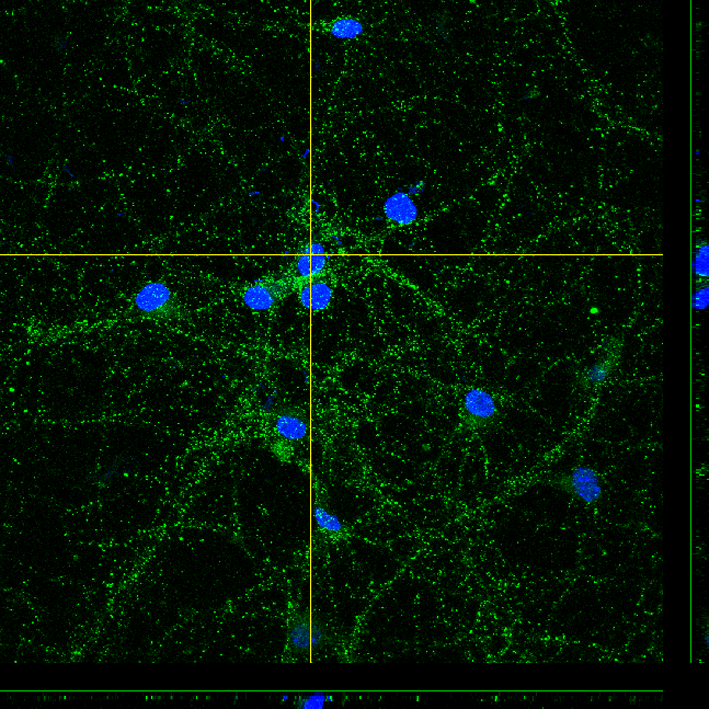

Supplement: Supplementary file 6 — Source Data for Figure 1 [file EMMM-15-e17101-s014.zip › Figure1/Image data-Fig1D/IgG-6h.tif]

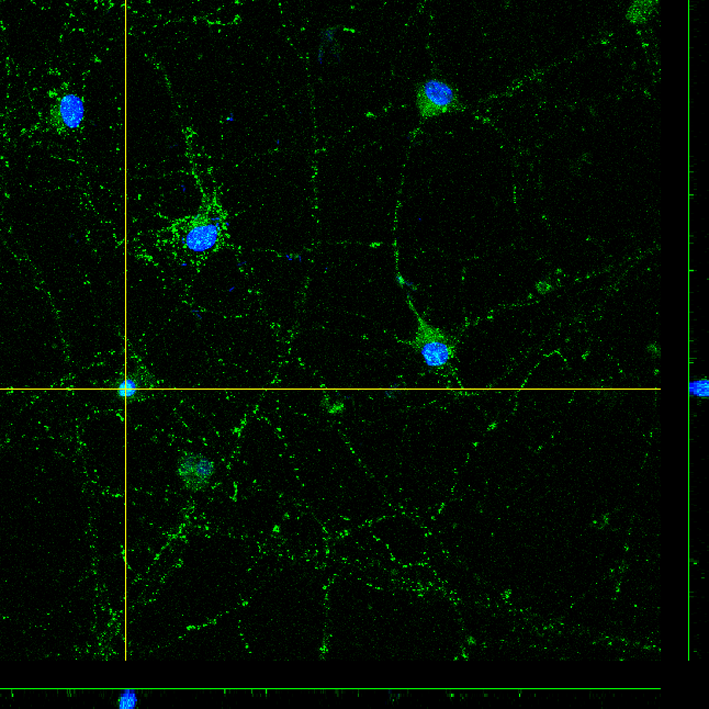

Supplement: Supplementary file 6 — Source Data for Figure 1 [file EMMM-15-e17101-s014.zip › Figure1/Image data-Fig1D/Anti-SHANK3-6h.tif]

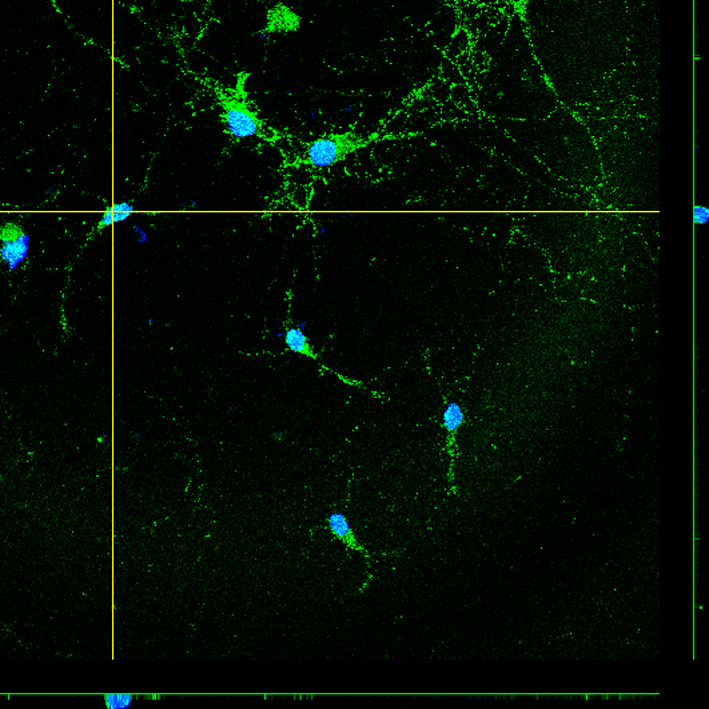

Supplement: Supplementary file 6 — Source Data for Figure 1 [file EMMM-15-e17101-s014.zip › Figure1/Image data-Fig1D/Anti-SHANK3-12h.tif]

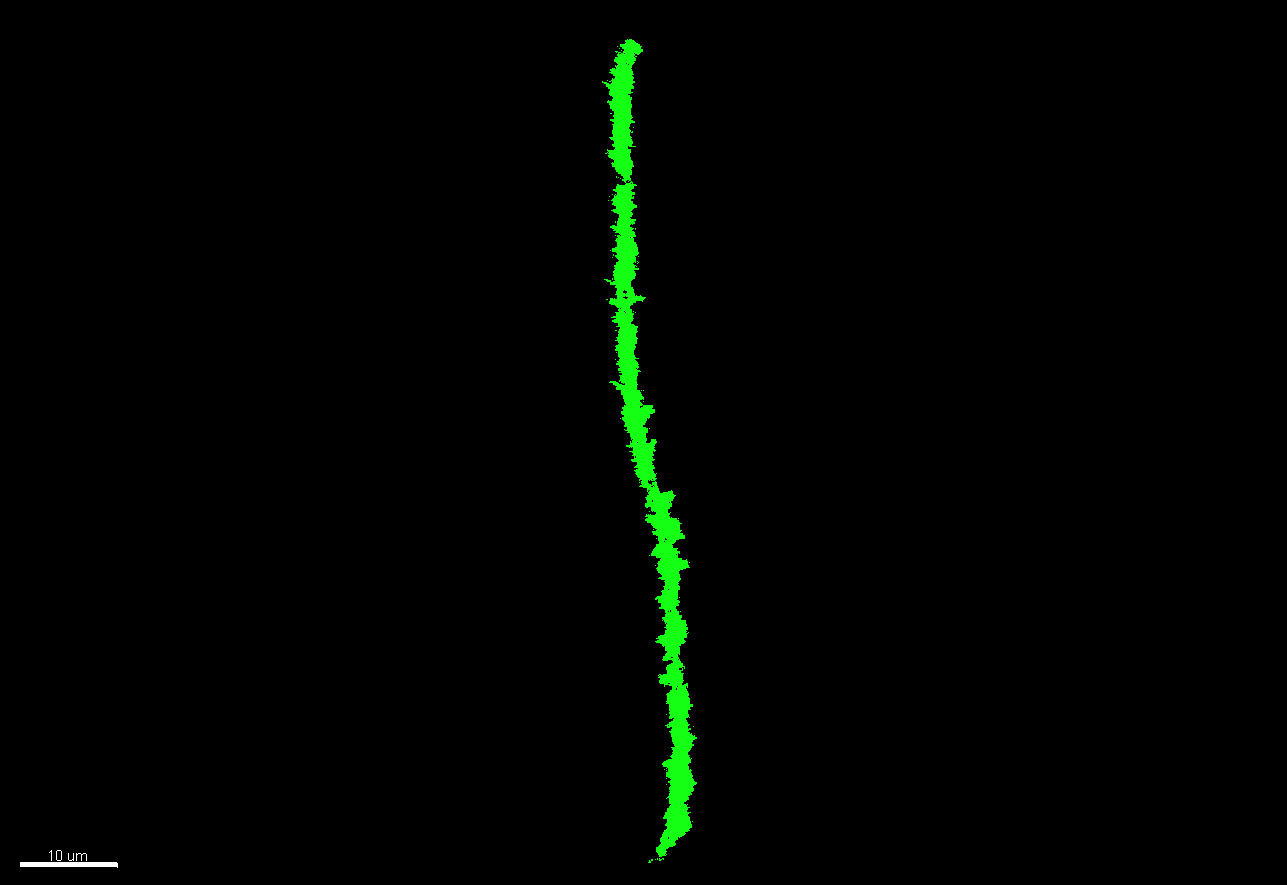

Supplement: Supplementary file 8 — Source Data for Figure 3 [file EMMM-15-e17101-s001.zip › Figure3/Image data-Fig3C/GFP+EX3/Typical image of spine.tif]

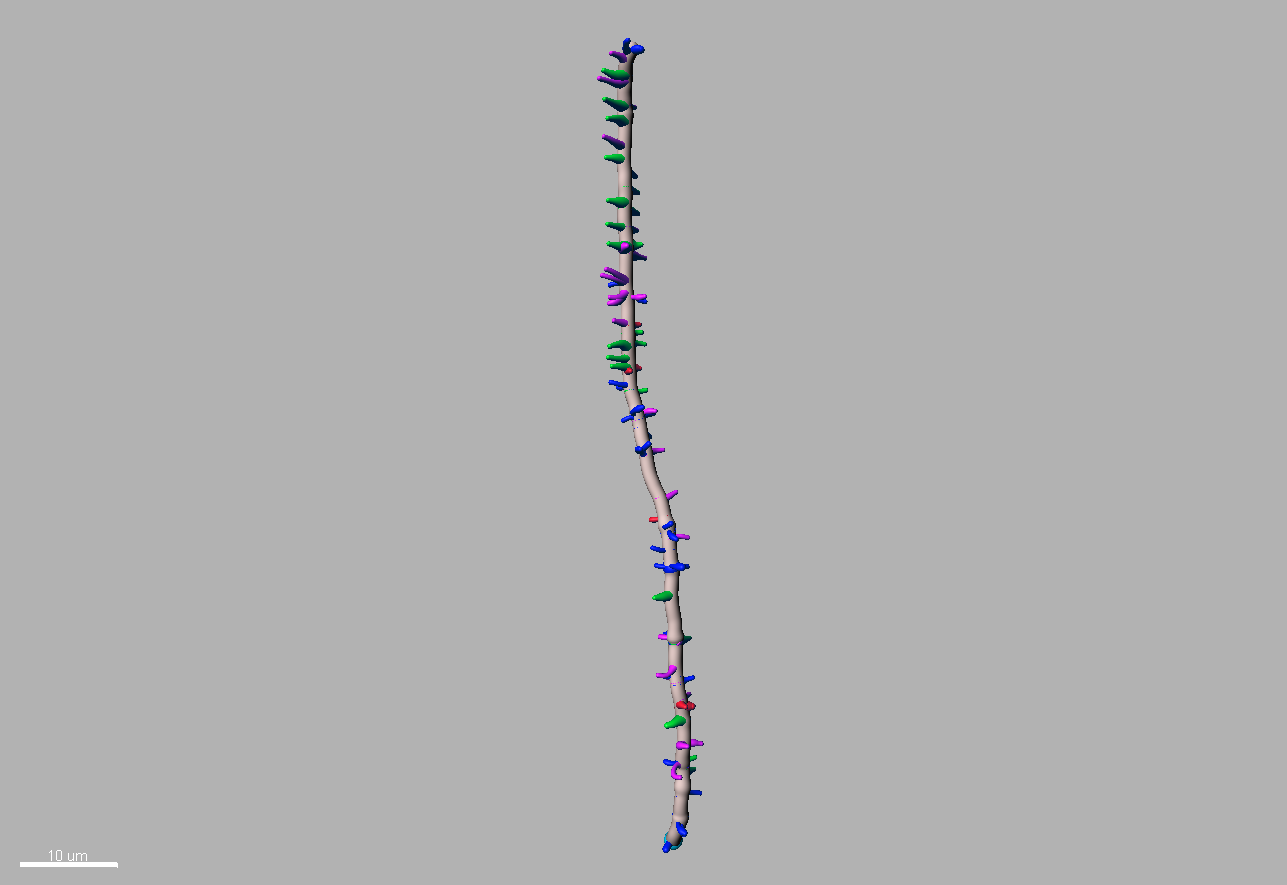

Supplement: Supplementary file 8 — Source Data for Figure 3 [file EMMM-15-e17101-s001.zip › Figure3/Image data-Fig3C/GFP+EX3/3D-reconstructed spine.tif]

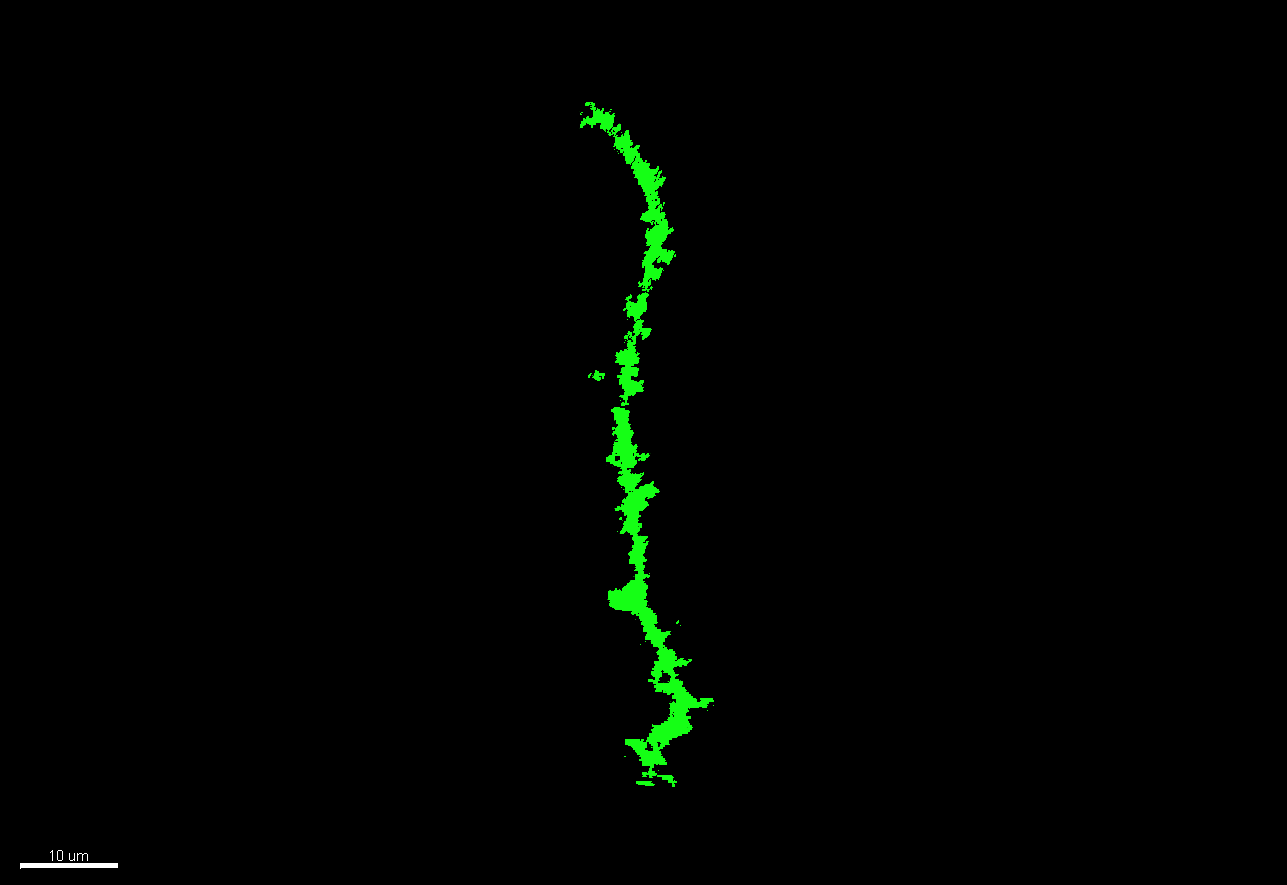

Supplement: Supplementary file 8 — Source Data for Figure 3 [file EMMM-15-e17101-s001.zip › Figure3/Image data-Fig3C/CaMK-Cre+EX3/Typical image of spine.tif]

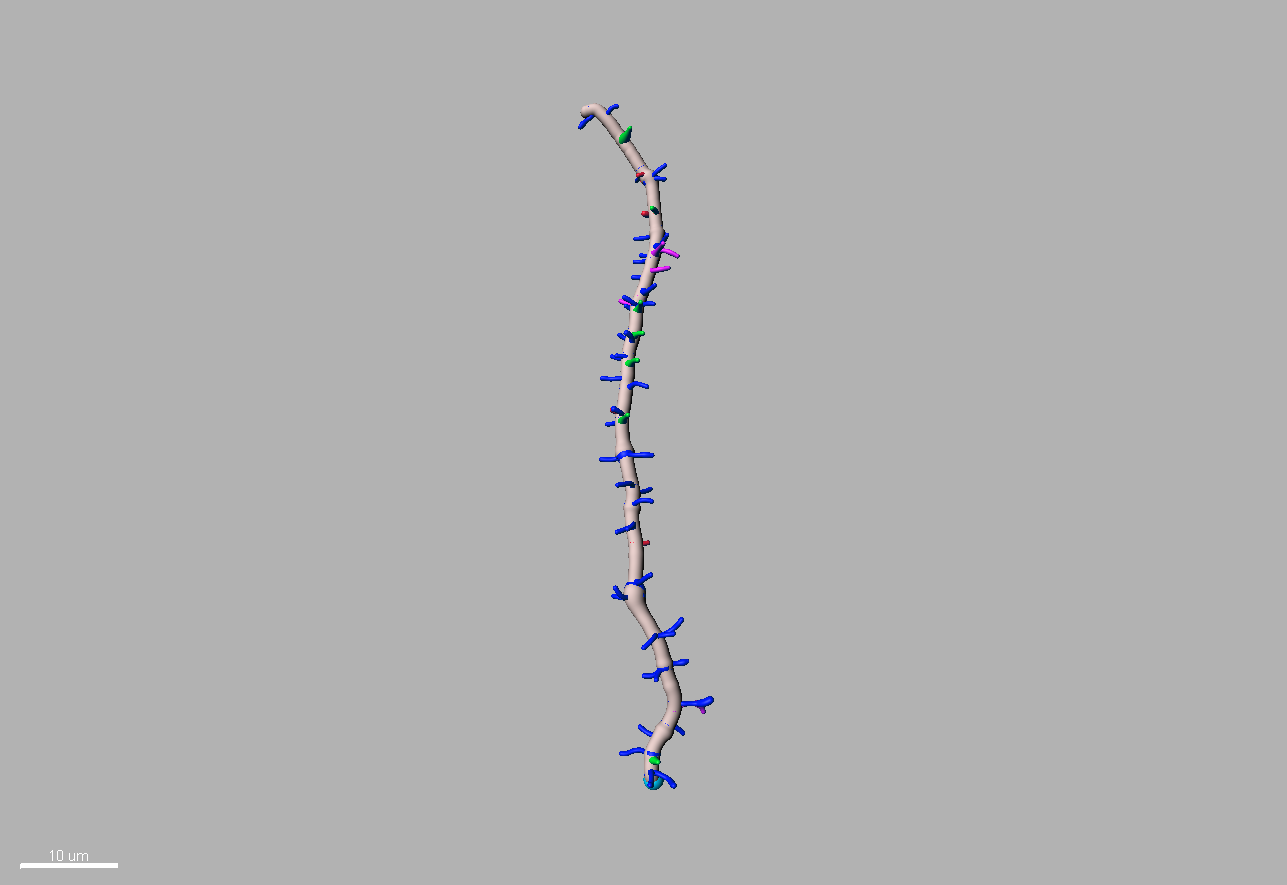

Supplement: Supplementary file 8 — Source Data for Figure 3 [file EMMM-15-e17101-s001.zip › Figure3/Image data-Fig3C/CaMK-Cre+EX3/3D-reconstructed spine.tif]

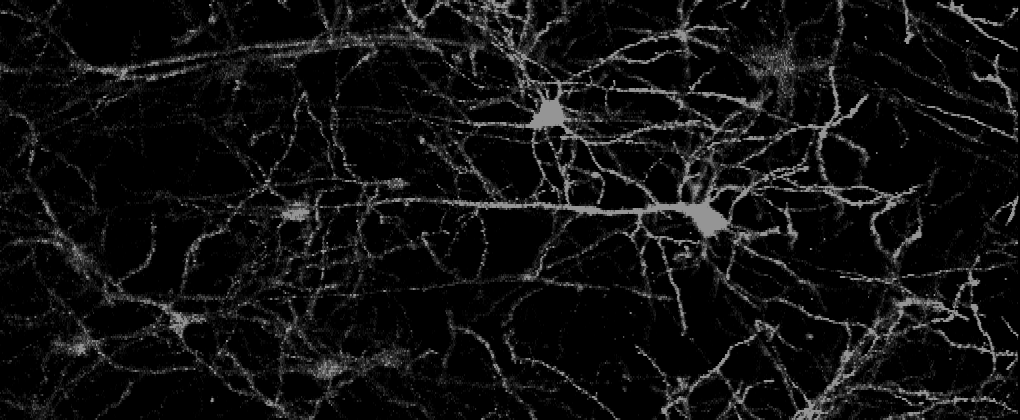

Supplement: Supplementary file 8 — Source Data for Figure 3 [file EMMM-15-e17101-s001.zip › Figure3/Image data-Fig3B/GFP+EX3/Typical image of neuron.tif]

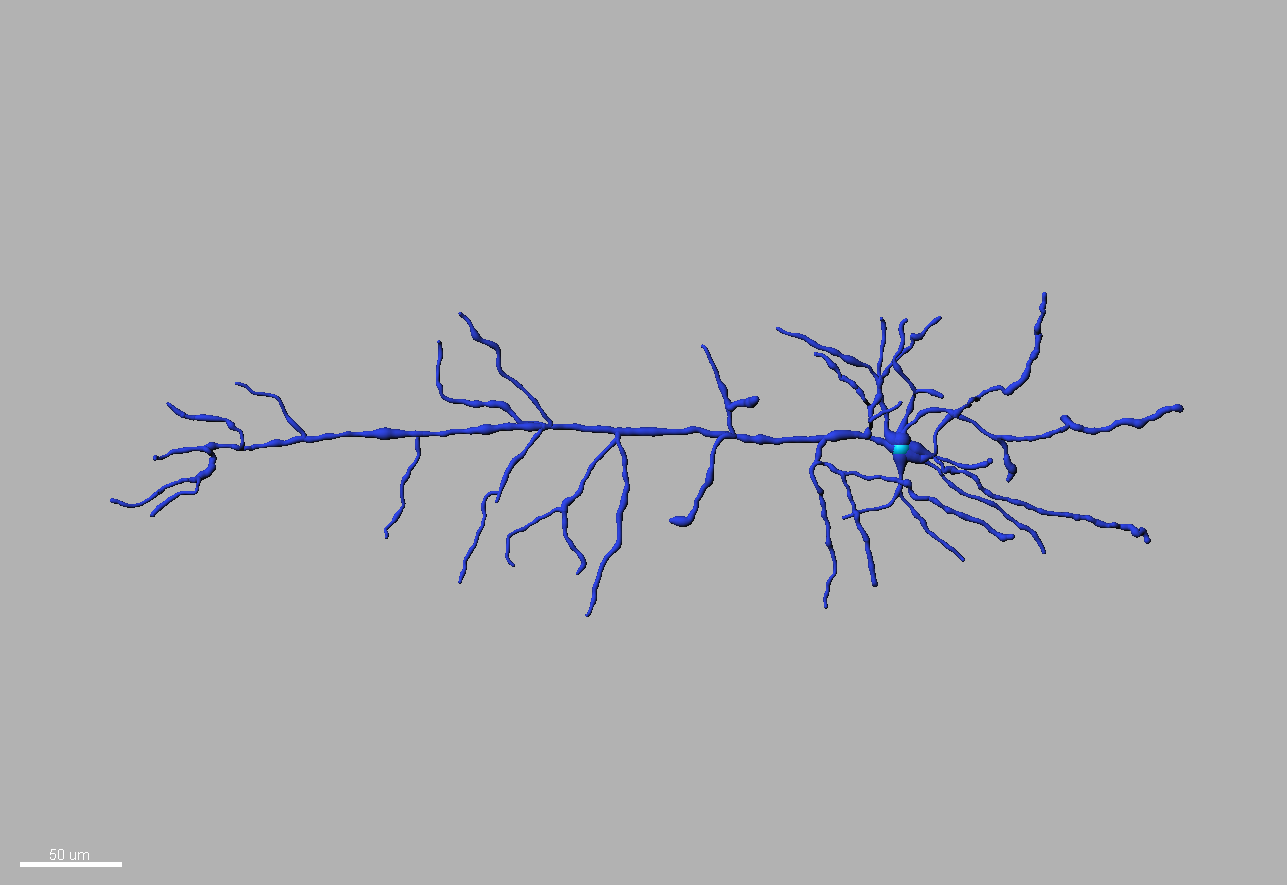

Supplement: Supplementary file 8 — Source Data for Figure 3 [file EMMM-15-e17101-s001.zip › Figure3/Image data-Fig3B/GFP+EX3/3D-reconstructed neuron.tif]

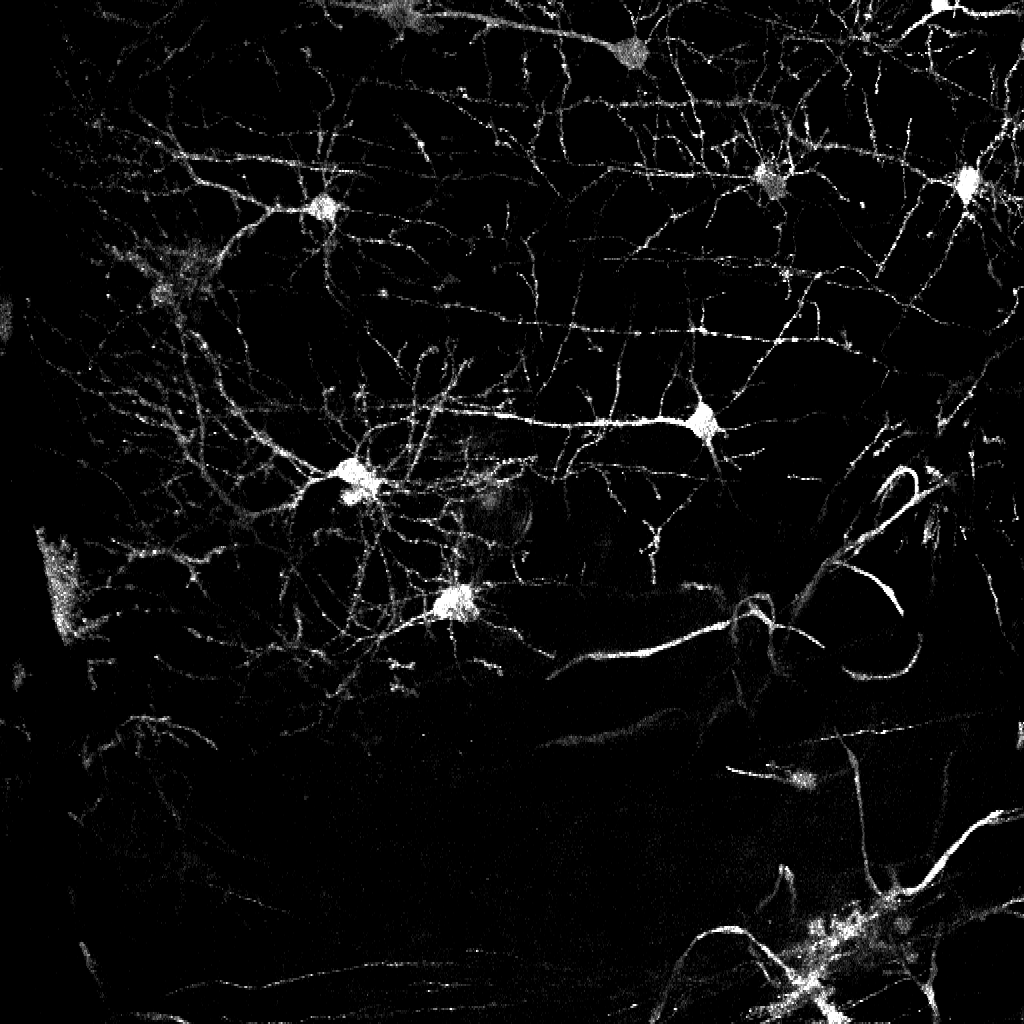

Supplement: Supplementary file 8 — Source Data for Figure 3 [file EMMM-15-e17101-s001.zip › Figure3/Image data-Fig3B/CaMK-Cre+EX3/Typical image of neuron.tif]

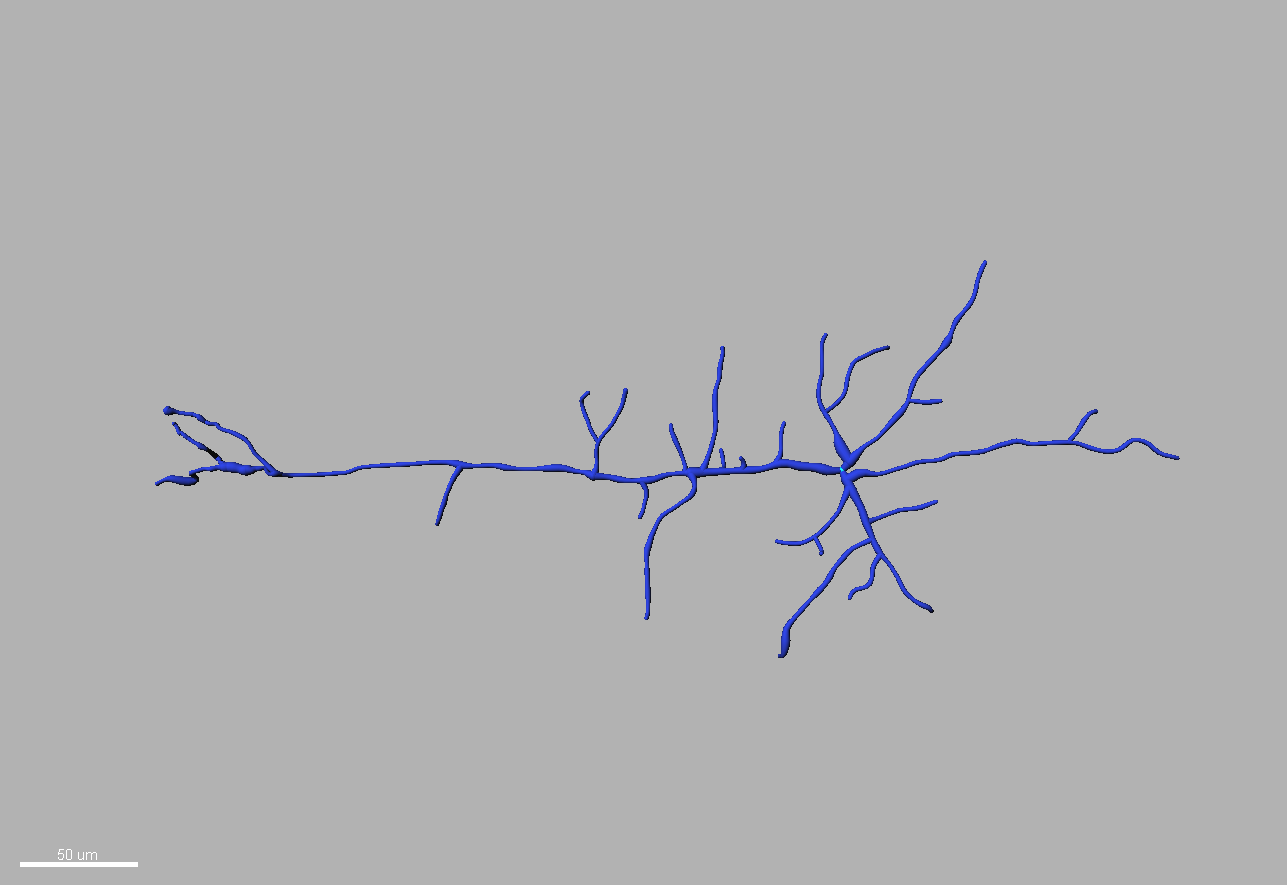

Supplement: Supplementary file 8 — Source Data for Figure 3 [file EMMM-15-e17101-s001.zip › Figure3/Image data-Fig3B/CaMK-Cre+EX3/3D-reconstructed neuron.tif]

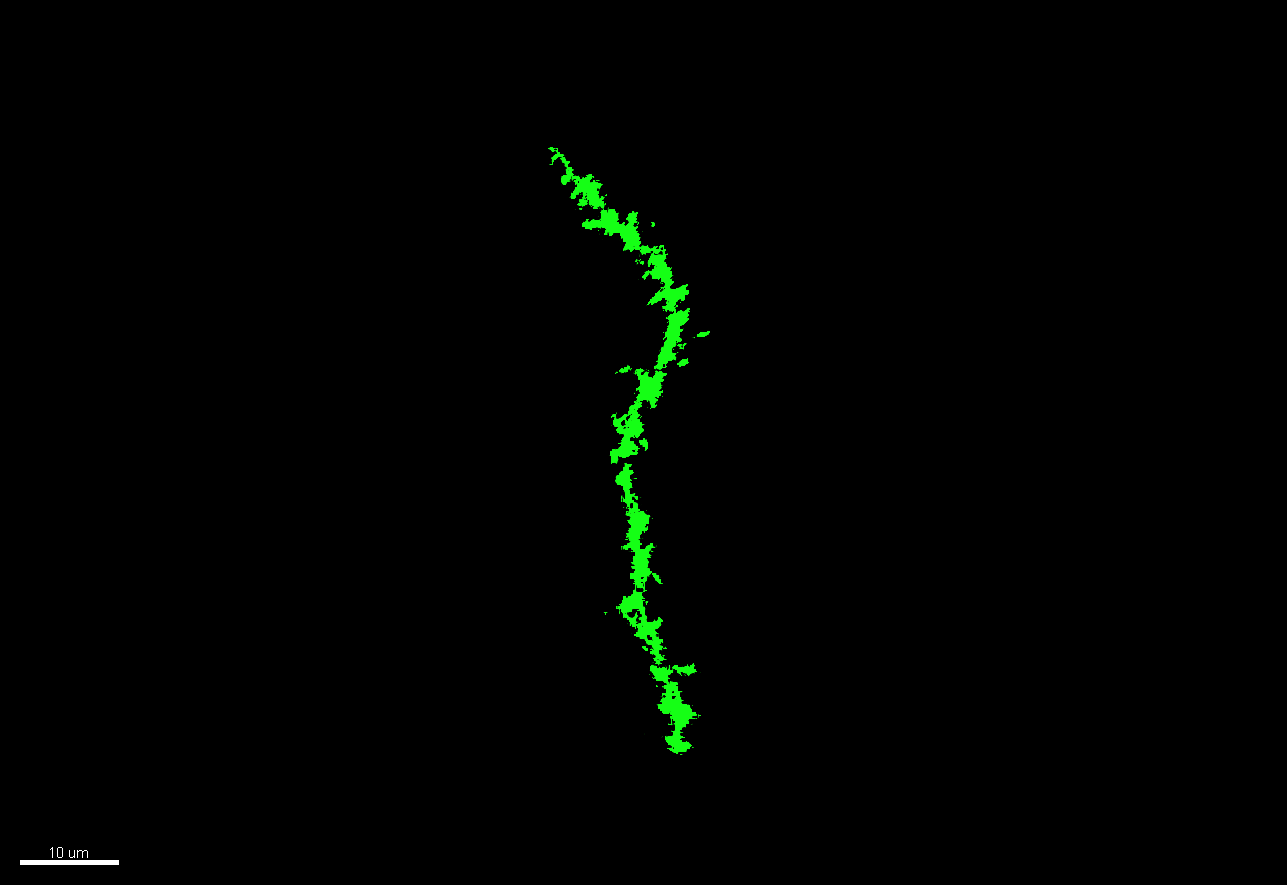

Supplement: Supplementary file 9 — Source Data for Figure 4 [file EMMM-15-e17101-s003.zip › Figure4/Image data-Fig4D/KO+2-DG/Typical image of spine.tif]

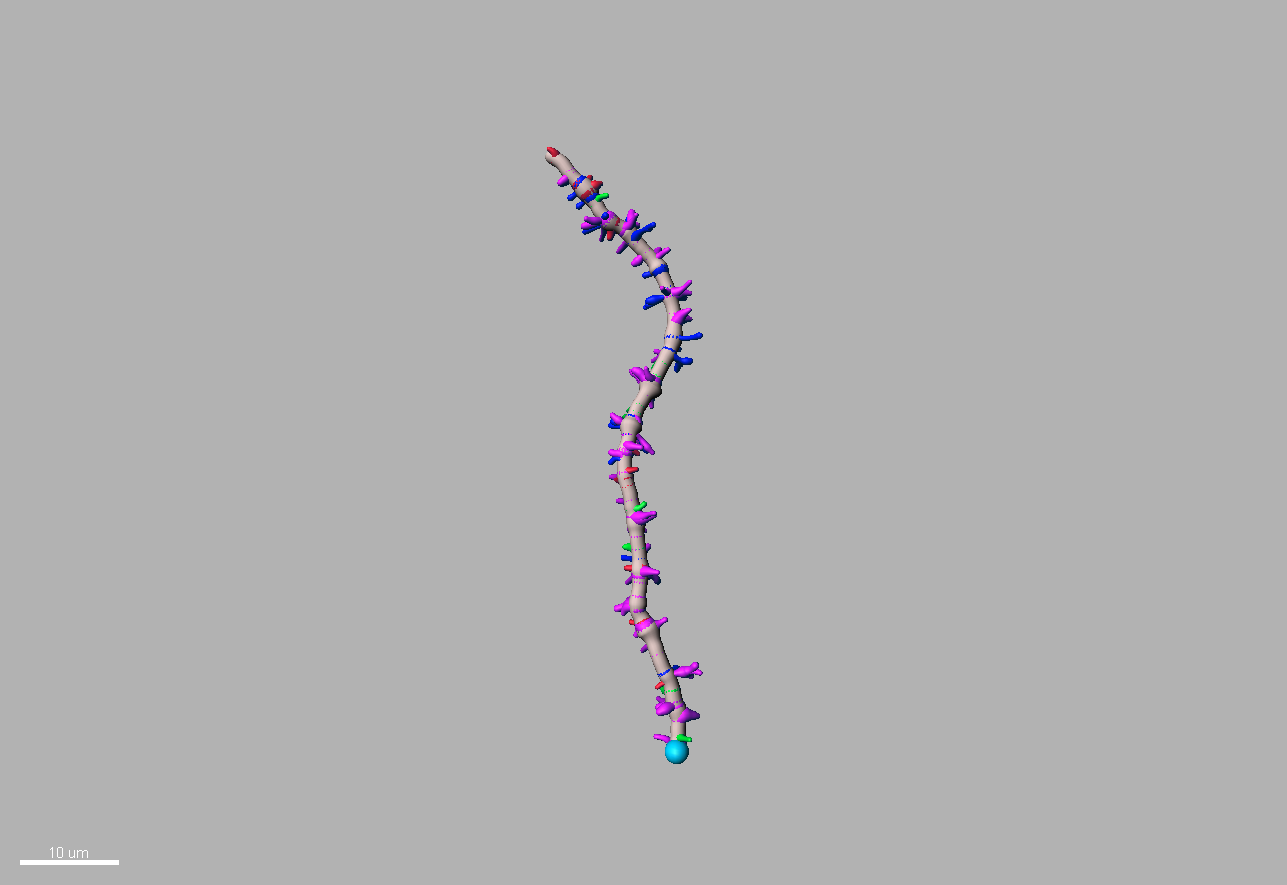

Supplement: Supplementary file 9 — Source Data for Figure 4 [file EMMM-15-e17101-s003.zip › Figure4/Image data-Fig4D/KO+2-DG/3D-reconstructed spine.tif]

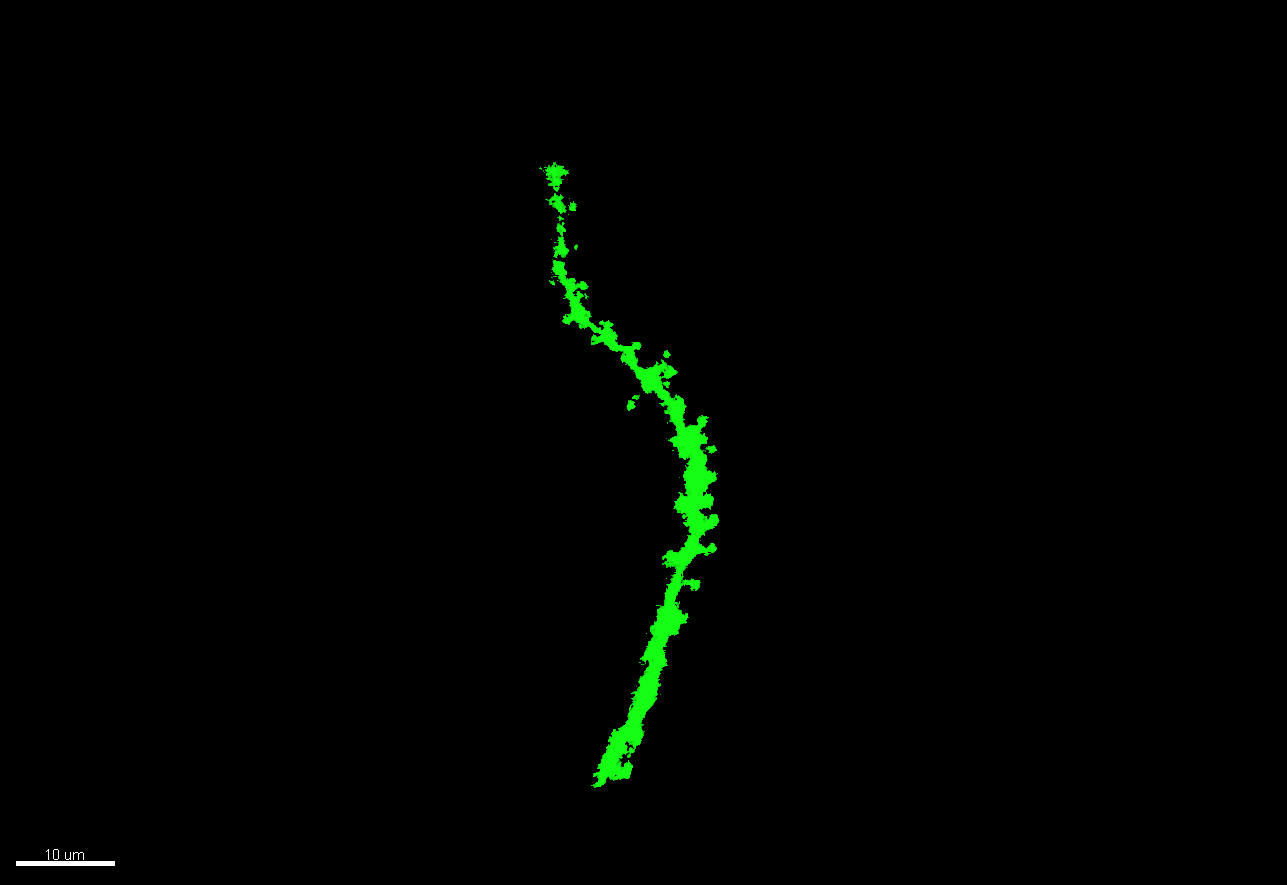

Supplement: Supplementary file 9 — Source Data for Figure 4 [file EMMM-15-e17101-s003.zip › Figure4/Image data-Fig4D/KO+Veh/Typical image of spine.tif]

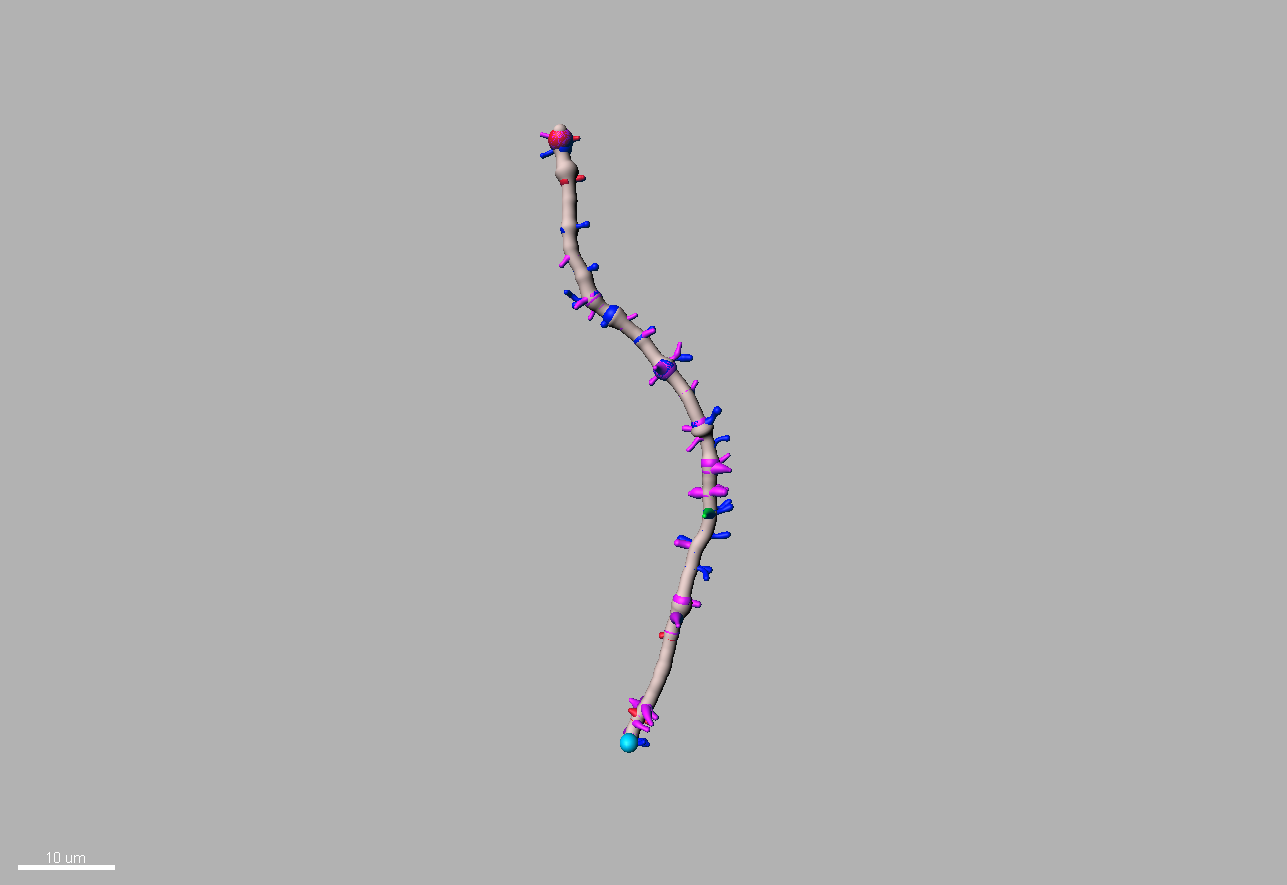

Supplement: Supplementary file 9 — Source Data for Figure 4 [file EMMM-15-e17101-s003.zip › Figure4/Image data-Fig4D/KO+Veh/3D-reconstructed spine.tif]

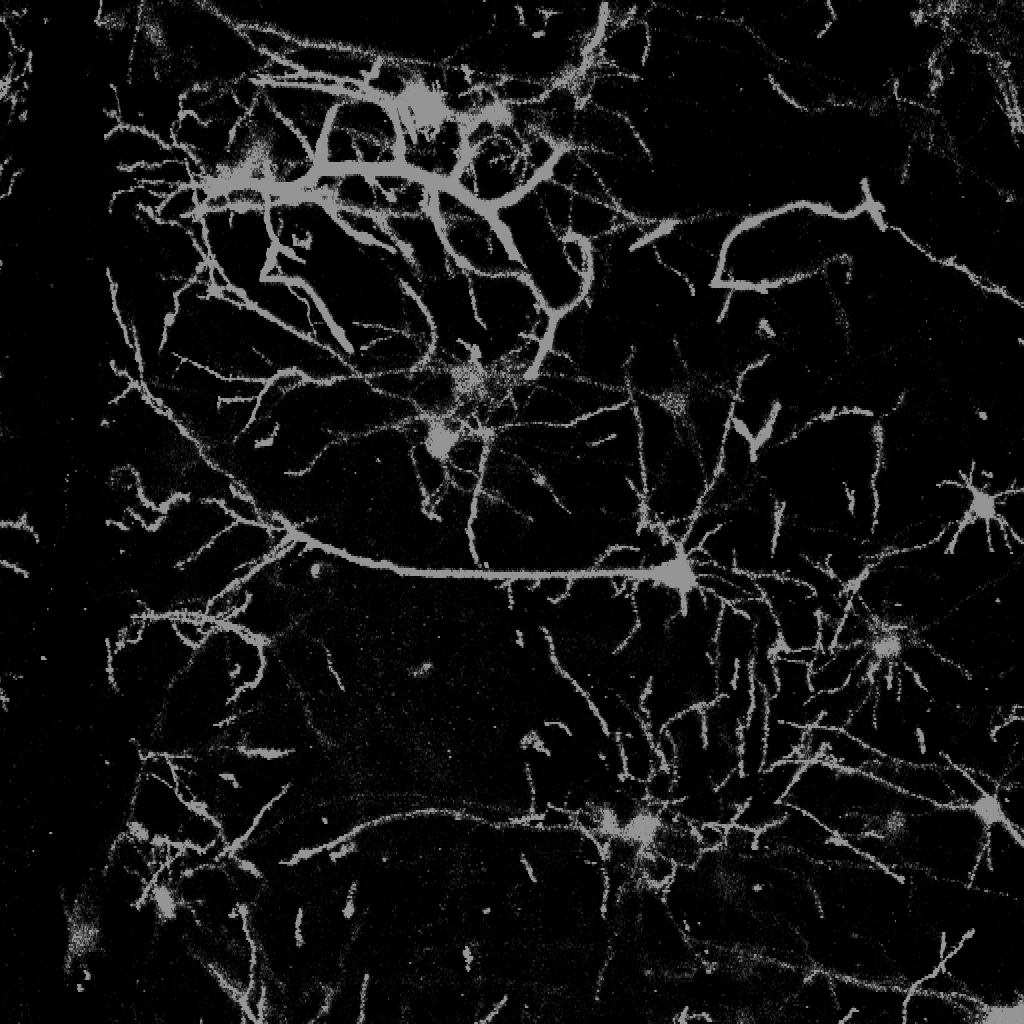

Supplement: Supplementary file 9 — Source Data for Figure 4 [file EMMM-15-e17101-s003.zip › Figure4/Image data-Fig4A/KO+2-DG/Typical image of neuron.tif]

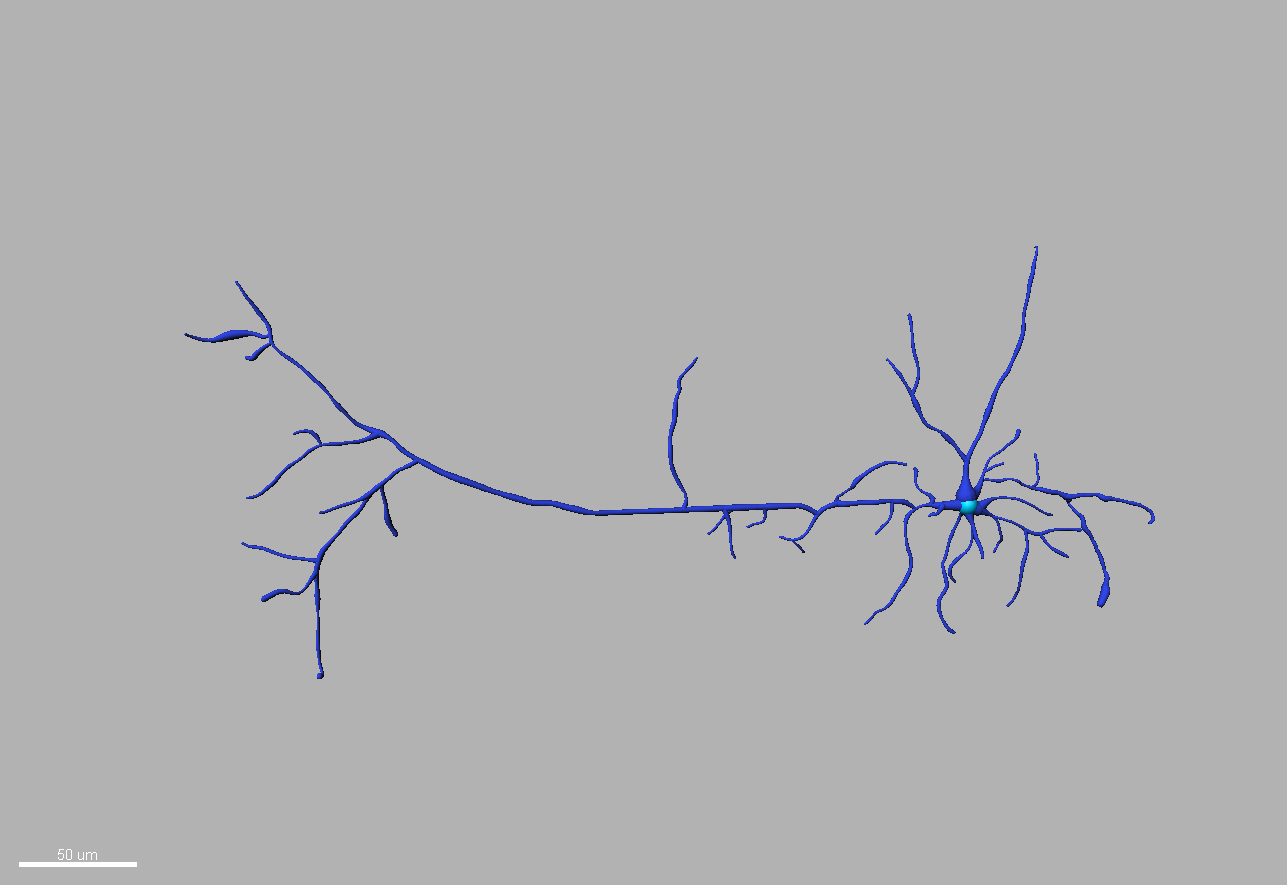

Supplement: Supplementary file 9 — Source Data for Figure 4 [file EMMM-15-e17101-s003.zip › Figure4/Image data-Fig4A/KO+2-DG/3D-reconstructed neuron.tif]

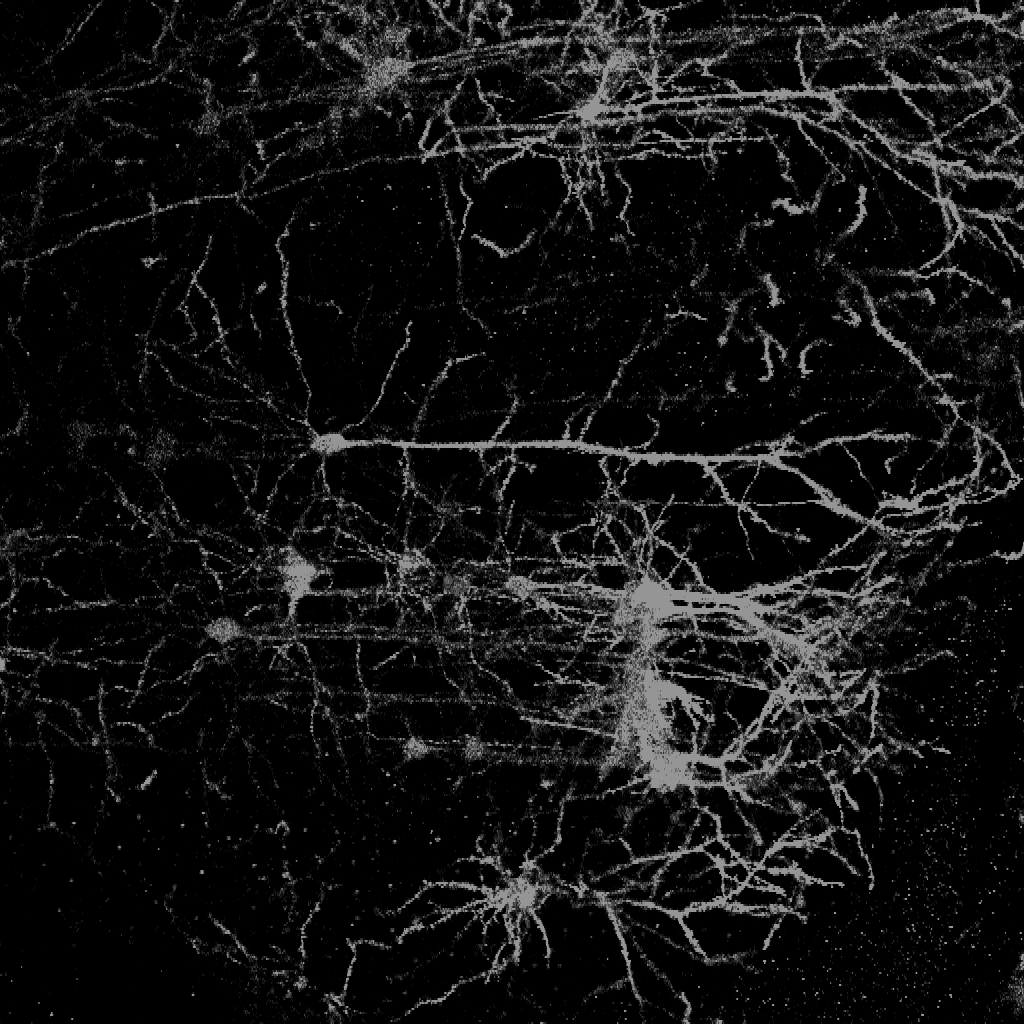

Supplement: Supplementary file 9 — Source Data for Figure 4 [file EMMM-15-e17101-s003.zip › Figure4/Image data-Fig4A/KO+Veh/Typical image of neuron.tif]

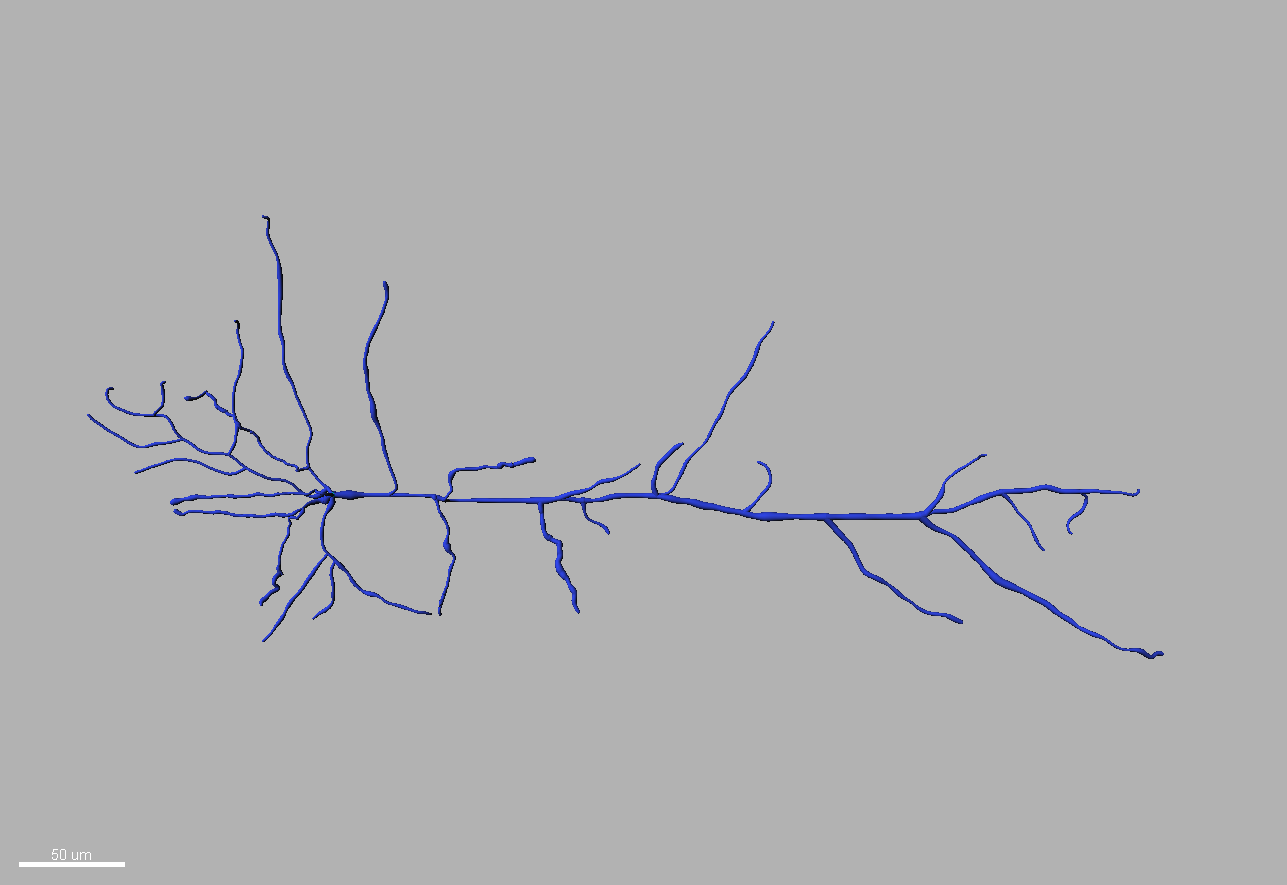

Supplement: Supplementary file 9 — Source Data for Figure 4 [file EMMM-15-e17101-s003.zip › Figure4/Image data-Fig4A/KO+Veh/3D-reconstructed neuron.tif]

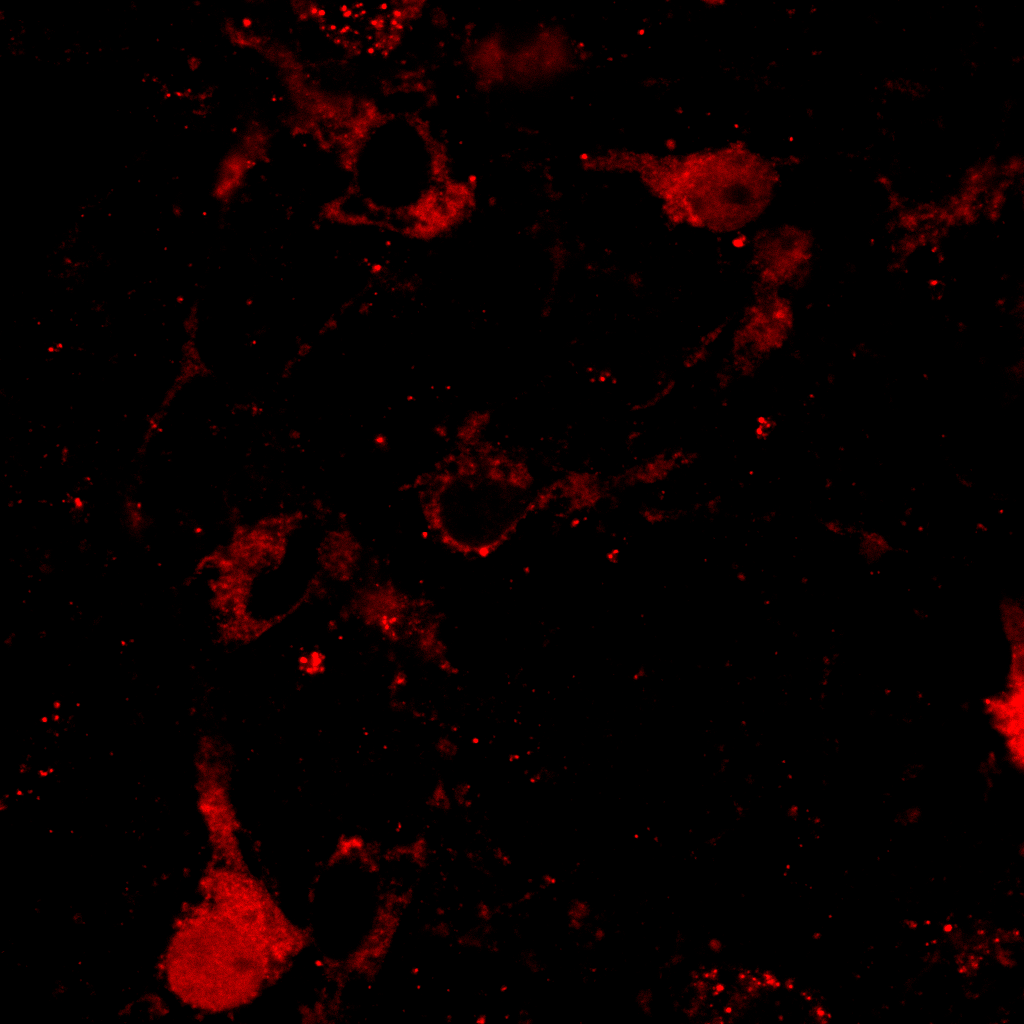

Supplement: Supplementary file 10 — Source Data for Figure 5 [file EMMM-15-e17101-s011.zip › Figure5/Image data-Fig5E/KO+XAV939/ENO1.tif]

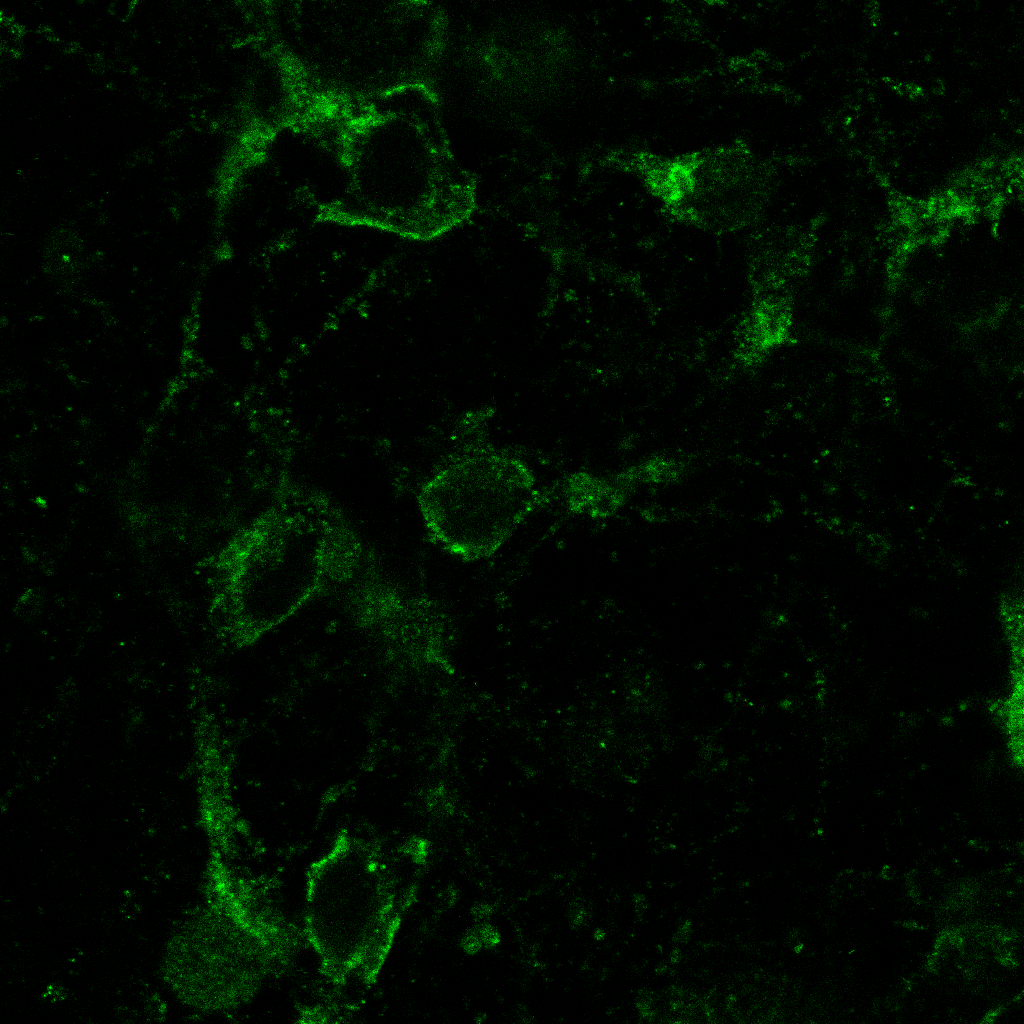

Supplement: Supplementary file 10 — Source Data for Figure 5 [file EMMM-15-e17101-s011.zip › Figure5/Image data-Fig5E/KO+XAV939/Axin2.tif]

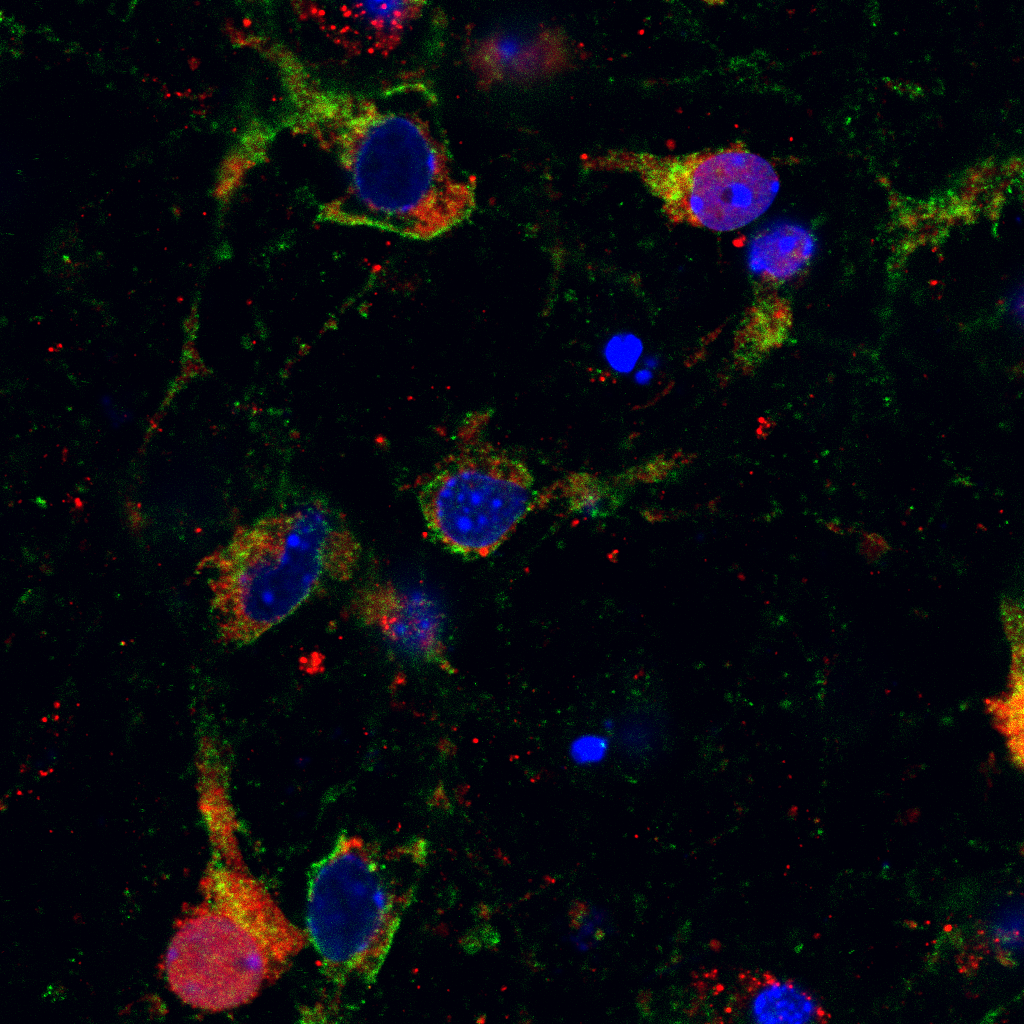

Supplement: Supplementary file 10 — Source Data for Figure 5 [file EMMM-15-e17101-s011.zip › Figure5/Image data-Fig5E/KO+XAV939/Merge.tif]

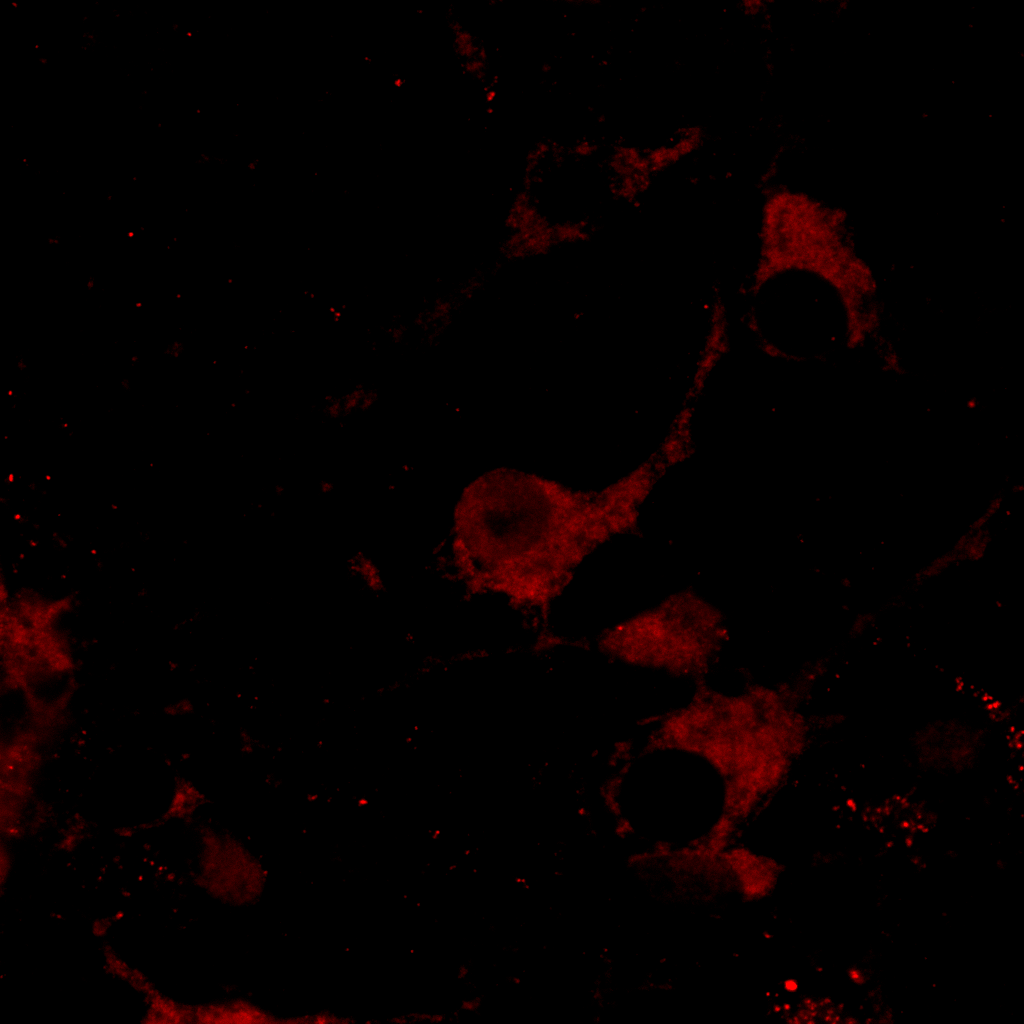

Supplement: Supplementary file 10 — Source Data for Figure 5 [file EMMM-15-e17101-s011.zip › Figure5/Image data-Fig5E/WT+Veh/ENO1.tif]

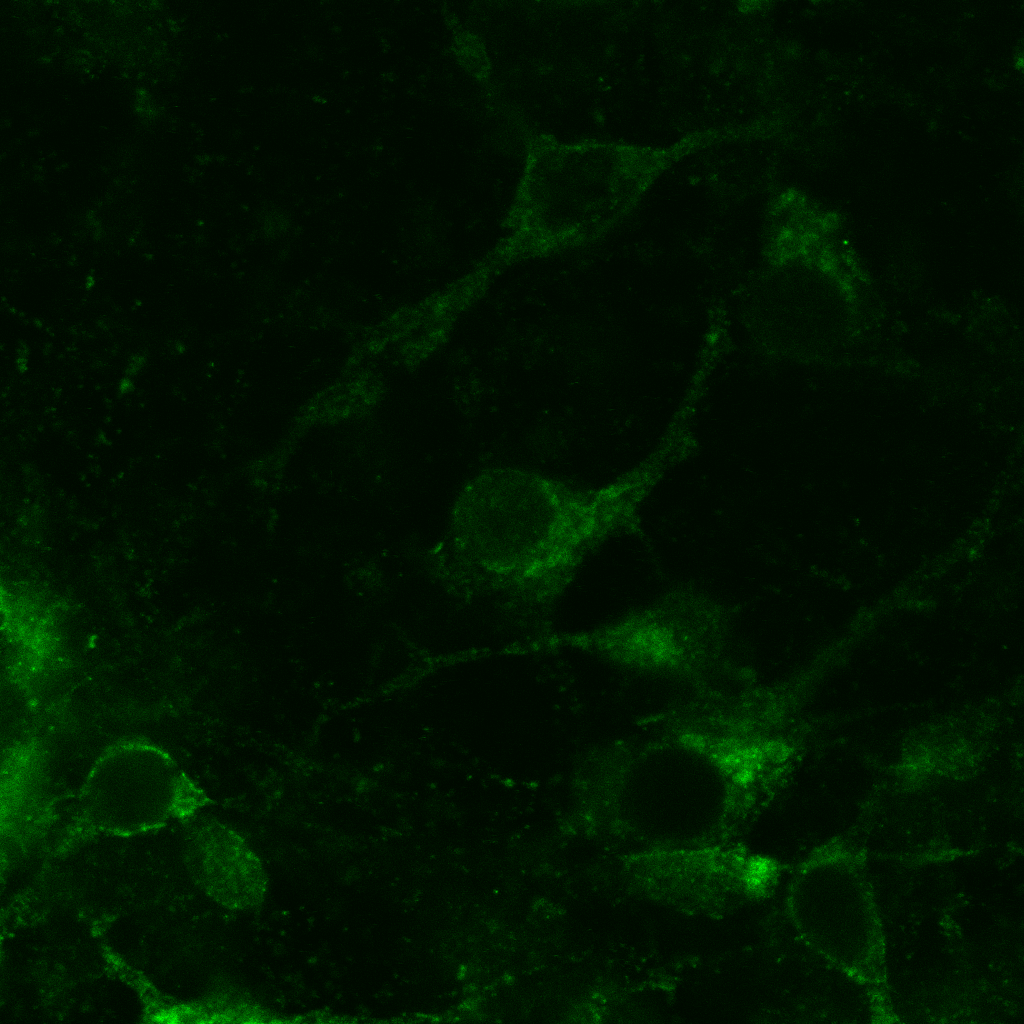

Supplement: Supplementary file 10 — Source Data for Figure 5 [file EMMM-15-e17101-s011.zip › Figure5/Image data-Fig5E/WT+Veh/Axin2.tif]

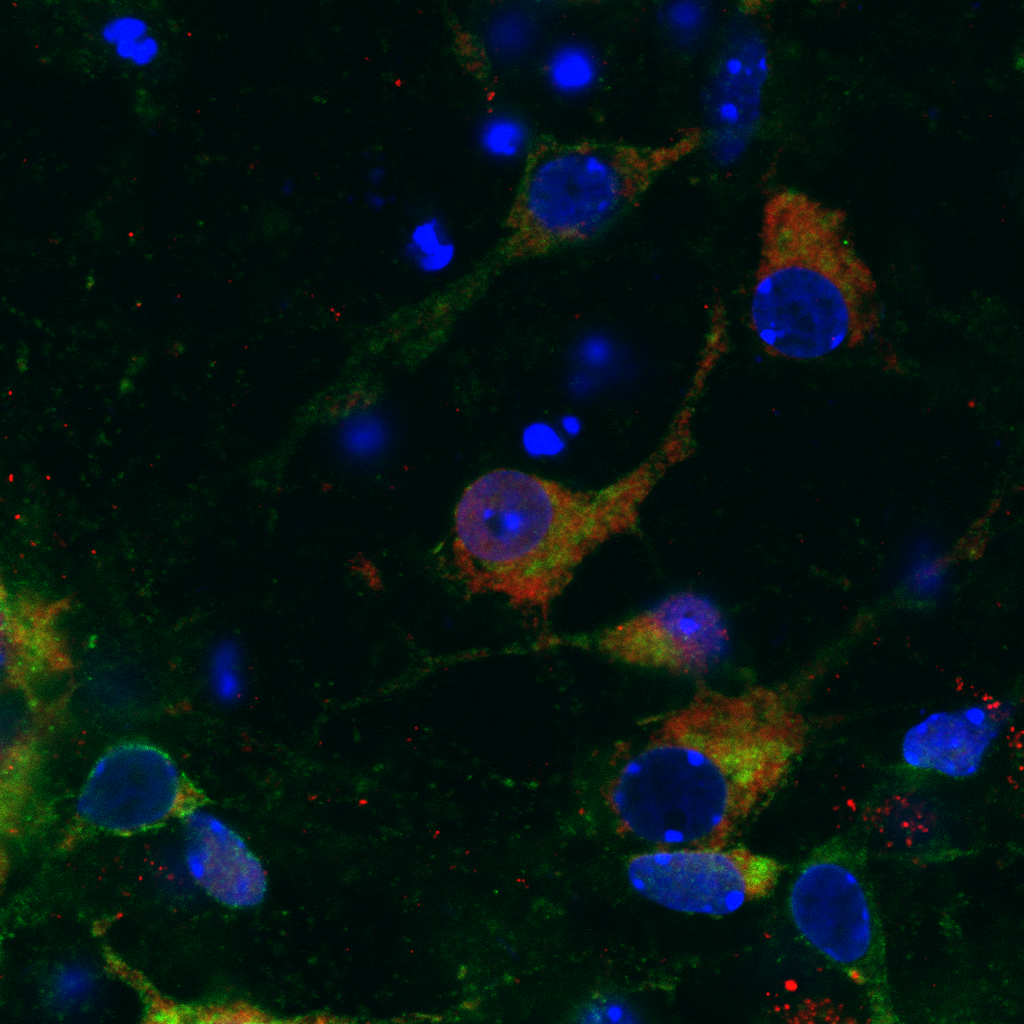

Supplement: Supplementary file 10 — Source Data for Figure 5 [file EMMM-15-e17101-s011.zip › Figure5/Image data-Fig5E/WT+Veh/Merge.tif]

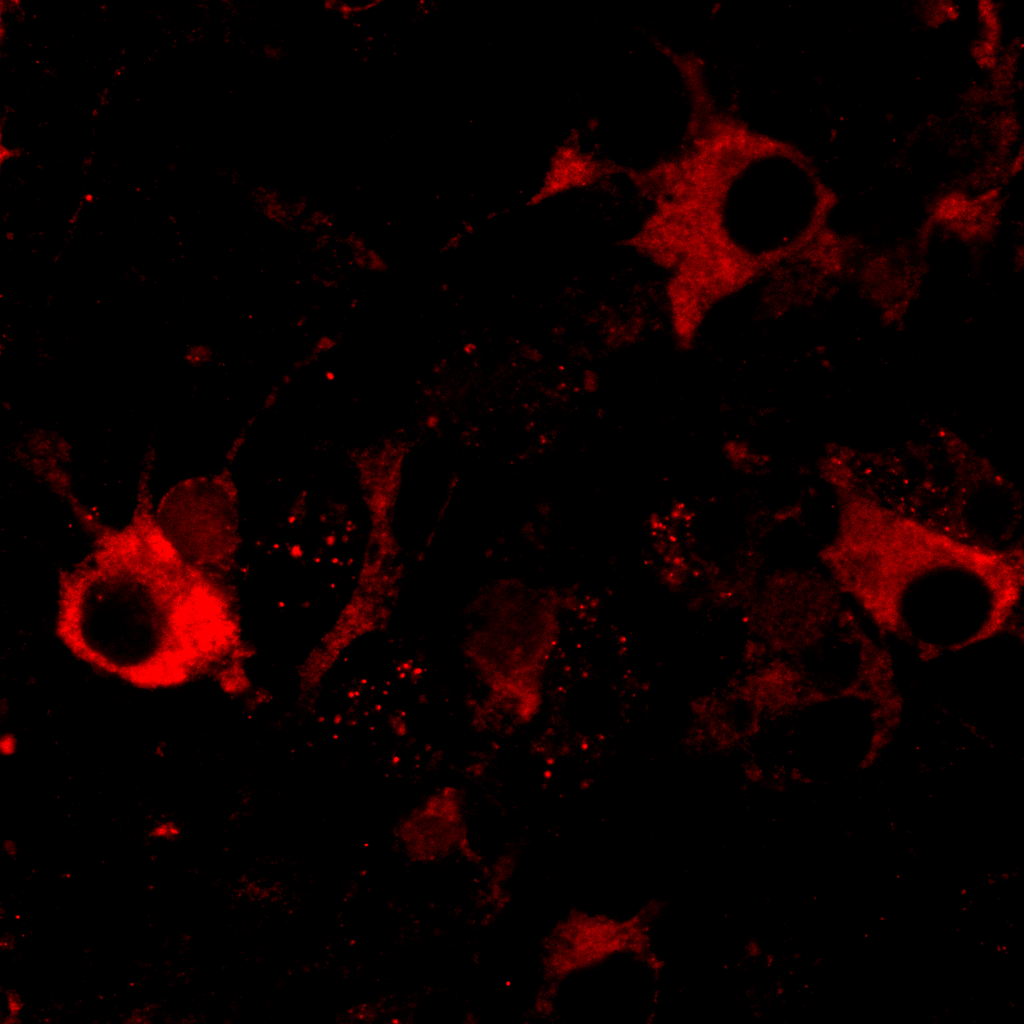

Supplement: Supplementary file 10 — Source Data for Figure 5 [file EMMM-15-e17101-s011.zip › Figure5/Image data-Fig5E/KO+Veh/ENO1.tif]

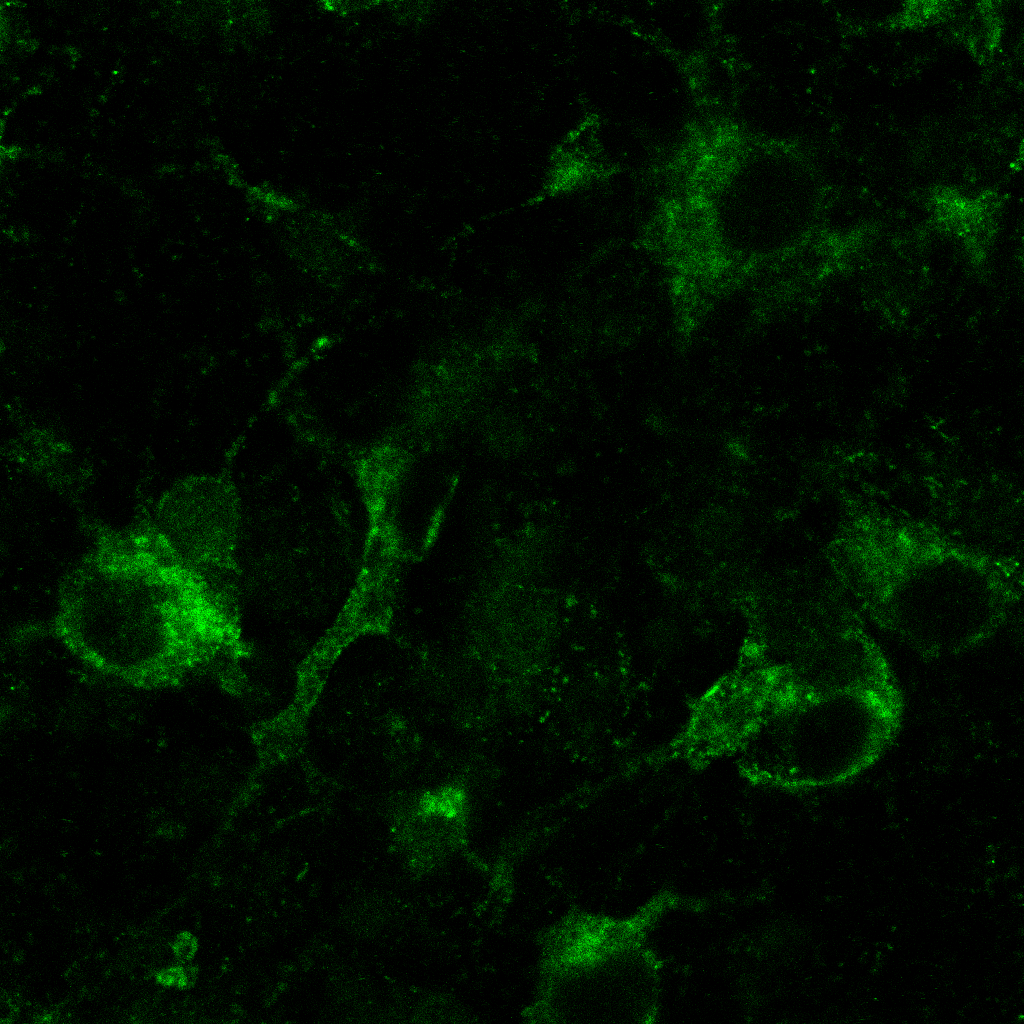

Supplement: Supplementary file 10 — Source Data for Figure 5 [file EMMM-15-e17101-s011.zip › Figure5/Image data-Fig5E/KO+Veh/Axin2.tif]

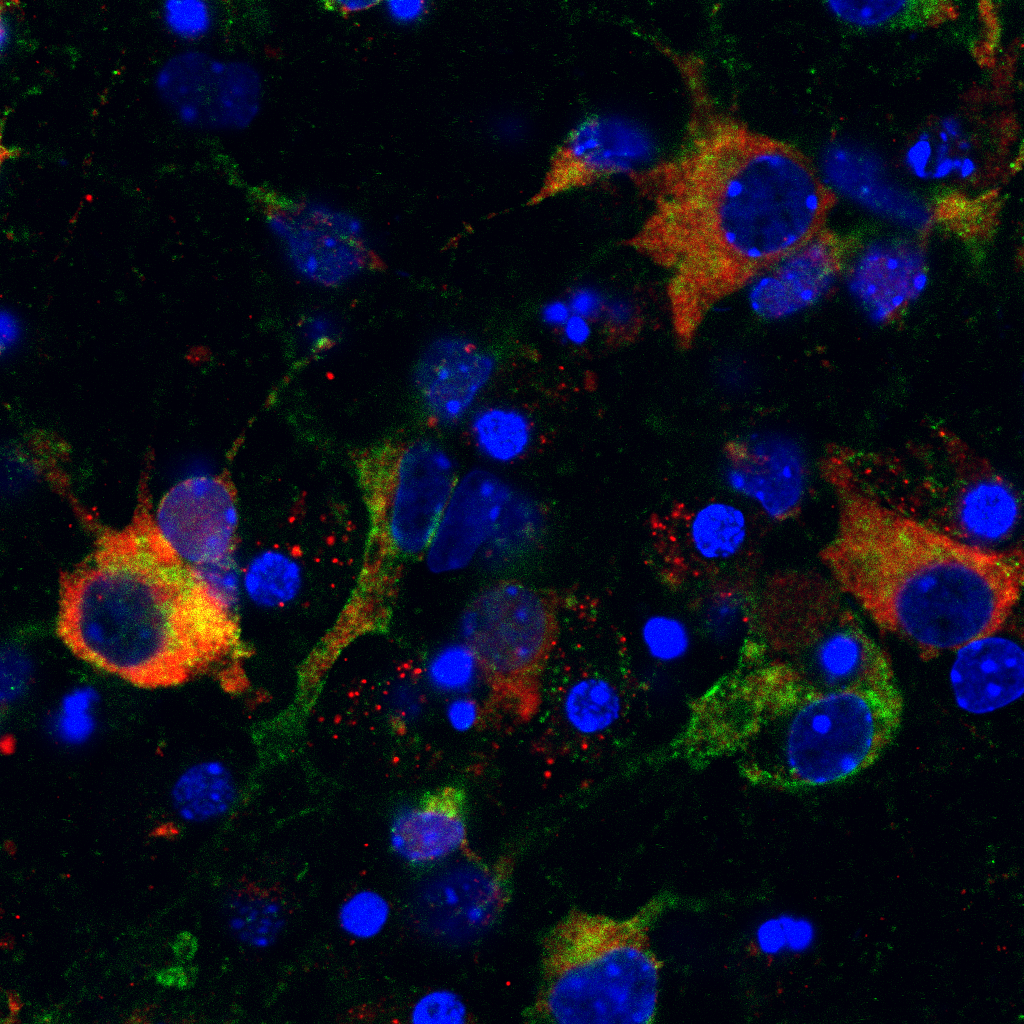

Supplement: Supplementary file 10 — Source Data for Figure 5 [file EMMM-15-e17101-s011.zip › Figure5/Image data-Fig5E/KO+Veh/Merge.tif]

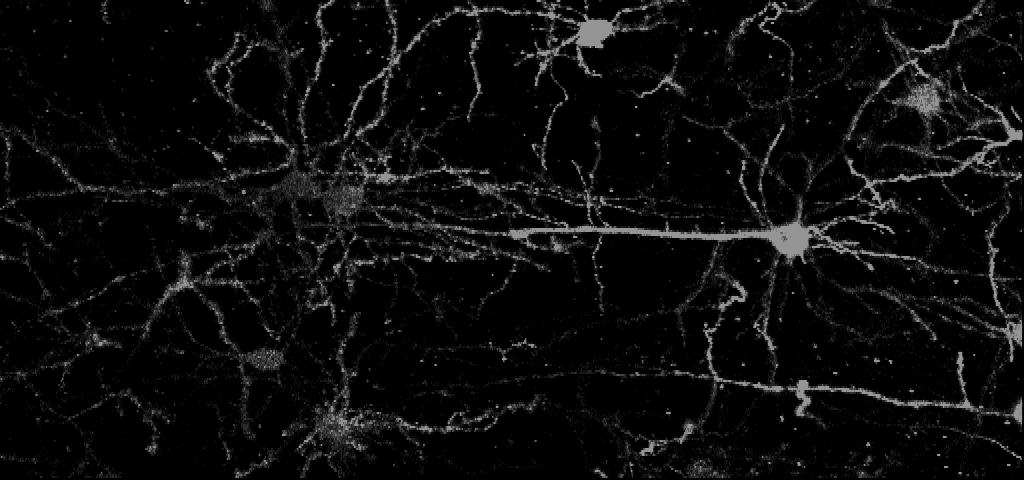

Supplement: Supplementary file 11 — Source Data for Figure 6 [file EMMM-15-e17101-s002.zip › Figure6/Image data-Fig6B/KO+XAV939/Typical image of neuron.tif]

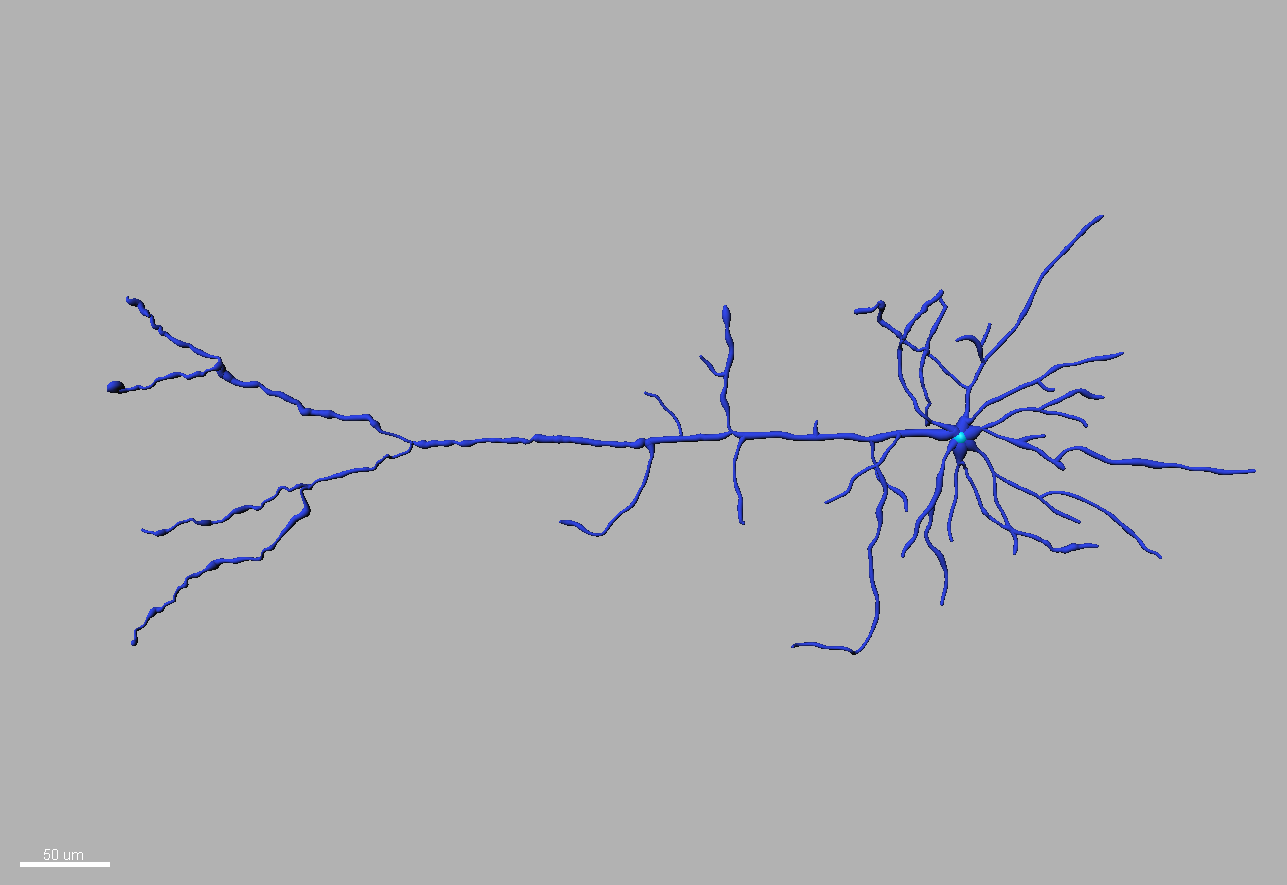

Supplement: Supplementary file 11 — Source Data for Figure 6 [file EMMM-15-e17101-s002.zip › Figure6/Image data-Fig6B/KO+XAV939/3D-reconstructed neuron.tif]

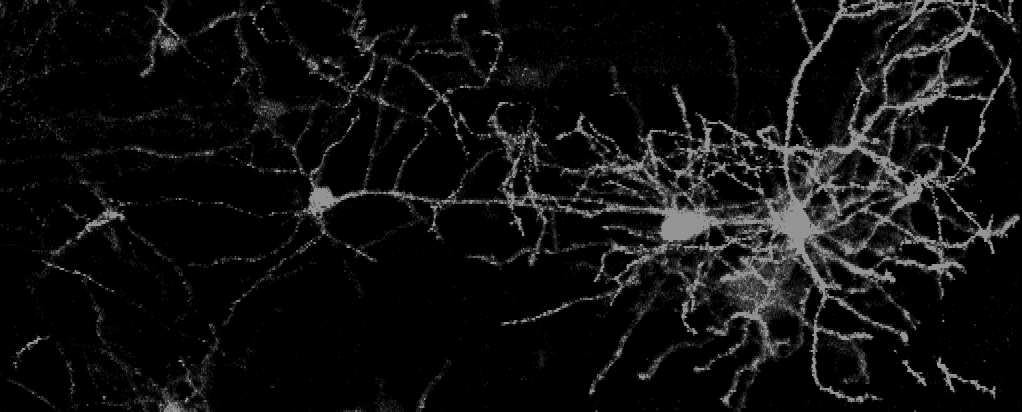

Supplement: Supplementary file 11 — Source Data for Figure 6 [file EMMM-15-e17101-s002.zip › Figure6/Image data-Fig6B/KO+Veh/Typical image of neuron.tif]

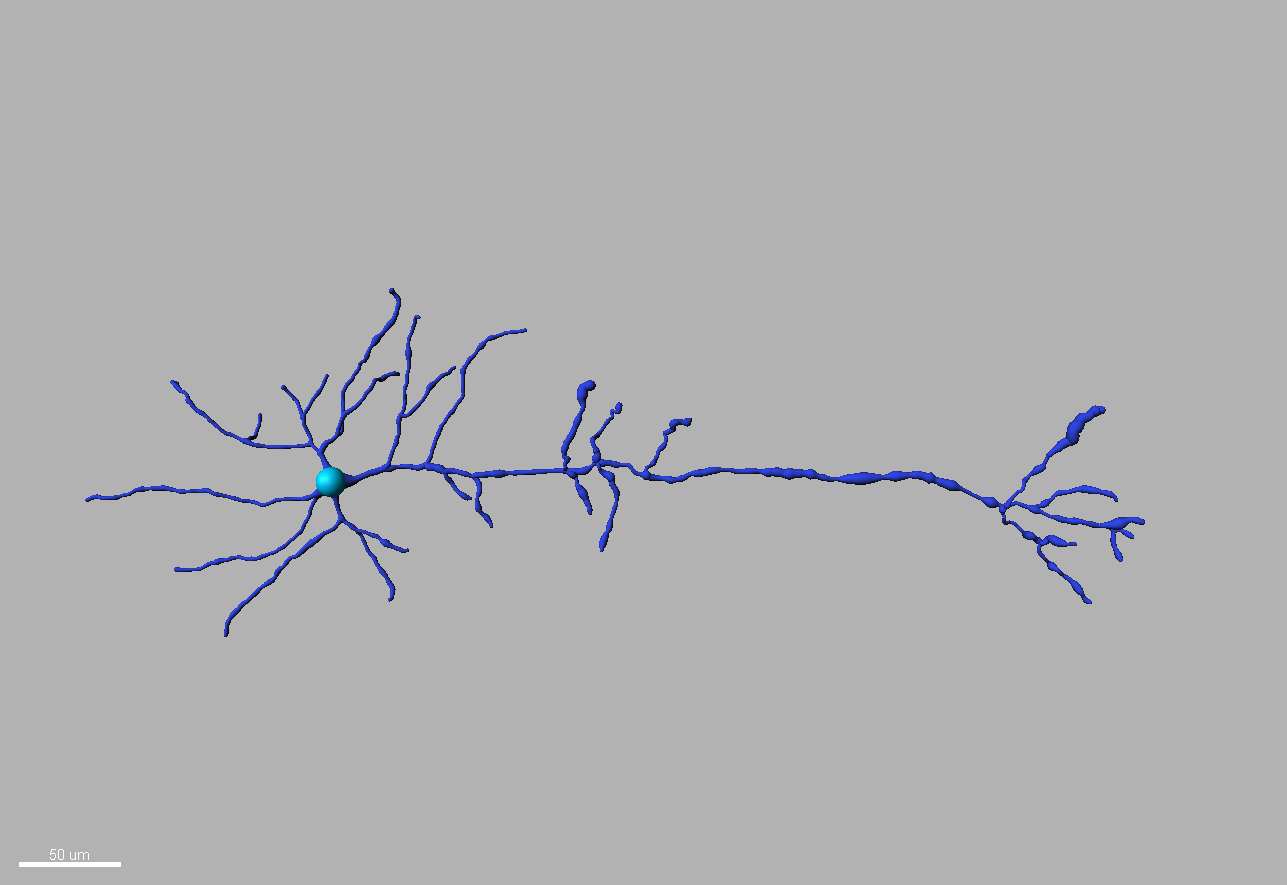

Supplement: Supplementary file 11 — Source Data for Figure 6 [file EMMM-15-e17101-s002.zip › Figure6/Image data-Fig6B/KO+Veh/3D-reconstructed neuron.tif]

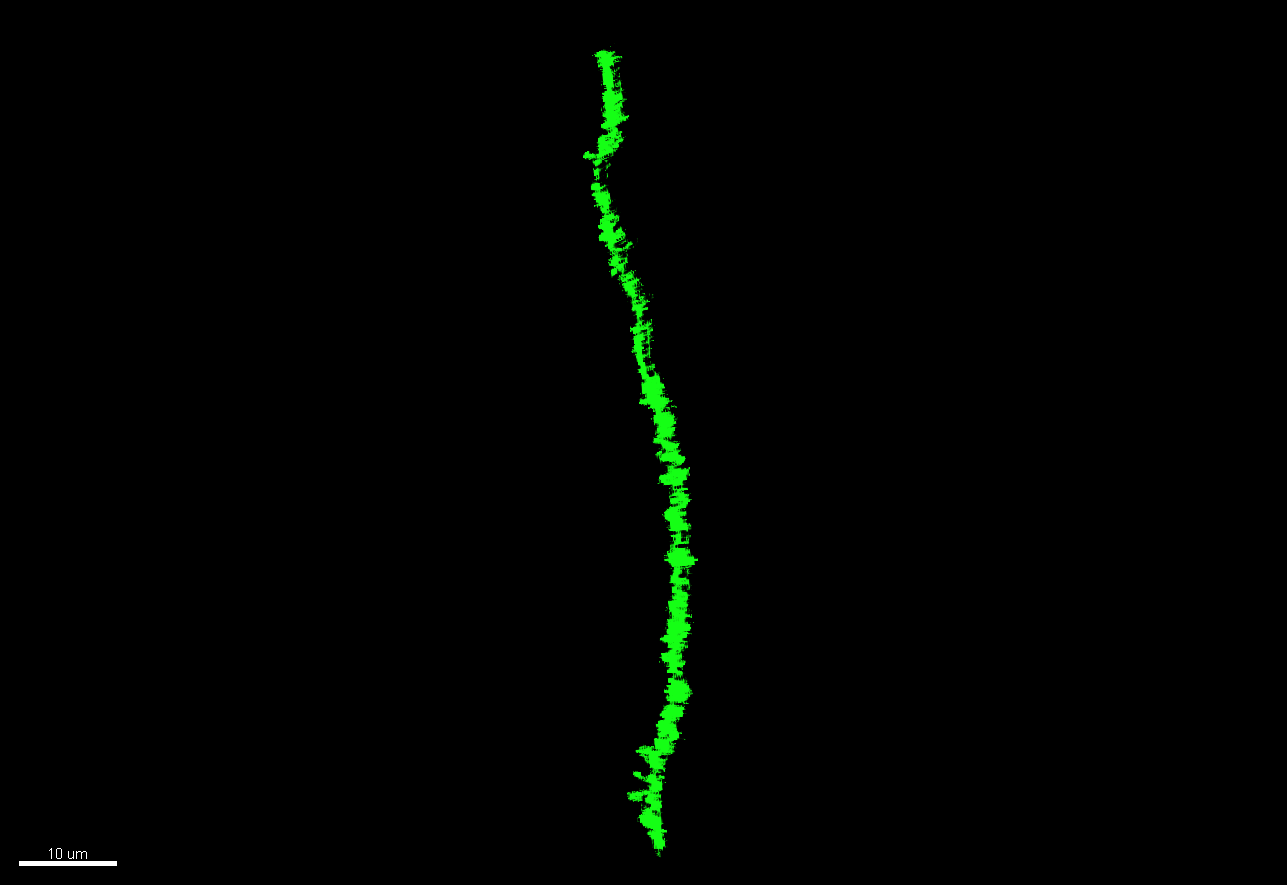

Supplement: Supplementary file 11 — Source Data for Figure 6 [file EMMM-15-e17101-s002.zip › Figure6/Image data-Fig6E/KO+XAV939/Typical image of spine.tif]

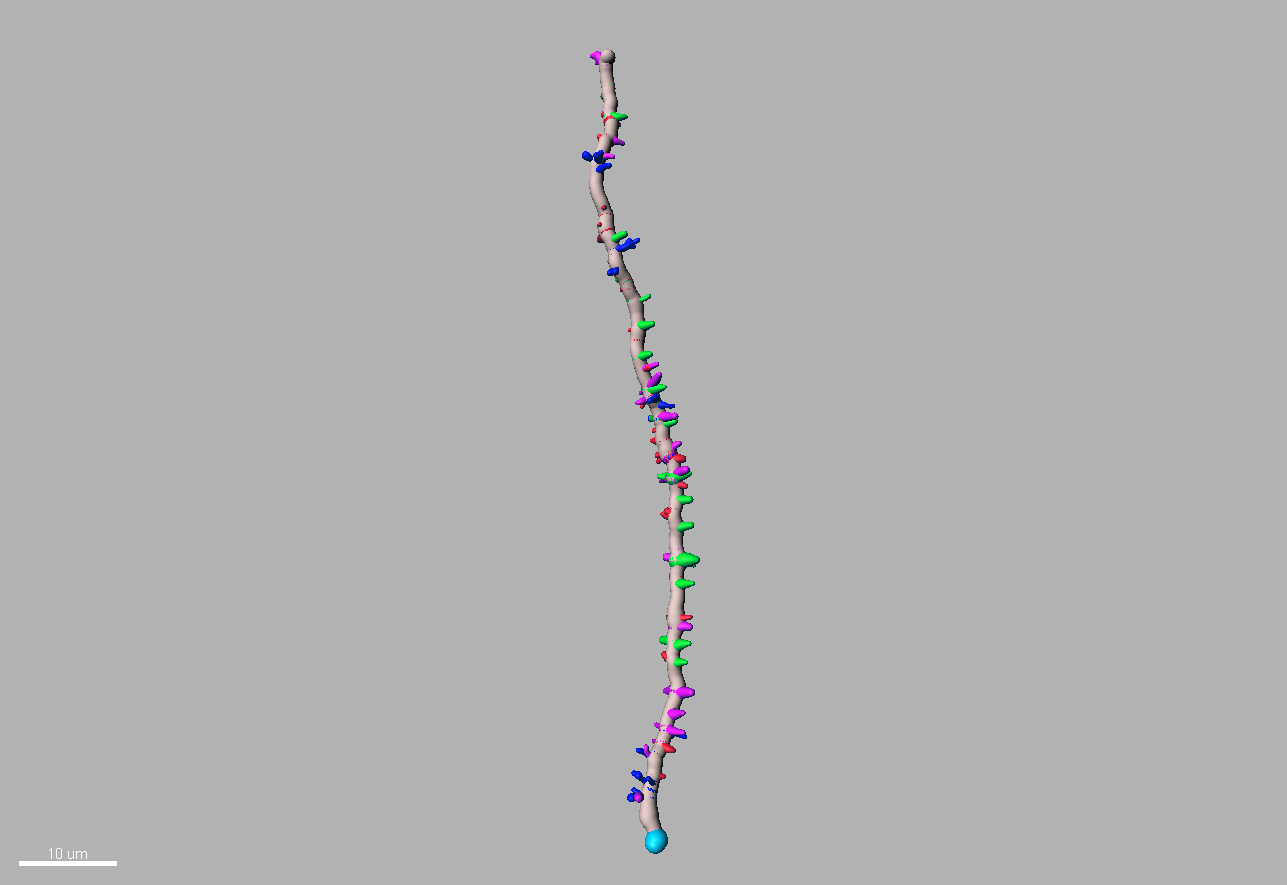

Supplement: Supplementary file 11 — Source Data for Figure 6 [file EMMM-15-e17101-s002.zip › Figure6/Image data-Fig6E/KO+XAV939/3D-reconstructed spine.tif]

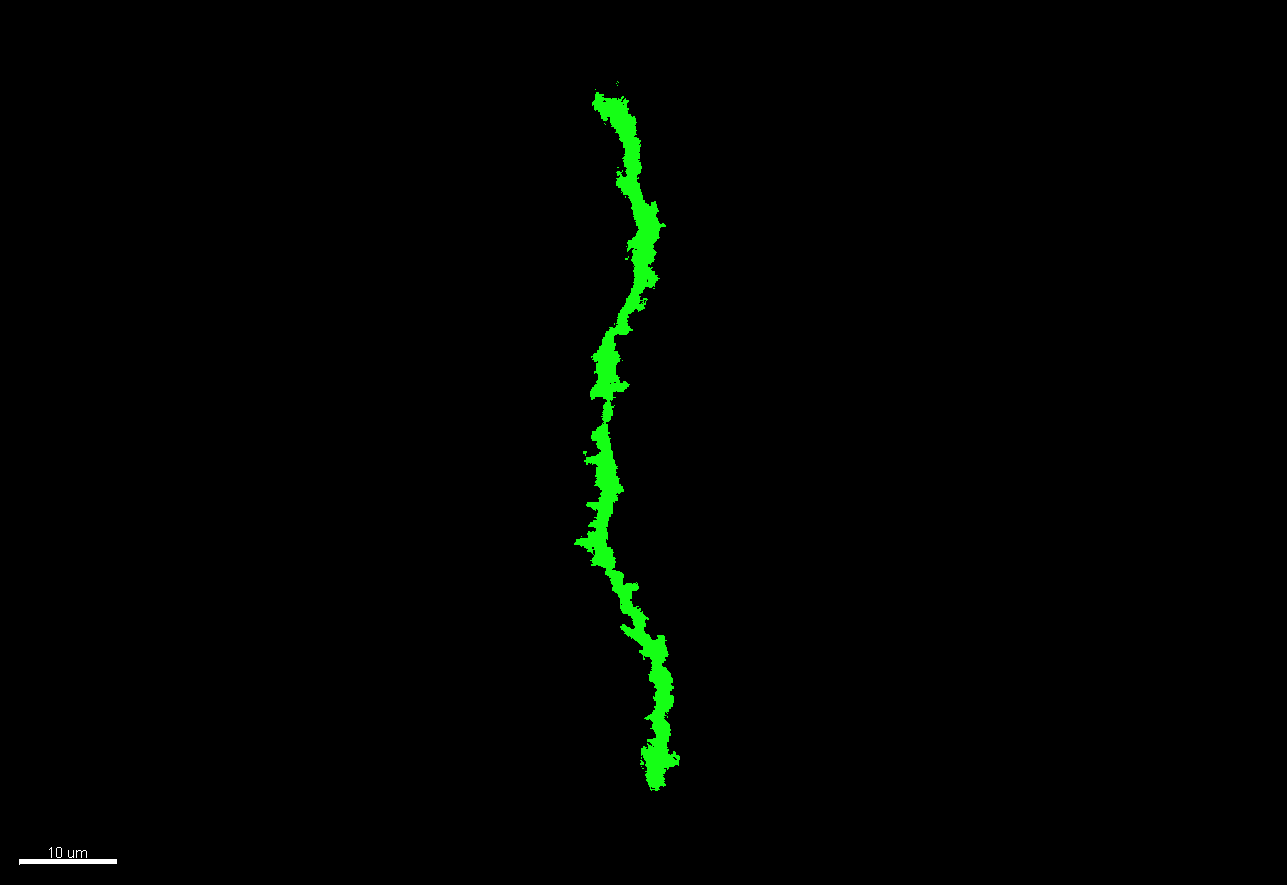

Supplement: Supplementary file 11 — Source Data for Figure 6 [file EMMM-15-e17101-s002.zip › Figure6/Image data-Fig6E/KO+Veh/Typical image of spine.tif]

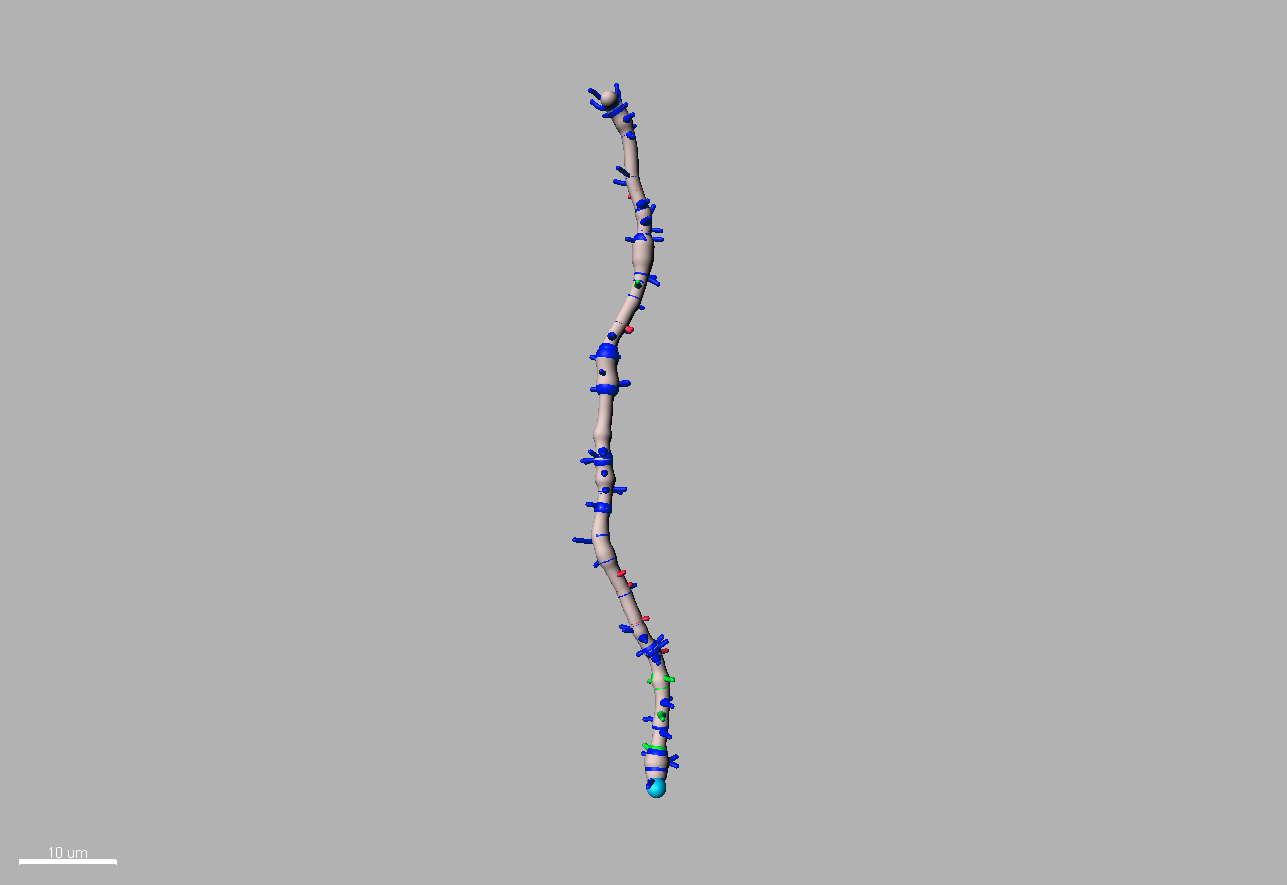

Supplement: Supplementary file 11 — Source Data for Figure 6 [file EMMM-15-e17101-s002.zip › Figure6/Image data-Fig6E/KO+Veh/3D-reconstructed spine.tif]
