# Supplementary material for: Axin2 coupled excessive Wnt‐glycolysis signaling mediates social defect in autism spectrum disorders
Source: EMBO Mol Med. 2023 Apr 20;15(6):e17101. doi: 10.15252/emmm.202217101 (PMC10245038; doi:10.15252/emmm.202217101)
Supplement: Supplementary file 14 [file EMMM-15-e17101-s007.pdf]

EMBO Molecular Medicine

Axin2 coupled excessive Wnt-glycolysis signaling mediates social defect in autism spectrum disorders

Mengmeng Wang, Panpan Xian, Weian Zheng, Zhenzhen Li, Andi Chen, Haoxiang Xiao, Chao Xu, Fei Wang, Honghui Mao, Han Meng, Youyi Zhao, Ceng Luo, Ya-Zhou Wang, and Shengxi Wu
DOI: 10.15252/emmm.202217101

Corresponding authors: Shengxi Wu (shengxi@fmmu.edu.cn) , Ya-Zhou Wang (yazhouw@fmmu.edu.cn)

Review Timeline:

Submission Date:	25th Oct 22
Editorial Decision:	30th Nov 22
Revision Received:	15th Feb 23
Editorial Decision:	23rd Mar 23
Revision Received:	30th Mar 23
Accepted:	3rd Apr 23

Editor: Jingyi Hou / Zeljko Durdevic

Transaction Report:

(Note: With the exception of the correction of typographical or spelling errors that could be a source of ambiguity, letters and reports are not edited. Depending on transfer agreements, referee reports obtained elsewhere may or may not be included in this compilation. Referee reports are anonymous unless the Referee chooses to sign their reports.)

30th Nov 2022

Dear Prof. Wu,

Thank you again for submitting your work to EMBO Molecular Medicine. We have now heard back from the three referees who agreed to evaluate your manuscript. As you will see from the reports below, the referees are overall supportive and think the study is interesting. Still, they raise a series of concerns, which we would ask you to address in a revision of the manuscript.

The referees' recommendations are relatively straightforward, so there is no need to reiterate their comments. All issues raised by the referees need to be satisfactorily addressed. Please feel free to contact me in case you would like to discuss in further detail any of the issues raised by the referees.

We would welcome the submission of a revised version within three months for further consideration. Please note that EMBO Molecular Medicine strongly supports a single round of revision. As acceptance or rejection of the manuscript will depend on another round of review, your responses should be as complete as possible.

EMBO Molecular Medicine has a "scooping protection" policy, whereby similar findings that are published by others during review or revision are not a criterion for rejection. Should you decide to submit a revised version, I do ask that you get in touch after three months if you have not completed it to update us on the status.

We are aware that many laboratories cannot function at full efficiency during the current COVID-19/SARS-CoV-2 pandemic and have therefore extended our "scooping protection policy" to cover the period required for a full revision to address the experimental issues. Please let me know should you need additional time, and also if you see a paper with related content published elsewhere.

Please read below for important editorial formatting and consult our author's guidelines for proper formatting of your revised article for EMBO Molecular Medicine.

I look forward to receiving your revised manuscript soon.

Sincerely,
Jingyi

Jingyi Hou
Editor
EMBO Molecular Medicine

*****

When submitting your revised manuscript, please carefully review the instructions that follow below. We perform an initial quality control of all revised manuscripts before re-review; failure to include requested items will delay the evaluation of your revision.

We require:

1) A .docx formatted version of the manuscript text (including legends for main figures, EV figures and tables). Please make sure that the changes are highlighted to be clearly visible.

2) Individual production quality figure files as .eps, .tif, .jpg (one file per figure). For guidance, download the 'Figure Guide PDF': (https://www.embopress.org/page/journal/17574684/authorguide#figureformat).

3) A .docx formatted letter INCLUDING the reviewers' reports and your detailed point-by-point responses to their comments. As part of the EMBO Press transparent editorial process, the point-by-point response is part of the Review Process File (RPF), which will be published alongside your paper.

4) A complete author checklist, which you can download from our author guidelines (https://www.embopress.org/page/journal/17574684/authorguide#submissionofrevisions). Please insert information in the

checklist that is also reflected in the manuscript. The completed author checklist will also be part of the RPF.

5) Please note that all corresponding authors are required to supply an ORCID ID for their name upon submission of a revised manuscript.

6) It is mandatory to include a 'Data Availability' section after the Materials and Methods. Before submitting your revision, primary datasets produced in this study need to be deposited in an appropriate public database, and the accession numbers and database listed under 'Data Availability'. Please remember to provide a reviewer password if the datasets are not yet public (see https://www.embopress.org/page/journal/17574684/authorguide#dataavailability).

In case you have no data that requires deposition in a public database, please state so in this section. Note that the Data Availability Section is restricted to new primary data that are part of this study.

7) For data quantification: please specify the name of the statistical test used to generate error bars and P values, the number (n) of independent experiments (specify technical or biological replicates) underlying each data point and the test used to calculate p-values in each figure legend. The figure legends should contain a basic description of n, P and the test applied. Graphs must include a description of the bars and the error bars (s.d., s.e.m.). See also 'Figure Legend' guidelines: https://www.embopress.org/page/journal/17574684/authorguide#figureformat

8) At EMBO Press we ask authors to provide source data for the main manuscript figures. Our source data coordinator will contact you to discuss which figure panels we would need source data for and will also provide you with helpful tips on how to upload and organize the files.

9) Our journal encourages inclusion of *data citations in the reference list* to directly cite datasets that were re-used and obtained from public databases. Data citations in the article text are distinct from normal bibliographical citations and should directly link to the database records from which the data can be accessed. In the main text, data citations are formatted as follows: "Data ref: Smith et al, 2001" or "Data ref: NCBI Sequence Read Archive PRJNA342805, 2017". In the Reference list, data citations must be labeled with "[DATASET]". A data reference must provide the database name, accession number/identifiers and a resolvable link to the landing page from which the data can be accessed at the end of the reference. Further instructions are available at .

10) We replaced Supplementary Information with Expanded View (EV) Figures and Tables that are collapsible/expandable online. A maximum of 5 EV Figures can be typeset. EV Figures should be cited as 'Figure EV1, Figure EV2" etc... in the text and their respective legends should be included in the main text after the legends of regular figures.

- For the figures that you do NOT wish to display as Expanded View figures, they should be bundled together with their legends in a single PDF file called *Appendix*, which should start with a short Table of Content. Appendix figures should be referred to in the main text as: "Appendix Figure S1, Appendix Figure S2" etc.

- Additional Tables/Datasets should be labeled and referred to as Table EV1, Dataset EV1, etc. Legends have to be provided in a separate tab in case of .xls files. Alternatively, the legend can be supplied as a separate text file (README) and zipped together with the Table/Dataset file.

See detailed instructions here:

.

11) The paper explained: EMBO Molecular Medicine articles are accompanied by a summary of the articles to emphasize the major findings in the paper and their medical implications for the non-specialist reader. Please provide a draft summary of your article highlighting

- the medical issue you are addressing,

- the results obtained and

- their clinical impact.

This may be edited to ensure that readers understand the significance and context of the research. Please refer to any of our published articles for an example.

12) For more information: There is space at the end of each article to list relevant web links for further consultation by our readers. Could you identify some relevant ones and provide such information as well? Some examples are patient associations, relevant databases, OMIM/proteins/genes links, author's websites, etc...

13) Author contributions: You will be asked to provide CRediT (Contributor Role Taxonomy) terms in the submission system. These replace a narrative author contribution section in the manuscript.

14) A Conflict of Interest statement should be provided in the main text.

15) Every published paper now includes a 'Synopsis' to further enhance discoverability. Synopses are displayed on the journal webpage and are freely accessible to all readers. They include a short stand first (maximum of 300 characters, including space) as well as 2-5 one-sentences bullet points that summarizes the paper. Please write the bullet points to summarize the key NEW findings. They should be designed to be complementary to the abstract - i.e. not repeat the same text. We encourage inclusion of key acronyms and quantitative information (maximum of 30 words / bullet point). Please use the passive voice. Please attach these in a separate file or send them by email, we will incorporate them accordingly.

Please also suggest a striking image or visual abstract to illustrate your article as a PNG file 550 px wide x 300-600 px high.

EMBO Molecular Medicine has a "scooping protection" policy, whereby similar findings that are published by others during review or revision are not a criterion for rejection. Should you decide to submit a revised version, I do ask that you get in touch after three months if you have not completed it, to update us on the status.

Please note: When submitting your revision you will be prompted to enter your funding and payment information. This will allow Wiley to send you a quote for the article processing charge (APC) in case of acceptance. This quote takes into account any reduction or fee waivers that you may be eligible for. Authors do not need to pay any fees before their manuscript is accepted and transferred to the publisher.

EMBO Press participates in many Publish and Read agreements that allow authors to publish Open Access with reduced/no publication charges. Check your eligibility: https://authorservices.wiley.com/author-resources/Journal-Authors/open-access/affiliation-policies-payments/index.html

***** Reviewer's comments *****

Referee #1 (Comments on Novelty/Model System for Author):

authors used genetically modified mice and human ES cell lines to address the role of glycosis in ASD-related mutations. results include both in vitro and in vivo data, which is quite convincing.

Referee #1 (Remarks for Author):

authors used Shank3 deletion mouse as ASD mouse model to find that glycolysis process is upregulated in Shank3 mouse. Authors then showed that suppressing glycolysis using multiple approaches could rescue social defects of Shank3 mutant mice. Interestingly, authors used Shank3 deleted human ES-derived neuron to show that manipulation glycolysis could also rescue the developmental defects in shank3 mutant human neurons. This work provides a crucial link to connect glycolysis in the brain with regulation of social behaviors. i have a few minor comments as following,
1. authors used 2-DG as a method to inhibit glycolysis. Although 2-DG is a glucose mimic which inhibits glycolysis, but massive application 2-DG into the brain may lead to some unknown side effects. So other than social behaviors, does 2-DG cause any other effects in the mice?
2. Above concern also applies for XAV939, although authors performed IV injection, does XAV939 lead to any other side effects in mice brain? Authors may look further to these pharmacological manipulations.

Referee #2 (Comments on Novelty/Model System for Author):

These two ASD mouse models are generally used to dissect pathological mechanisms underlying ASD.

Referee #2 (Remarks for Author):

The study by Wang et al. sought to identify convergent molecular mechanism underlying ASD associated social dysfunction. By using two ASD mouse models, the authors found that aberrant activation of Wnt-glycolysis signaling in the ACC mediates both social exploration and social novelty defects. They further identified axin2 as a key molecule linking Wnt over-activation and glycolysis elevation in ASD mice. Overall the authors reported several findings that are important to understand ASD pathology and the molecules revealed in this study could potentially serve as therapeutic targets. While the findings are interesting, there are some major issues to be addressed to improve the quality of the study.

1. When does the Wnt signaling over-activation or glycolysis elevation start to happen? At embryonic stage, early postnatal

days, or adulthood? Does it evolve over time? Correspondingly, is there a difference in terms of rescue effect in behavioral outcomes when intervention conducted at different time points? Answers to these questions are required to better understand the pathological mechanism of ASD and to develop therapeutic strategy.

2. Social function is accomplished by extremely complicated neural network. Except for ACC, there are other major brain regions involved, such as VTA and NAc. Is the aberrant signaling pathway revealed in the present study specific for ACC? Or alternatively, is it generally true for other social-relevant brain regions?

3. ASD is a spectrum disorder characterized by social deficits and repetitive behavior. Although the present study investigated the social domain, it is equally important to know whether the Wnt-glycolysis signaling pathway is also involved in other ASD behavioral phenotypes, such as repetitive behavior.

4. The ages of animals used for each experiment were not indicated in the manuscript. This information is necessary to understand the context of individual experiments and should be clearly described accordingly.

5. This study employed two ASD mouse models to identify convergent molecular mechanism underlying ASD. There is a big difference in axin2/tubulin ratio (Figure 1F vs Figure 1I), ATP level (Figure 2A), and lactate level (Figure 2E vs Figure 2G) between wild type mice of each model. Why is that?

Referee #3 (Comments on Novelty/Model System for Author):

Neurologic disorders with social dysfunction are a prominent part of developmental disorders. The WNT signaling system may be linked to 30-40% of ASD and metabolic abnormalities are similarly seen in at least 30% of ASD patients. Thus, this investigation and its findings fills an important gap in the literature. It is further bolstered by findings related to mTOR signaling in autism. As such this manuscript is an important contribution to the autism literature.

All papers have some areas of improvement and here are the reviewer's suggestions:
1) In the Introduction, the last paragraphs should introduce the central hypothesis of the paper and then major aims or questions to be asked by the investigation.
2) Under the section: "Wnt signaling over-activation in ACC impairs social function"--please re-word the description of Fig 3 to indicate plainly whether social preference is present or not and whether social novelty is present or not. I think I understand but it is worded unclearly and needs clarification.
3) Do we know why compound XAV939 has no effect in WT mice?
4) While we know the length/age of neurons in mice, it is unclear whether the human neuron experiments replicate that which is seen in vivo and therefore whether Axin2 is a reasonable drug target. The developmental time course of Axin2 interactions in vitro was not studied or at least presented in the manuscript. The authors did show they replicate the mouse data, but when, where, and how they came to this data is unclear. XAV939 could be significantly more toxic at certain ages and that is important to know.
5) Many genetic factors are prenatal and second trimester events in humans. Although postnatal Axin2 targeting could help ASD, the real question is what happens if you do a prenatal targeting of the Axin2 interactions? What happens?
6) The authors suggest that nuclear vs cytosolic targeting of beta-catenin signaling may be important? is there other examples to back up this observation??
7) In Fig S4, please replace the Western blot of Shank 3. The reviewer cannot tell anything from this blot.

The reviewer congratulates the authors on a wonderful study and its potential clinical implications. The paper just needs some edits to improve its impact.

Referee #3 (Remarks for Author):

Great study. Should be published after addressing major critiques.

Point-by-point response letter

We thank the reviewers for their very valuable and constructive comments. The manuscript is now revised according to their suggestions. The followings are our point-by-point reply to all reviewers' comments.

Referee #1

authors used Shank3 deletion mouse as ASD mouse model to find that glycolysis process is upregulated in Shank3 mouse. Authors then showed that suppressing glycolysis using multiple approaches could rescue social defects of Shank3 mutant mice. Interestingly, authors used Shank3 deleted human ES-derived neuron to show that manipulation glycolysis could also rescue the developmental defects in shank3 mutant human neurons. This work provides a crucial link to connect glycolysis in the brain with regulation of social behaviors. i have a few minor comments as following,

1. authors used 2-DG as a method to inhibit glycolysis. Although 2-DG is a glucose mimic which inhibits glycolysis, but massive application 2-DG into the brain may lead to some unknown side effects. So other than social behaviors, does 2-DG cause any other effects in the mice?

Reply:
We agree that massive application of 2-DG may initiate other effects in brain. Under normal condition, neurons prefer oxidative phosphorylation for energy supply and only mobilize glycolysis in conditions of hyperactivity (Physiol Rev. 2019 Jan 1;99(1):949-1045.). 2-DG has been used to suppress the hyperactivity of excitatory neurons and to reduce epileptic behaviors (JCI Insight, 2019 Apr 30;5(11):e126506. Ann Neurol, 2009 Apr;65(4):435-47.), which is in some way beneficial for ASD treatment because epilepsy is a concomitant disease of ASD. In our study, aside from social behaviors, we assessed the effects of 2-DG on repetitive and anxious behaviors. Shank3-KO mice treated with or without 2-DG showed similar behaviors in grooming, open-field and elevated plus maze tests, suggesting that 2-DG treatment had no significant effects on the repetitive and anxious behaviors of ASD mice. New data have been included in Fig. EV3F-H.

2. Above concern also applies for XAV939, although authors performed IV injection, does XAV939 lead to any other side effects in mice brain? Authors may look further to these pharmacological manipulations.

Reply:
Thank you for the question. As XAV939 can't be applied systematically, we injected it directly into ACC. Therefore, we assessed its effects on ASD associated behaviors (repetitive stereotype behavior, anxious behaviors, and social behaviors). Shank3-KO mice injected with or without XAV939 showed similar behaviors in grooming, open-field and elevated plus maze tests, suggesting that ACC delivery of XAV939 had no significant effects on the repetitive and anxious behaviors of ASD mice. As over-activation of Wnt signaling occurs mainly in ACC and starts from P14 in ASD mice, and there is no or very low levels of Wnt activity in most regions of postnatal brain (except for few brain regions such as hippocampus) in wild type mice, we think that it would be relatively safe for using XAV939 in adult. New data of behavior tests and developmental Wnt alteration have been included in Fig. EV2A-F, and Fig. EV4F-H.

Referee #2 (Comments on Novelty/Model System for Author):

These two ASD mouse models are generally used to dissect pathological mechanisms underlying ASD.

Referee #2 (Remarks for Author):

The study by Wang et al. sought to identify convergent molecular mechanism underlying ASD associated social dysfunction. By using two ASD mouse models, the authors found that aberrant activation of Wnt-glycolysis signaling in the ACC mediates both social exploration and social novelty defects. They further identified axin2 as a key molecule linking Wnt over-activation and glycolysis elevation in ASD mice. Overall the authors reported several findings that are important to understand ASD pathology and the molecules revealed in this study could potentially serve as therapeutic targets. While the findings are interesting, there are some major issues to be addressed to improve the quality of the study.

1. When does the Wnt signaling over-activation or glycolysis elevation start to happen? At embryonic stage, early postnatal days, or adulthood? Does it evolve over time? Correspondingly, is there a difference in terms of rescue effect in behavioral outcomes when intervention conducted at different time points? Answers to these questions are required to better understand the pathological mechanism of ASD and to develop therapeutic strategy.

Reply:

Thank you for the questions. If we understand correctly, these questions can be grouped into two. We reply them one-by-one.

(1) When does the Wnt signaling over-activation or glycolysis elevation start to happen? At embryonic stage, early postnatal days, or adulthood? Does it evolve over time?

We examined the expression of key Wnt signaling components (p-GSK3β, p-β-catenin and TCFL1) and measured the levels of lactic acid and pyruvic acid in the ACC of Shank3-KO and VPA-treated mice at different developmental time points. Our data showed that Wnt signaling and lactate increased from P14 in both ASD models. Pyruvic acid decreased in Shank3-KO mice from P21 while increased in VPA-treated mice from P14. In general, these data suggested that both Wnt signaling and glycolysis were up-regulated in the ACC of Shank3-KO and VPA-treated mice from juvenile and evolved to adult. New data have been included in Fig. EV2A-F and Fig. EV2H-K.

(2) Correspondingly, is there a difference in terms of rescue effect in behavioral outcomes when intervention conducted at different time points?

The above data indicated that inhibiting Wnt signaling or glycolysis at the turning point of Wnt/glycolysis alteration might be better for preventing ASD development. As XAV939 can't be used systematically, we had tried to deliver it to ACC from 2 weeks after birth. However, as the skull grows quickly during this period, it was very hard to fix the microinjection guide cannulas and we failed to conduct consecutive microinjection. In regards of glycolysis, we adopted two experimental paradigms: (1) i.p. injection starting from P10 (3 consecutive days) to evaluate ultrasonic vocalization at P14; (2) i.p. injection starting from P7 (7 consecutive days) to evaluate social function in adult. In first experimental paradigm, we observed significant improvement of ultrasonic vocalization in both ASD models (See the below). In second experimental paradigm, most mice died soon after 2-DG treatment. These data indicated that early systematic suppression of glycolysis shall be carefully considered. Short-term application maybe beneficial for social communication while long-term application may be toxic to mice development. Therefore, it would be optimal to inhibit Wnt/glycolysis neuron-specifically or brain-specifically. This will need large amount experiments to investigate. We wish to report our detailed study on this issue in the future and thus non including these data in our revised manuscript.

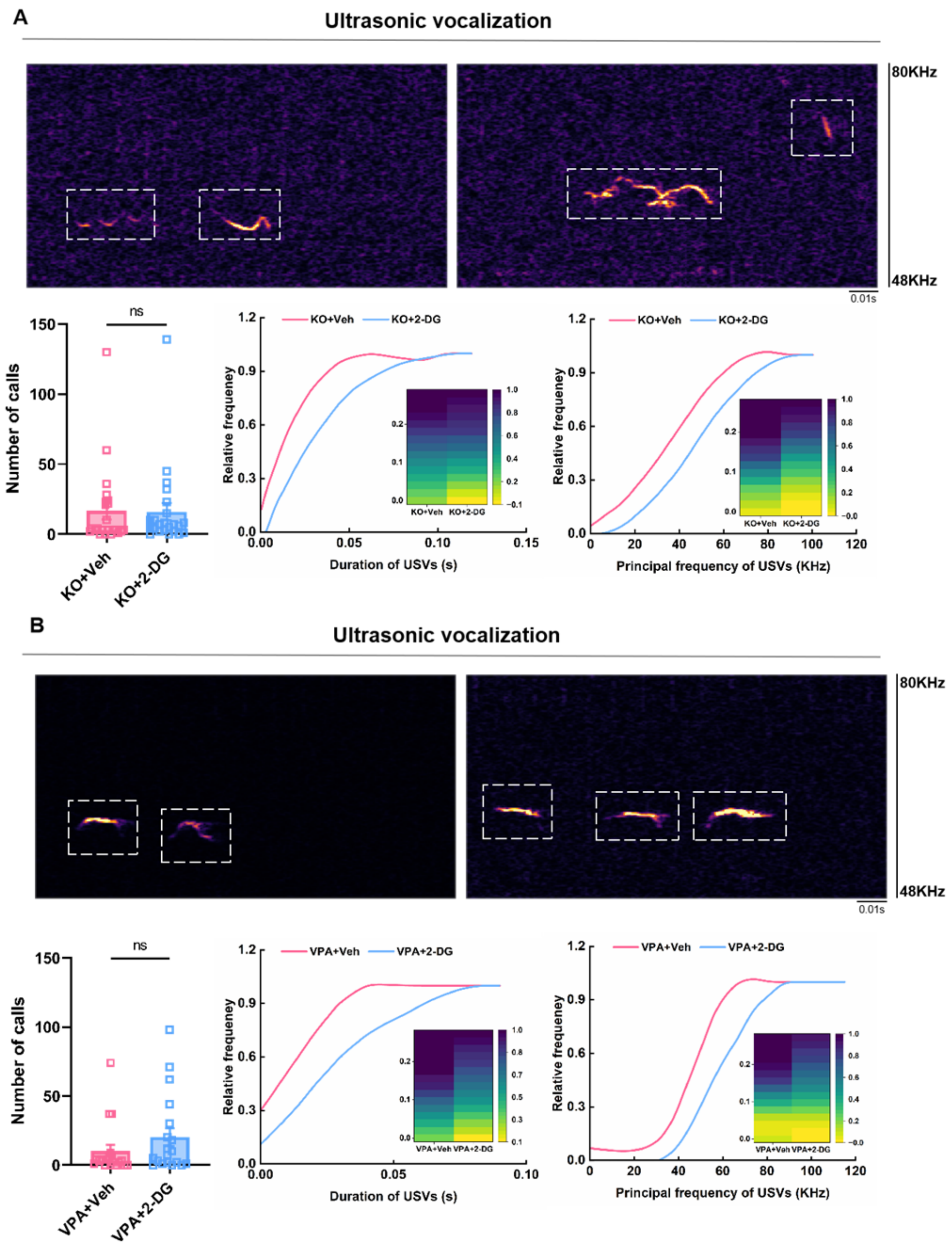

(A) Effects of 2-DG treatment on ultrasonic vocalization of Shank3$^{-/-}$ mice. 2-DG treatment significantly increased the average frequency and duration of individual ultrasonic vocalizations of Shank3$^{-/-}$ mice. (B) Effects of 2-DG treatment on ultrasonic vocalization of VPA-treated mice. Similarly, 2-DG treatment significantly increased the average frequency and duration of ultrasonic vocalization of VPA-pretreated mice.

2. Social function is accomplished by extremely complicated neural network. Except for ACC, there are other major brain regions involved, such as mPFC, VTA and NAc.

Is the aberrant signaling pathway revealed in the present study specific for ACC? Or alternatively, is it generally true for other social-relevant brain regions?

Reply:

Thank you for the question. We examined the expression of Axin2 and p-β-catenin (S33, an inhibitory form of β-catenin) in the mPFC, VTA and NAc of Shank3-KO and VPA-treated mice as you suggested. Shank3-KO mice showed significant increase of Axin2 and decrease of p-β-catenin(S33) in mPFC. VPA-treated mice showed significant decrease of p-β-catenin(S33) but no changes of Axin2 in mPFC. Both mice showed no changes of these two proteins in VTA and NAc. The changes of β-catenin in mPFC were consistent with previous report that nuclear β-catenin is up-regulated in the mPFC of Shank3-KO mice (Nat Neurosci. 2018 Apr;21(4):564-575). These data indicated that the alteration of Wnt signaling is brain-region dependent. This is understandable as Shank3-KO mice exhibits different phenotypes in different brain regions. These data, in together with those presented in Figure-1, indicated that ACC was the major brain region where Wnt signaling was aberrant. New data has been included in Fig. EV1A-F.

3. ASD is a spectrum disorder characterized by social deficits and repetitive behavior. Although the present study investigated the social domain, it is equally important to know whether the Wnt-glycolysis signaling pathway is also involved in other ASD behavioral phenotypes, such as repetitive behavior.

Reply:

To address this question, we evaluated the expression of β-catenin and Axin2, and measured the levels of lactic acid and pyruvic acid in the striatum (the key brain region for repetitive behavior) of wild type and Shank3-KO mice. The results showed no changes of these proteins and molecules in the striatum of Shank3-KO mice (Fig. EV1G, Fig. EV2G). Further, no difference of grooming was found between Shank3-KO mice treated with or without 2-DG (Fig. EV3F) In addition, no changes of grooming behavior were found in mice with β-catenin over-expression in striatum (See below). These data indicated that Wnt-glycolysis signaling did not contribute to repetitive behavior.

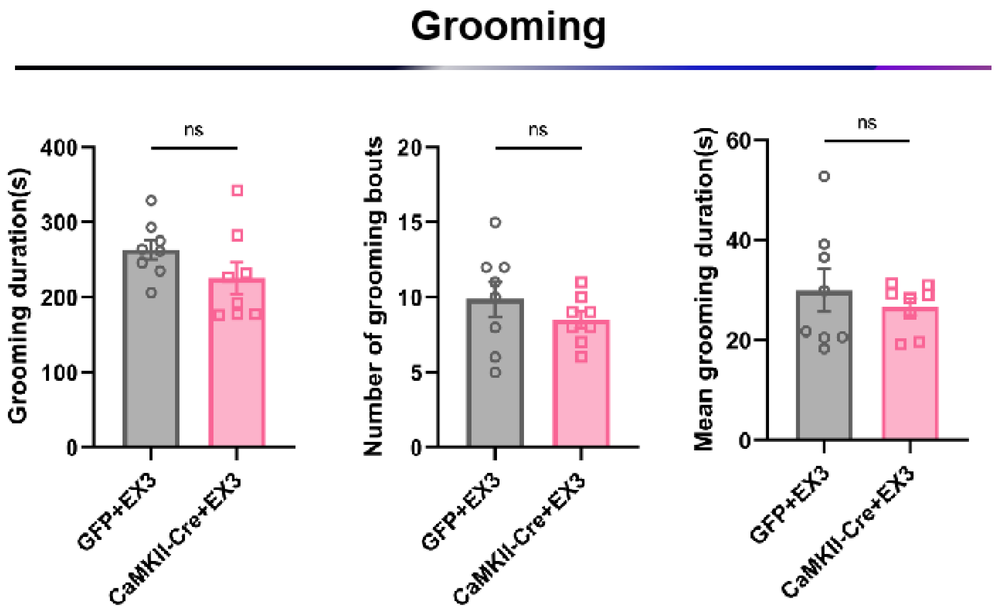

We injected AAV-expressing CaMKII-Cre into striatum of β-catEX3 mice to over-express β-catenin in striatum (mice injected with AAV-GFP was adopted as control). No difference of grooming behavior was found between these mice.

4. The ages of animals used for each experiment were not indicated in the manuscript. This information is necessary to understand the context of individual experiments and should be clearly described accordingly.

Reply:
The information has been added in revised manuscript.

5. This study employed two ASD mouse models to identify convergent molecular mechanism underlying ASD. There is a big difference in axin2/tubulin ratio (Figure 1F vs Figure 1I), ATP level (Figure 2A), and lactate level (Figure 2E vs Figure 2G) between wild type mice of each model. Why is that?

Reply:
Thank you for the question.
For Axin2/tubulin ratio, Figure 1F was derived from primary cultured neurons while Figure 1I was from the ACC tissue. It is reasonable that there was slight difference. To make this clear, we labeled Figure 1F “in vitro” and Figure 1I “in vivo” in the image.
For ATP levels (Figure 2A), the WT mice in left panel were the same littermates of Shank3-KO while the Control mice in right panel had been exposed same amount of saline (as the vehicle control of VPA) at E12.5. Therefore, there may be a slight

difference. Anyway, we remeasured the ATP levels in Figure 2A and included the new data. To reduce confusion, we revised figure labeling as “Con”.
For lactate, we apologize for the big difference presented. The lactic acid in Figure 2G were measured by using fresh samples while that in Figure 2E was obtained from samples stored in -80°C for about 1 week (owing to the influence of COVID-19 at that time). We remeasured the levels of lactic acid in Figure 2E using fresh samples and new data have been added.

Referee #3 (Comments on Novelty/Model System for Author):

Neurologic disorders with social dysfunction are a prominent part of developmental disorders. The WNT signaling system may be linked to 30-40% of ASD and metabolic abnormalities are similarly seen in at least 30% of ASD patients. Thus, this investigation and its findings fills an important gap in the literature. It is further bolstered by findings related to mTOR signaling in autism. As such this manuscript is an important contribution to the autism literature.

All papers have some areas of improvement and here are the reviewer's suggestions:

1) In the Introduction, the last paragraphs should introduce the central hypothesis of the paper and then major aims or questions to be asked by the investigation.

Reply:
Thank you for the suggestion. We have revised Introduction by adding our hypothesis and the major questions to be addressed (Line 8-21, page 5).

2) Under the section: "Wnt signaling over-activation in ACC impairs social function"--please re-word the description of Fig 3 to indicate plainly whether social preference is present or not and whether social novelty is present or not. I think I understand but it is worded unclearly and needs clarification.

Reply:
Thank you. We revised this part as you suggested (Line 2-3 of page 10).

3) Do we know why compound XAV939 has no effect in WT mice?

Reply:
Thank you for the question. In the adult cortex and most brain regions of WT mice, there is no or very low levels of Wnt activity. Therefore, local delivery of XAV939 normally would not has any significant effects in WT mice. If there were some effects of XAV939 on WT mice, that would be associated with the few brain regions where Wnt signaling is maintained at certain level, such as hippocampus. We assessed the effects of ACC delivery of XAV939 in WT mice. The results showed no significant changes of social, repetitive and anxiety-like behaviors (See below).

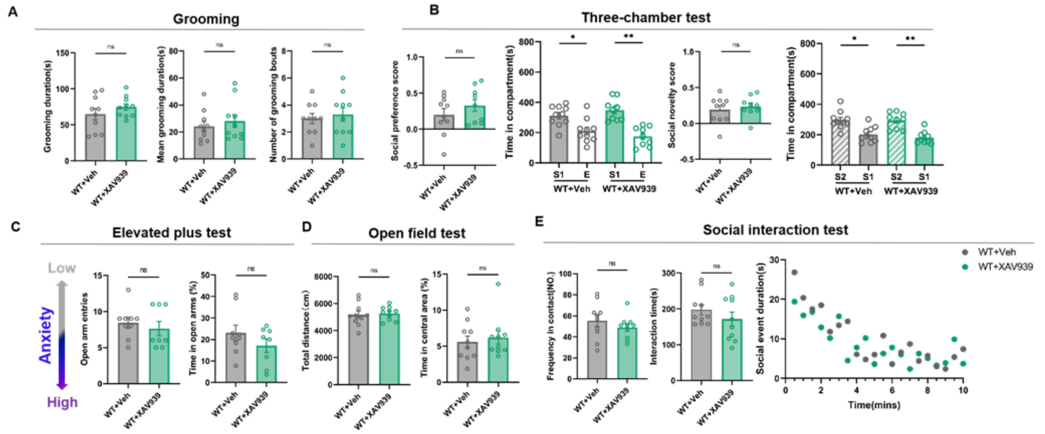

(A) Effects of ACC delivery of XAVA939 on repetitive behavior of WT mice. (B, E) Effects of ACC delivery of XAVA939 on social preference and social interaction of WT mice. (C, D) Effects of ACC delivery of XAVA939 on the anxiety-like behavior of WT mice.

4) While we know the length/age of neurons in mice, it is unclear whether the human neuron experiments replicate that which is seen in vivo and therefore whether Axin2 is a reasonable drug target. The developmental time course of Axin2 interactions in vitro was not studied or at least presented in the manuscript. The authors did show they replicate the mouse data, but when, where, and how they came to this data is unclear. XAV939 could be significantly more toxic at certain ages and that is important to know.

Reply:

Thank you for the comments. We acknowledge that it is hard to precisely replicate the in vivo age of mice by in vitro human neurons, and agree that early interference (particularly at embryonic stage) of Axin2 may be toxic since Wnt signaling is required for the development of multiple tissues. We assessed Axin2/ENO1 interaction in human cells at different stages as you suggested. In human NPCs, there was basal and similar levels of Axin2/ENO1 interaction between WT and Shank3-KO cells. In human neurons, both *Shank3* mutation and VPA-pretreatment resulted in robust increase of Axin2/ENO1 interaction, as compared with corresponding WT and naïve controls. These data demonstrated that enhanced Axin2/ENO1 interaction occurred mainly in ASD human neurons, which was in line with the up-regulation of Wnt-glycolysis signaling from P14 in ASD mice, indicating that Axin2 in postnatal neurons could be a potential therapeutic target. New CO-IP data have been added in Fig. 8C-D, Fig. EV5C.

5) Many genetic factors are prenatal and second trimester events in humans. Although postnatal Axin2 targeting could help ASD, the real question is what happens if you do a prenatal targeting of the Axin2 interactions? What happens?

Reply:
Thank you for the question. Our data showed that abnormal Wnt signaling and glycolysis appeared mainly from P14 in the ACC of both Shank3-KO and VPA-pretreated mice. In addition, enhanced Axin2/ENO1 interaction was found in human Shank3-KO neurons, but not in their corresponding NPCs. These data indicated that postnatal targeting Axin2/ENO1 interaction could be targeted for treating ASD social dysfunction. Prenatal targeting Axin2 may has other side effects, such as affecting organ development owing to the inhibition of Wnt signaling. New data of CO-IP and developmental Wnt-glycolysis have been added in Fig.8C-D, Fig. EV2A-F, Fig. EV2H-K, Fig. EV5C.

6) The authors suggest that nuclear vs cytosolic targeting of beta-catenin signaling may be important? is there other examples to back up this observation?

Reply:
Thank you for this very important question.
Based on our observation, we would prefer to target cytosolic β-catenin signaling for better outcome in preventing ASD social dysfunction, as our data revealed cytosolic Axin2 as a converging point of Wnt signaling and glycolysis in ASD neurons. We do not exclude the possibility that nuclear β-catenin signaling which may directly regulate glycolytic gene expression in other diseases or conditions. As to other examples in stand of our opinion, we would suggest GSK-3β. As another cytosolic β-catenin signaling component, GSK-3β is a good candidate which can regulate glycolysis. In addition, our previous study had demonstrated that over-activating GSK-3β could improve social function in Shank3-KO mice (Front Cell Neurosci. 2019 Oct 23;13:447.). Whether this applies to VPA-induced ASD mice and how GSK-3β modulates glycolysis in ASD neurons are worthy to be studied in detail in the future.

7) In Fig S4, please replace the Western blot of Shank 3. The reviewer cannot tell anything from this blot.

Reply:
The Western-blot has been replaced by a more representative one. The data has been presented in Fig. EV5A in revised manuscript.

8) The reviewer congratulates the authors on a wonderful study and its potential clinical implications. The paper just needs some edits to improve its impact.
Referee #3 (Remarks for Author):
Great study. Should be published after addressing major critiques.

Reply:
Thank you for your positive feedback and very valuable suggestions. We have performed new experiments and added new explanations to address all your concerns.

We sincerely wish our revision is adequate and this paper could be accepted for publication by EMBO Molecular Medicine.

Best regards

Shengxi Wu
Department of Neurobiology and Institute of Neurosciences,
School of Basic Medicine,
Fourth Military Medical University
169 Chang Le Xi Road, Xi'an, Shaanxi 710032, China
Tel: +86-29-84712311
Fax: +86-29-83246270
Email: shengxi@fmmu.edu.cn

23rd Mar 2023

Dear Prof. Wu,

Thank you for sending us your revised manuscript. We have now received the comments from the three referees who agreed to re-review your manuscript. As you will see below, the referees are satisfied with the modifications and think the study is now suitable for publication.

Before we can formally accept your manuscript, we would ask you to address the following editorial-level issues:

1. Please remove the 'Author Contribution' section from the manuscript file.

2. Reference format: Please list up to 10 co-authors of a paper before adding et al. to the reference list.

3. Supplementary file: this file can be removed, and the EV figure legends should be added to the main manuscript after the main figure legends. The callout for "Supplementary Fig. 4A" needs to be changed into "Figure EV4A".

4. Data availability section: Please include the following single sentence in this section- "This study includes no data deposited in external repositories".

5. Supplementary Table 1 (reagent table) should be uploaded as "Table EV1" (excel file). Provide table legend in a separate worksheet within the excel file. Update the callout accordingly.

6. There is a table "Statistics for each figure". Please either incorporate the statistics into the legends or rename it as 'Dataset EV1' (then it should also be called out in the manuscript text and a table legend should be added in a separate worksheet within the excel file).

7. Source data:
- "DNA sequencing file for validation" is not needed, please remove it.
- Please complete the source data checklist
- Files should be upldd as one file per figure with numerical data and western blots zipped.

8. I have deleted the 'Expanded view' section from the manuscript(see attached version).

9. I have slightly modified the synopsis text and 'The paper explained'. Please let me know if they are fine or if you would like to introduce further modifications. Move 'The paper explained' to the main manuscript file.

10. Our data editors have seen the manuscript, and they have made some comments and suggestions that need to be addressed (see attached). Please send back a revised version (in track change mode), as we will need to go through the changes.

11. As part of the EMBO Publications transparent editorial process initiative (see our Editorial at http://embomolmed.embopress.org/content/2/9/329), EMBO Molecular Medicine will publish online a Review Process File (RPF) to accompany accepted manuscripts.

In the event of acceptance, this file will be published in conjunction with your paper and will include the anonymous referee reports, your point-by-point response, and all pertinent correspondence relating to the manuscript. Let us know if you disagree with this and if you want to remove or keep any figures from it prior to publication.

Please note that the Authors checklist will be published at the end of the RPF.

Please submit your revised manuscript within two weeks. I look forward to seeing a revised form of your manuscript as soon as possible.

Kind regards,
Jingyi

Jingyi Hou
Editor
EMBO Molecular Medicine

*** Instructions to submit your revised manuscript ***

*** PLEASE NOTE *** As part of the EMBO Publications transparent editorial process initiative (see our Editorial at https://www.embopress.org/doi/pdf/10.1002/emmm.201000094), EMBO Molecular Medicine will publish online a Review Process File to accompany accepted manuscripts.

In the event of acceptance, this file will be published in conjunction with your paper and will include the anonymous referee reports, your point-by-point response and all pertinent correspondence relating to the manuscript. If you do NOT want this file to be published, please inform the editorial office at contact@embomolmed.org.

When submitting your revised manuscript, please include:

1) a .docx formatted version of the manuscript text (including Figure legends and tables)

2) Separate figure files*

3) supplemental information as Expanded View and/or Appendix. Please carefully check the authors guidelines for formatting Expanded view and Appendix figures and tables at https://www.embopress.org/page/journal/17574684/authorguide#expandedview

4) a letter INCLUDING the reviewer's reports and your detailed responses to their comments (as Word file).

5) The paper explained: EMBO Molecular Medicine articles are accompanied by a summary of the articles to emphasize the major findings in the paper and their medical implications for the non-specialist reader. Please provide a draft summary of your article highlighting
- the medical issue you are addressing,
- the results obtained and
- their clinical impact.
This may be edited to ensure that readers understand the significance and context of the research.
Please refer to any of our published articles for an example.

6) For more information: There is space at the end of each article to list relevant web links for further consultation by our readers. Could you identify some relevant ones and provide such information as well? Some examples are patient associations, relevant databases, OMIM/proteins/genes links, author's websites, etc...

7) EMBO Molecular Medicine now requires a complete author checklist (https://www.embopress.org/page/journal/17574684/authorguide) to be submitted with all revised manuscripts. Please use the checklist as guideline for the sort of information we need WITHIN the manuscript. The checklist should only be filled with page numbers were the information can be found. This is particularly important for animal reporting, antibody dilutions (missing) and exact values and n that should be indicted instead of a range.

8) Every published paper now includes a 'Synopsis' to further enhance discoverability. Synopses are displayed on the journal webpage and are freely accessible to all readers. They include a short stand first (maximum of 300 characters, including space) as well as 2-5 one sentence bullet points that summarise the paper. Please write the bullet points to summarise the key NEW findings. They should be designed to be complementary to the abstract - i.e. not repeat the same text. We encourage inclusion of key acronyms and quantitative information (maximum of 30 words / bullet point). Please use the passive voice. Please attach these in a separate file or send them by email, we will incorporate them accordingly.

You are also welcome to suggest a striking image or visual abstract to illustrate your article. If you do please provide a jpeg file 550 px-wide x 400-px high.

9) A Conflict of Interest statement should be provided in the main text

10) Please note that we now mandate that all corresponding authors list an ORCID digital identifier. This takes <90 seconds to

complete. We encourage all authors to supply an ORCID identifier, which will be linked to their name for unambiguous name identification.

Currently, our records indicate that the ORCID for your account is 0000-0002-3210-9567.

Please click the link below to modify this ORCID:
Link Not Available

11) The system will prompt you to fill in your funding and payment information. This will allow Wiley to send you a quote for the article processing charge (APC) in case of acceptance. This quote takes into account any reduction or fee waivers that you may be eligible for. Authors do not need to pay any fees before their manuscript is accepted and transferred to our publisher.

*Additional important information regarding Figures

Each figure should be given in a separate file and should have the following resolution:
Graphs 800-1,200 DPI
Photos 400-800 DPI
Colour (only CMYK) 300-400 DPI"

Figures are not edited by the production team. All lettering should be the same size and style; figure panels should be indicated by capital letters (A, B, C etc). Gridlines are not allowed except for log plots. Figures should be numbered in the order of their appearance in the text with Arabic numerals. Each Figure must have a separate legend and a caption is needed for each panel.

*Additional important information regarding figures and illustrations can be found at https://bit.ly/EMBOPressFigurePreparationGuideline. See also figure legend preparation guidelines: https://www.embopress.org/page/journal/17574684/authorguide#figureformat

The system will prompt you to fill in your funding and payment information. This will allow Wiley to send you a quote for the article processing charge (APC) in case of acceptance. This quote takes into account any reduction or fee waivers that you may be eligible for. Authors do not need to pay any fees before their manuscript is accepted and transferred to our publisher.

***** Reviewer's comments *****

Referee #1 (Comments on Novelty/Model System for Author):

authors addressed all concerns raised by reviewers.

Referee #1 (Remarks for Author):

authors addressed all concerns raised by reviewers. now i am glad to recommend its acceptance for publication.

Referee #2 (Comments on Novelty/Model System for Author):

The animal models are generally used to study ASD mechanism.

Referee #2 (Remarks for Author):

The authors performed additional experiments and addressed most of my concerns. I have no extra questions.

Referee #3 (Comments on Novelty/Model System for Author):

The reviewer's comment from the previous review can be referenced.

Referee #3 (Remarks for Author):

All of the reviews have been answered and the reviewer has no other comments. Excellent response to all 3 reviewers.

The authors addressed the remaining formatting issues.

3rd Apr 2023

Dear Prof. Wu,

We are pleased to inform you that your manuscript is accepted for publication and is now being sent to our publisher to be included in the next available issue of EMBO Molecular Medicine.

Please read below for additional IMPORTANT information regarding your article, its publication and the production process.

Congratulations on your interesting work,

Zeljko Durdevic

Zeljko Durdevic
Editor
EMBO Molecular Medicine

Follow us on Twitter @EmboMolMed
Sign up for eTOCs at embopress.org/alertsfeeds

*** *** *** IMPORTANT INFORMATION *** *** ***

SPEED OF PUBLICATION
The journal aims for rapid publication of papers, using using the advance online publication "Early View" to expedite the process: A properly copy-edited and formatted version will be published as "Early View" after the proofs have been corrected. Please help the Editors and publisher avoid delays by providing e-mail address(es), telephone and fax numbers at which author(s) can be contacted.

Should you be planning a Press Release on your article, please get in contact with embomolmed@wiley.com as early as possible, in order to coordinate publication and release dates.

LICENSE AND PAYMENT:

All articles published in EMBO Molecular Medicine are fully open access: immediately and freely available to read, download and share.

EMBO Molecular Medicine charges an article processing charge (APC) to cover the publication costs. You, as the corresponding author for this manuscript, should have already received a quote with the article processing fee separately. Please let us know in case this quote has not been received.

Once your article is at Wiley for editorial production you will receive an email from Wiley's Author Services system, which will ask you to log in and will present you with the publication license form for completion. Within the same system the publication fee can be paid by credit card, an invoice, pro forma invoice or purchase order can be requested.

Payment of the publication charge and the signed Open Access Agreement form must be received before the article can be published online.

PROOFS

You will receive the proofs by e-mail approximately 2 weeks after all relevant files have been sent o our Production Office. Please return them within 48 hours and if there should be any problems, please contact the production office at embopressproduction@wiley.com.

Please inform us if there is likely to be any difficulty in reaching you at the above address at that time. Failure to meet our deadlines may result in a delay of publication.

All further communications concerning your paper proofs should quote reference number EMM-2022-17101-V3 and be directed to the production office at embopressproduction@wiley.com.

Thank you,

Zeljko Durdevic
Editor
EMBO Molecular Medicine

EMBO Press Author Checklist

Corresponding Author Name: Shengxi Wu	
Journal Submitted to: EMBO Molecular Medicine	
Manuscript Number: EMM-2022-17101	

USEFUL LINKS FOR COMPLETING THIS FORM
The EMBO Journal - Author Guidelines
EMBO Reports - Author Guidelines
Molecular Systems Biology - Author Guidelines
EMBO Molecular Medicine - Author Guidelines

Reporting Checklist for Life Science Articles (updated January 2022)
This checklist is adapted from Materials Design Analysis Reporting (MDAR) Checklist for Authors. MDAR establishes a minimum set of requirements in transparent reporting in the life sciences (see Statement of Task: 10.31222/osf.io/9sm4x). Please follow the journal's guidelines in preparing your manuscript.
Please note that a copy of this checklist will be published alongside your article.

Abridged guidelines for figures
1. Data
The data shown in figures should satisfy the following conditions:
- the data were obtained and processed according to the field's best practice and are presented to reflect the results of the experiments in an accurate and unbiased manner.
- ideally, figure panels should include only measurements that are directly comparable to each other and obtained with the same assay.
- plots include clearly labeled error bars for independent experiments and sample sizes. Unless justified, error bars should not be shown for technical replicates.
- if n<5, the individual data points from each experiment should be plotted. Any statistical test employed should be justified.
- Source Data should be included to report the data underlying figures according to the guidelines set out in the authorship guidelines on Data Presentation.

2. Captions
Each figure caption should contain the following information, for each panel where they are relevant:
- a specification of the experimental system investigated (eg cell line, species name).
- the assay(s) and method(s) used to carry out the reported observations and measurements.
- an explicit mention of the biological and chemical entity(ies) that are being measured.
- an explicit mention of the biological and chemical entity(ies) that are altered/varied/perturbed in a controlled manner.
- the exact sample size (n) for each experimental group/condition, given as a number, not a range;
- a description of the sample collection allowing the reader to understand whether the samples represent technical or biological replicates (including how many animals, litters, cultures, etc.).
- a statement of how many times the experiment shown was independently replicated in the laboratory.
- definitions of statistical methods and measures:
  - common tests, such as t-test (please specify whether paired vs. unpaired), simple χ2 tests, Wilcoxon and Mann-Whitney tests, can be unambiguously identified by name only, but more complex techniques should be described in the methods section;
  - are tests one-sided or two-sided?
  - are there adjustments for multiple comparisons?
  - exact statistical test results, e.g., P values = x but not P values < x;
  - definition of 'center values' as median or average;
  - definition of error bars as s.d. or s.e.m.

Please complete ALL of the questions below.
Select "Not Applicable" only when the requested information is not relevant for your study.

Materials

Newly Created Materials	Information included in the manuscript?	In which section is the information available? (Reagents and Tools Table, Materials and Methods, Figures, Data Availability Section)
New materials and reagents need to be available; do any restrictions apply?	Not Applicable	

Antibodies	Information included in the manuscript?	In which section is the information available? (Reagents and Tools Table, Materials and Methods, Figures, Data Availability Section)
For **antibodies** provide the following information:<br>- Commercial antibodies: RRID (if possible) or supplier name, catalogue number and or/clone number<br>- Non-commercial: RRID or citation	Yes	The information has been included in supplementary reagent table.

DNA and RNA sequences	Information included in the manuscript?	In which section is the information available? (Reagents and Tools Table, Materials and Methods, Figures, Data Availability Section)
**Short novel DNA or RNA including primers, probes**: provide the sequences.	Yes	The sequences of Shank3 gRNA and the sequencing primers were included in the section of Materials and Methods.

Cell materials	Information included in the manuscript?	In which section is the information available? (Reagents and Tools Table, Materials and Methods, Figures, Data Availability Section)
**Cell lines:** Provide species information, strain. Provide accession number in repository **OR** supplier name, catalog number, clone number, and/**OR** RRID.	Yes	The information has been included in Materials and Methods.
**Primary cultures:** Provide species, strain, sex of origin, genetic modification status.	Yes	The information has been included in Materials and Methods.
Report if the cell lines were recently **authenticated** (e.g., by STR profiling) and tested for mycoplasma contamination.	Not Applicable	

Experimental animals	Information included in the manuscript?	In which section is the information available? (Reagents and Tools Table, Materials and Methods, Figures, Data Availability Section)
**Laboratory animals or Model organisms:** Provide species, strain, sex, age, genetic modification status. Provide accession number in repository **OR** supplier name, catalog number, clone number, **OR** RRID.	Yes	The information has been included in Materials and Methods.
**Animal observed in or captured from the field:** Provide species, sex, and age where possible.	Not Applicable	
Please detail **housing and husbandry conditions**.	Yes	The information has been included in Materials and Methods.

Plants and microbes	Information included in the manuscript?	In which section is the information available? (Reagents and Tools Table, Materials and Methods, Figures, Data Availability Section)
**Plants:** provide species and strain, ecotype and cultivar where relevant, unique accession number if available, and source (including location for collected wild specimens).	Not Applicable	
**Microbes:** provide species and strain, unique accession number if available, and source.	Not Applicable	

Human research participants	Information included in the manuscript?	In which section is the information available? (Reagents and Tools Table, Materials and Methods, Figures, Data Availability Section)
If collected and within the bounds of privacy constraints report on age, sex and gender or ethnicity for all study participants.	Not Applicable	

Core facilities	Information included in the manuscript?	In which section is the information available? (Reagents and Tools Table, Materials and Methods, Figures, Data Availability Section)
If your work benefited from core facilities, was their service mentioned in the acknowledgments section?	Yes	The information has been provided in Materials and Methods.

Design

Study protocol	Information included in the manuscript?	In which section is the information available? (Reagents and Tools Table, Materials and Methods, Figures, Data Availability Section)
If study protocol has been **pre-registered, provide DOI in the manuscript**. For clinical trials, provide the trial registration number **OR** cite DOI.	Not Applicable	
Report the **clinical trial registration number** (at ClinicalTrials.gov or equivalent), where applicable.	Not Applicable	

Laboratory protocol	Information included in the manuscript?	In which section is the information available? (Reagents and Tools Table, Materials and Methods, Figures, Data Availability Section)
Provide DOI OR other citation details if **external detailed step-by-step protocols** are available.	Not Applicable	

Experimental study design and statistics	Information included in the manuscript?	In which section is the information available? (Reagents and Tools Table, Materials and Methods, Figures, Data Availability Section)
Include a statement about **sample size** estimate even if no statistical methods were used.	Not Applicable	
Were any steps taken to minimize the effects of subjective bias when allocating animals/samples to treatment (e.g. **randomization procedure**)? If yes, have they been described?	Yes	The information has been included the Materials and Methods.
Include a statement about **blinding** even if no blinding was done.	Yes	The information has been included the Materials and Methods.
Describe **inclusion/exclusion criteria** if samples or animals were excluded from the analysis. Were the criteria pre-established?<br><br>If sample or data points were omitted from analysis, report if this was due to attrition or intentional exclusion and provide justification.	Not Applicable	
For every figure, are **statistical tests** justified as appropriate? Do the data meet the assumptions of the tests (e.g., normal distribution)? Describe any methods used to assess it. Is there an estimate of variation within each group of data? Is the variance similar between the groups that are being statistically compared?	Yes	The information has been included the Materials and Methods.

Sample definition and in-laboratory replication	Information included in the manuscript?	In which section is the information available? (Reagents and Tools Table, Materials and Methods, Figures, Data Availability Section)
In the figure legends: state number of times the experiment was **replicated** in laboratory.	Yes	The information has been included the figure legend.
In the figure legends: define whether data describe **technical or biological replicates**.	Yes	The information has been included the figure legend.

Ethics

Ethics	Information included in the manuscript?	In which section is the information available? (Reagents and Tools Table, Materials and Methods, Figures, Data Availability Section)
Studies involving **human participants**: State details of **authority granting ethics approval** (IRB or equivalent committee(s), provide reference number for approval.	Not Applicable	
Studies involving **human participants**: Include a statement confirming that **informed consent** was obtained from all subjects and that the experiments conformed to the principles set out in the WMA Declaration of Helsinki and the Department of Health and Human Services Belmont Report.	Not Applicable	
Studies involving **human participants:** For publication of **patient photos**, include a statement confirming that consent to publish was obtained.	Not Applicable	
Studies involving experimental **animals**: State details of **authority granting ethics approval** (IRB or equivalent committee(s), provide reference number for approval. Include a statement of compliance with ethical regulations.	Yes	The information has been included the Materials and Methods.
Studies involving **specimen and field samples:** State if relevant **permits** obtained, provide details of authority approving study; if none were required, explain why.	Not Applicable	

Dual Use Research of Concern (DURC)	Information included in the manuscript?	In which section is the information available? (Reagents and Tools Table, Materials and Methods, Figures, Data Availability Section)
Could your study fall under dual use research restrictions? Please check biosecurity documents and list of **select agents and toxins** (CDC): https://www.selectagents.gov/sat/list.htm	Not Applicable	
If you used a select agent, is the security level of the lab appropriate and reported in the manuscript?	Not Applicable	
If a study is subject to dual use research of concern regulations, is the name of the **authority granting approval and reference number** for the regulatory approval provided in the manuscript?	Not Applicable	

Reporting

The MDAR framework recommends adoption of discipline-specific guidelines, established and endorsed through community initiatives. Journals have their own policy about requiring specific guidelines and recommendations to complement MDAR.

Adherence to community standards	Information included in the manuscript?	In which section is the information available? (Reagents and Tools Table, Materials and Methods, Figures, Data Availability Section)
State if relevant guidelines or checklists (e.g., **ICMJE, MIBBI, ARRIVE, PRISMA**) have been followed or provided.	Yes	The information has been included the Materials and Methods.
For **tumor marker prognostic studies**, we recommend that you follow the **REMARK** reporting guidelines (see link list at top right). See author guidelines, under 'Reporting Guidelines'. Please confirm you have followed these guidelines.	Not Applicable	
For **phase II and III randomized controlled trials**, please refer to the **CONSORT** flow diagram (see link list at top right) and submit the CONSORT checklist (see link list at top right) with your submission. See author guidelines, under 'Reporting Guidelines'. Please confirm you have submitted this list.	Not Applicable	

Data Availability

Data availability	Information included in the manuscript?	In which section is the information available? (Reagents and Tools Table, Materials and Methods, Figures, Data Availability Section)
Have **primary datasets** been deposited according to the journal's guidelines (see 'Data Deposition' section) and the respective accession numbers provided in the Data Availability Section?	Not Applicable	
Were **human clinical and genomic datasets** deposited in a public access-controlled repository in accordance to ethical obligations to the patients and to the applicable consent agreement?	Not Applicable	
Are **computational models** that are central and integral to a study available without restrictions in a machine-readable form? Were the relevant accession numbers or links provided?	Not Applicable	
If publicly available data were reused, provide the respective **data citations in the reference list**.	Not Applicable	
